# The circadian transcriptome of marine fish (*Sparus aurata*) larvae reveals highly synchronized biological processes at the whole organism level

**DOI:** 10.1038/s41598-017-13514-w

**Published:** 2017-10-11

**Authors:** M. Yúfera, E. Perera, J. A. Mata-Sotres, J. Calduch-Giner, G. Martínez-Rodríguez, J. Pérez-Sánchez

**Affiliations:** 1 Department of Marine Biology and Aquaculture, Instituto de Ciencias Marinas de Andalucía, ICMAN-CSIC, 11519 Cádiz, Spain; 2 Nutrigenomics and Fish Growth Endocrinology, Institute of Aquaculture Torre de la Sal, IATS-CSIC, 12595 Castellón, Spain; 3 Present Address: CONACYT-Nutrición y Fisiología Digestiva. Instituto de Investigaciones Oceanológicas, Universidad Autónoma de Baja California (UABC), 22860 Ensenada, Baja California Mexico

## Abstract

The regulation of circadian gene expression remains largely unknown in farmed fish larvae. In this study, a high-density oligonucleotide microarray was used to examine the daily expression of 13,939 unique genes in whole gilthead sea bream (*Sparus aurata*) larvae with fast growth potentiality. Up to 2,229 genes were differentially expressed, and the first two components of Principal Component Analysis explained more than 81% of the total variance. Clustering analysis of differentially expressed genes identified 4 major clusters that were triggered sequentially, with a maximum expression at 0 h, 3 h, 9–15 h and 18-21 h zeitgeber time. Various core clock genes (*per1, per2*, *per*3*, bmal1, cry1, cry*2*, clock*) were identified in clusters 1–3, and their expression was significantly correlated with several genes in each cluster. Functional analysis revealed a daily consecutive activation of canonical pathways related to phototransduction, intermediary metabolism, development, chromatin remodeling, and cell cycle regulation. This daily transcriptome of whole larvae resembles a cell cycle (G1/S, G2/M, and M/G1 transitions) in synchronization with multicellular processes, such as neuromuscular development. This study supports that the actively feeding fish larval transcriptome is temporally organized in a 24-h cycle, likely for maximizing growth and development.

## Introduction

The evolution of many organisms has been driven by circadian rhythms to adapt to periodic events in their external environments. These self-sustained and entrainable 24-h rhythms begin in the early stages of development and rely on tight regulation of gene expression. Fish are the most diverse vertebrate group and have evolved in quite different habitats (e.g., freshwater *vs*. sea water, tropical *vs*. temperate, diurnal *vs*. nocturnal), coordinating metabolic processes in a timely fashion^1^. This metabolic coordination is particularly relevant for farmed fish species, whose growth, health, and well-being may rely on synchronization between endogenous rhythms and external clues imposed by production practices. Therefore, information on daily transcriptome organization for a given species would enable further comparison of farming conditions, evaluate the adaptation capacity to changing environments, identify reliable markers of health and performance, and refine feeding practices by matching diet composition or the time of diet delivery to the temporal requirements of the organism. Thus far, massive gene expression analyses addressing clock-driven transcription in fish larvae have mostly been conducted in zebrafish^2,3^. Both microarray and RNA-Seq gene expression profiling of whole-body larvae of other teleostean fishes have been used to unravel key issues of development^4,5^, domestication^6^, and the effects of different environmental factors such as pollutants, diet, stress, and infection^7–12^ but not to elucidate overall daily transcriptomic organization.

Daily gene expression is driven, although not exclusively, by circadian clocks. The current vertebrate circadian model involves a positive core loop with heterodimer BMAL1/CLOCK that transactivates promoters with circadian clock-responsive elements such as E-box (e.g., in period genes, *per*) and triggers different transcriptional cascades^13^. The negative core loop of the model includes PER and cryptochromes (CRY), which after gradual accumulation can suppress BMAL1/CLOCK transcriptional activity^14^. PER and CRY are progressively phosphorylated and targeted for degradation, allowing reactivation of *clock*/*bmal1* ^15^. Additional ancillary loops drive the alternate activation and repression of *bmal1* ^16^, *per* and *nr1d1* (*rev-erbα*) genes^17^. Significant advancements have been made in understanding the functioning of these clock components in commercially important fish species. The available studies have focused mostly on juvenile or adult stages and on specific tissues such as the pineal^18^ and liver^19^ in salmon, the pineal in the European sea bass^18^, and the brain and liver in the gilthead sea bream (*Sparus aurata)* ^20^. However, a study in *S. aurata* demonstrated that, as in other vertebrates, there are differences in the entrainment of central and peripheral clocks, being the liver clock regulated by feed rather than by light cycles^20^. Different to juveniles, larvae from farmed fish are cultured under constant feed availability and, in theory, with both central and peripheral clocks in phase as reported in higher vertebrates^21^. Actually, under these conditions, whole *S. aurata* larvae exhibit clear circadian rhythms in clock genes expression, being the expression of *bmal1*/*clock* and voluntary ingestion closely correlated^22^. In addition, most circadian clock studies on early stages of development of cultured fish have focused on the expression of a discrete number of clock genes^22–24^, and there is still little information on the overall metabolic output of the molecular clock. Thus, by analyzing clock components and transcriptomic variations in entire fish larvae, a better representation of the temporal organization of metabolism can be achieved. This approach has been used in early (5 days post-fertilization) zebrafish larvae^2^, but transcriptomic variations in more advanced and actively feeding fish larvae have not yet been examined, even though feeding exerts a prominent effect on fish metabolism (e.g., up to a 136% increase in metabolic rate)^25^.

The present study aims to depict the daily transcriptomic changes of the feeding larval stage of *S. aurata* under culture conditions and their relationship with the circadian clock. This is a perciform fish of high value among temperate farmed species of European aquaculture. Since fish larvae of *S. aurata* exhibit a remarkably high growth rate, which can reach 12–15% day^−1^ ^26^, we hypothesized that the metabolism of actively feeding larvae of this species has a highly synchronized and time-based organization. To test this hypothesis, the *S. aurata* nucleotide database (www.nutrigroup-iats.org/seabreamdb)^27^ was updated with sequences from pyrosequencing of 454 libraries of larval origin^28^, and a specific high-density oligonucleotide microarray was constructed to examine, over a single day, the expression profile of more than 13,900 unique genes in whole larvae kept under a light/dark (LD) cycle with continuous feed availability. This gene expression analysis demonstrated the coordinated daily progression of various cellular and metabolic processes and highlighted the putative role of the circadian clock in the organization of daily metabolism and growth in whole fish larvae.

## Results

### Several genes are sequentially expressed during the daily cycle

**Figure 1:**
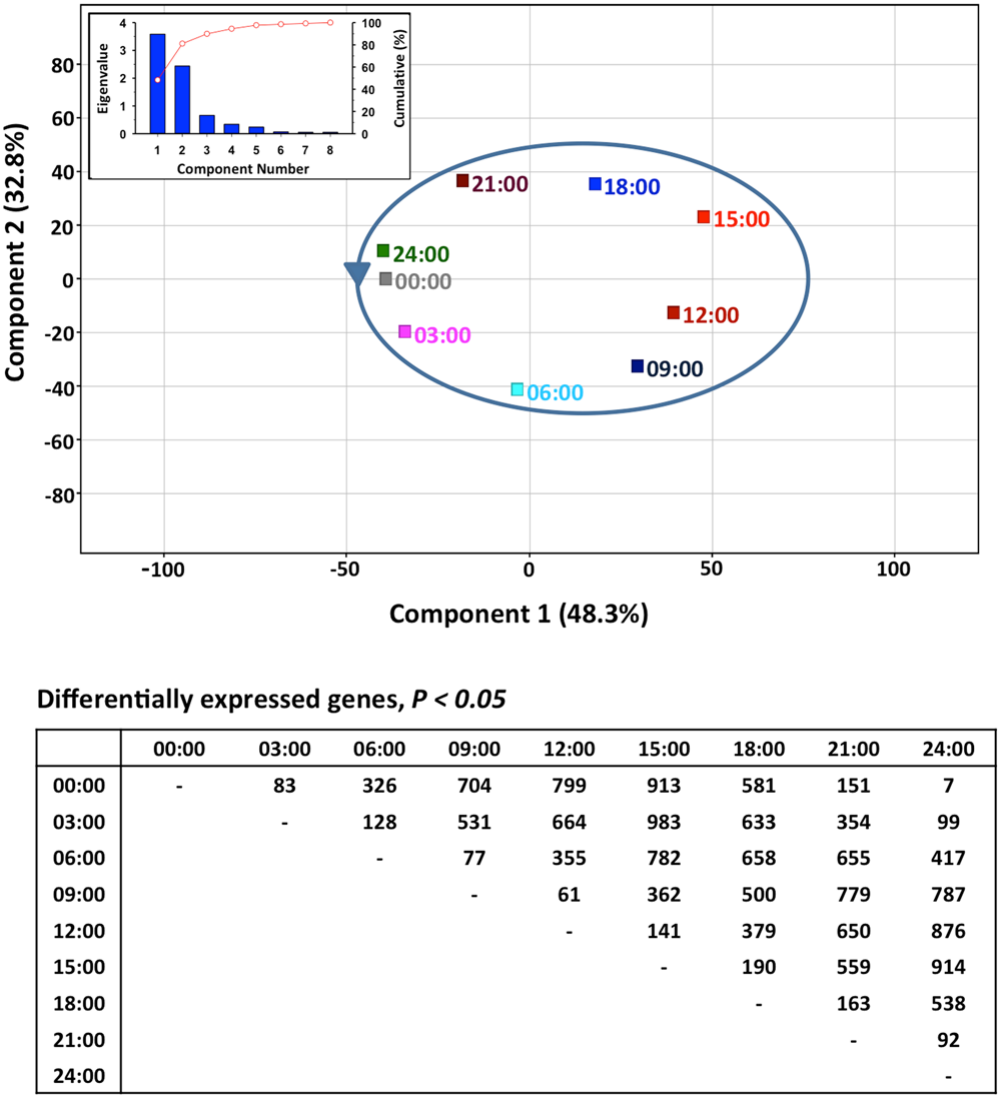
Principal component analysis of larval transcriptome at various time points. Insert is a scree plot of the principal component analysis, showing eigenvalues (blue bars) and cumulative variability explained (red points) against the number of the principal component. The number of differentially expressed genes among experimental groups was determined by one-way ANOVA (corrected P-value < 0.05, Benjamini-Hochberg).

To investigate whether the whole fish larvae transcriptome exhibits a daily pattern, 30-day-old *S. aurata* larvae were sampled every 3 h during a 24-h cycle under continuous feed availability and 12 h light: 12 h dark photoperiod. Then, a customized high-density oligo-microarray was used to profile the expression of 13,939 unique genes of *S. aurata*. One-way ANOVA showed that 2,229 genes were differentially expressed throughout the day (Supplementary Table S1). Principal Component Analysis (PCA) of differentially expressed genes showed a cyclic distribution of the groups along two components that accounted for 81% of the total variance (Fig. 1). Of note, minimal transcriptome differences (7 genes) were found when the comparison was made between fish sampled at 0 h and 24 h zeitgeber time.

### Differentially expressed genes are grouped into four clusters

**Figure 2:**
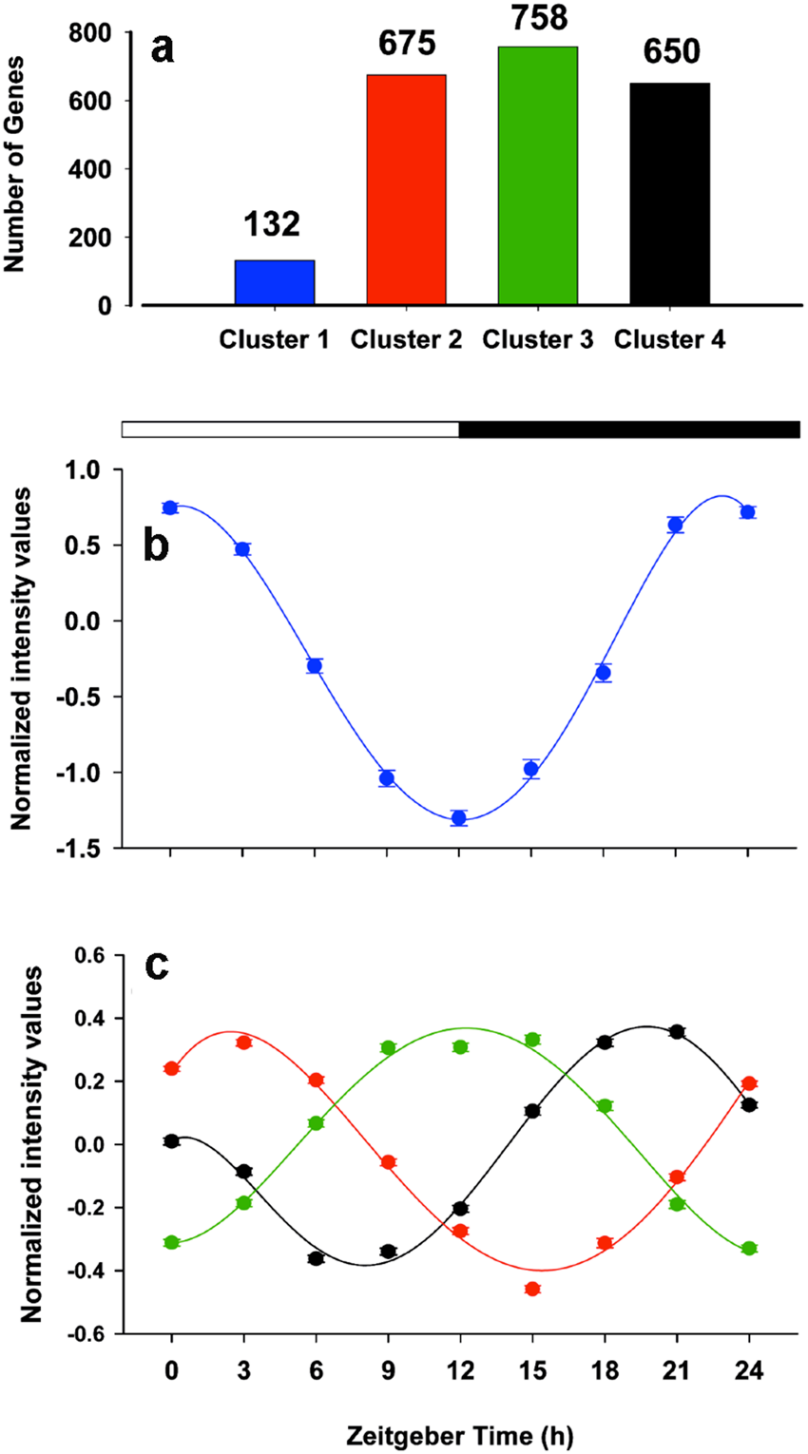
K-means clustering of differentially expressed genes. (**a**) Number of genes in each cluster. (**b**) Average expression profile of cluster 1 genes. (**c**) Average expression profiles of cluster 2 (red), cluster 3 (green) and cluster 4 (black). At each time point, the mean ± SEM of six individuals is represented.

The k-means clustering of differentially expressed genes identified 4 major clusters with a sequential expression profile (Fig. 2a). The entire sets of genes included in each cluster are listed in Supplementary Table S1, with fold-change expression values referring to fish at 24 h zeitgeber time. Cluster 1 comprised the lowest number of genes (132), although the magnitude of response was higher than in the other clusters, with a peak of expression at 0 h zeitgeber time and the minimum 12 h later (Fig. 2b). Clusters termed 2, 3 and 4 contained 675, 758 and 650 genes, respectively; they also showed a circadian pattern of expression with intensity peaks at 3 h (cluster 2), 9–15 h (cluster 3) and 18–21 h (cluster 4) zeitgeber time (Fig. 2c).

### Daily activation of canonical pathways of intermediary metabolism, development and cell cycle

**Figure 3:**
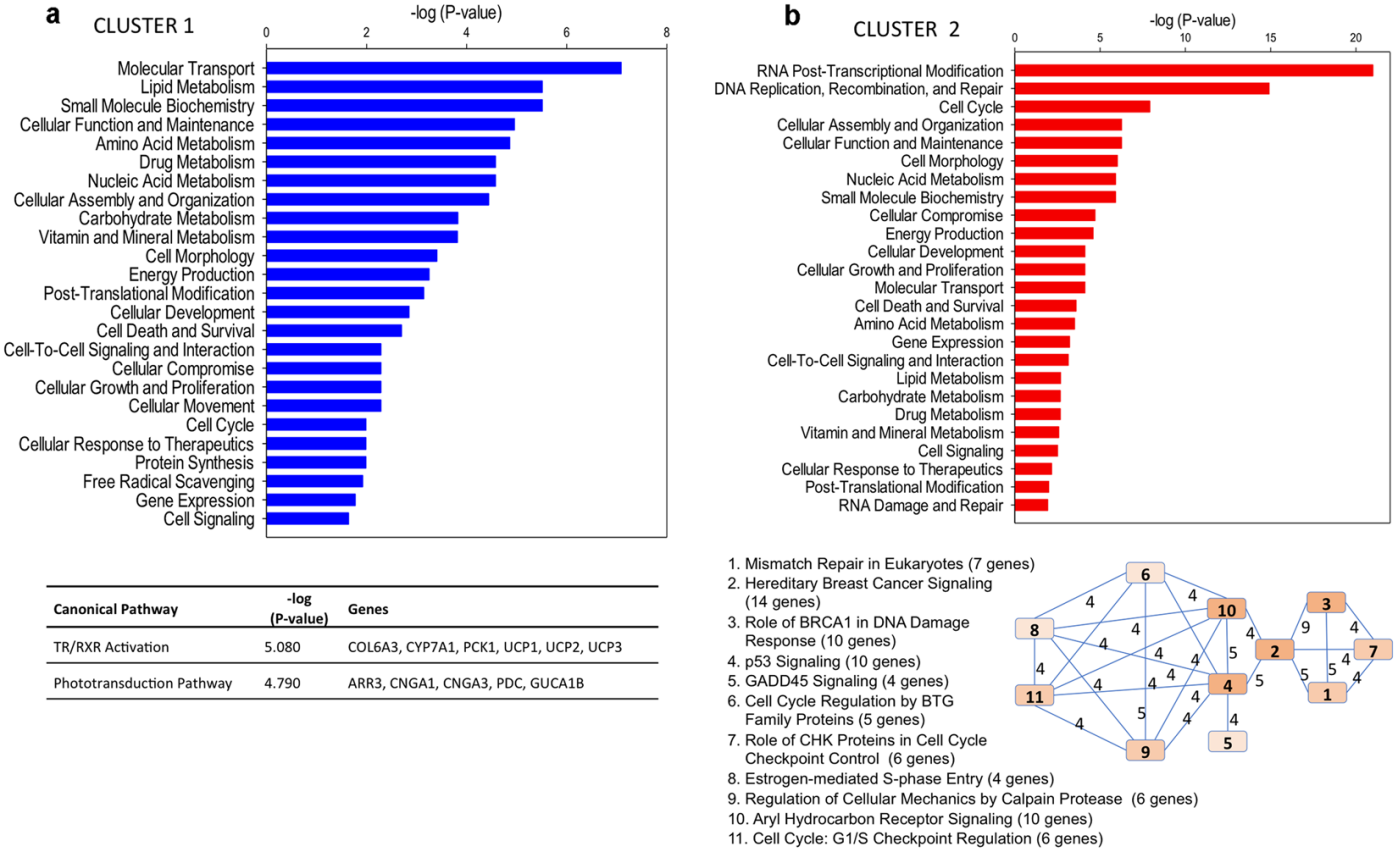
Functional characterization of genes present in cluster 1 and 2 by Ingenuity Pathway Analysis. (**a**) Top represented molecular and cellular functions and top represented canonical pathways (below the graph) in cluster 1. (**b**) Top represented molecular and cellular functions in cluster 2, and overlapping analysis of related canonical pathways (below the graph). Number of common genes between pathways is represented on the connection lines. Pathways are numbered according to their significance value from lower (more significant) to higher, and the color grading of boxes is representative of the number of genes in each pathway.

**Figure 4:**
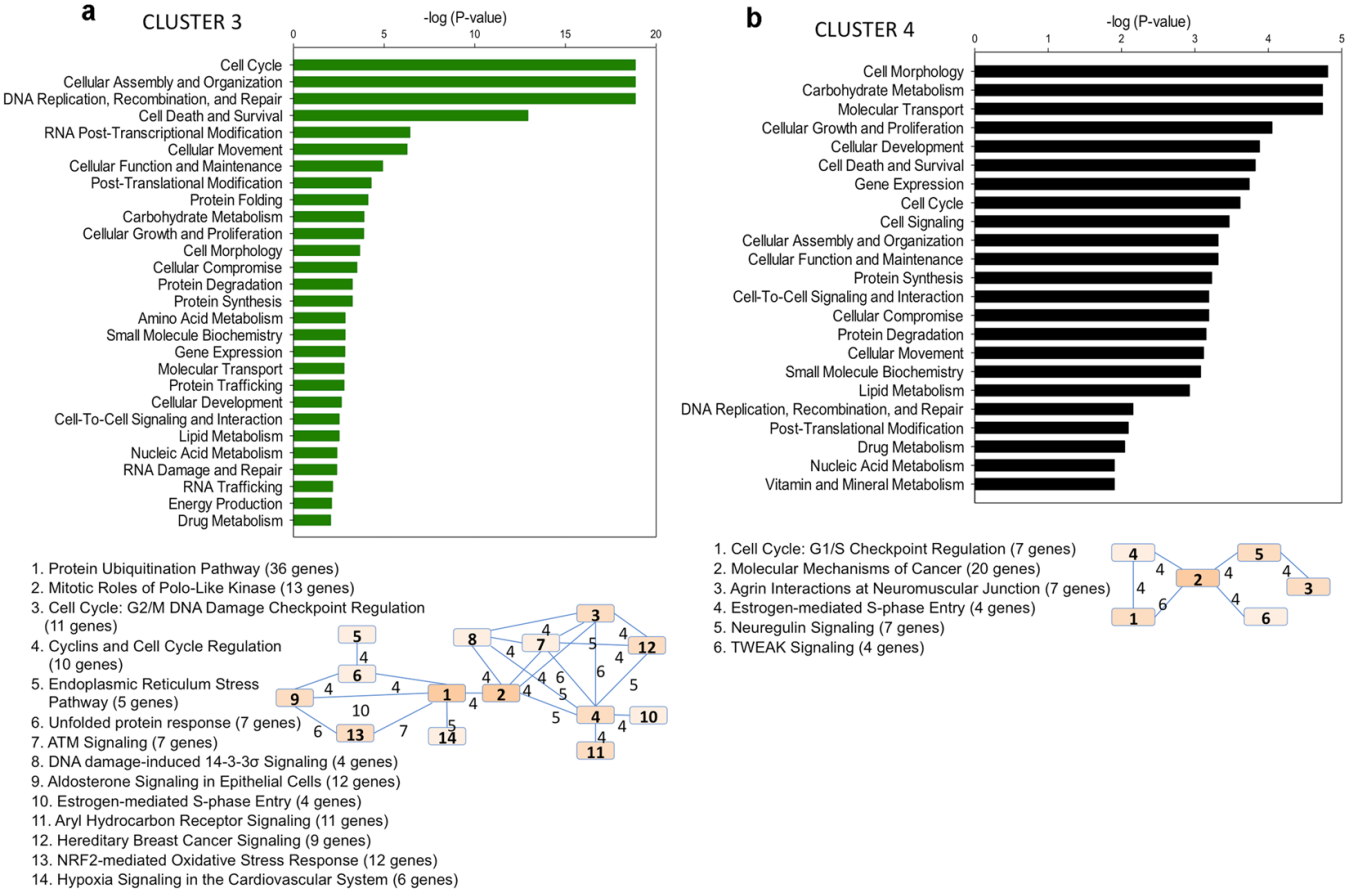
Functional characterization of genes present in cluster 3 and 4 by Ingenuity Pathway Analysis. (**a**) Top represented molecular and cellular functions, and overlapping analysis of related canonical pathways (below the graph) in cluster 3. (**b**) Top represented molecular and cellular functions, and overlapping analysis of related canonical pathways (below the graph) in cluster 4. Number of common genes between pathways is represented on the connection lines. Pathways are numbered according to their significance value from lower (more significant) to higher, and the color grading of boxes is representative of the number of genes in each pathway.

Ingenuity Pathway Analysis (IPA) software was used to gain insight into the biological functions and pathways that were most significant in the different detected clusters. Up to 92.6% (2,052 genes) of differentially expressed genes were eligible for pathway analysis in the IPA software. Regarding molecular and cellular functions, the most significant for cluster 1 were “molecular transport”, “lipid metabolism”, “amino acid metabolism”, “nucleic acid metabolism”, “carbohydrate metabolism”, and “vitamin and mineral metabolism” (Fig. 3a). Notably, the most significant canonical pathways in this cluster were Thyroid hormone receptor and retinoid X receptor (TR/RXR) activation, represented by collagen alpha-3(VI) chain, cholesterol 7 alpha-monooxygenase (*cyp7a1*), phosphoenol pyruvate carboxykinase 1, and the mitochondrial uncoupling proteins (*ucp1, ucp2*, and *ucp3*). In addition, Phototransduction was significantly over-represented by five genes: arrestin 3, cyclic nucleotide-gated channel alpha 1 and 3, phosducin, and guanylatecyclase activator 1B. In cluster 2, the molecular functions “RNA post-transcriptional modification” and “DNA replication, recombination, and repair” were clearly the most significant (Fig. 3b), and overlapping analysis of associated canonical pathways identified a group of 30 related genes (Table 1), which were mainly involved in repair response to DNA damage and cell cycle regulation. “Cell cycle” and “DNA replication, recombination, and repair” were also among the top molecular and cellular functions of cluster 3, together with “cellular assembly and organization” (Fig. 4a). In this cluster, significant overlapping of canonical pathways identified 72 genes (Table 2), mostly related to unfolded protein responses, oxidative stress, and regulation of cell cycle. In contrast, there were no clearly prominent molecular and cellular functions in cluster 4 (Fig. 4b), and overlapping analysis only resulted in six related pathways with 26 overlapping genes (Table 3), including those of the cell cycle and signaling processes related to development (Neuregulin signaling, Agrin interactions at neuromuscular junction, and TWEAK-TNF-like weak inducer of apoptosis signaling).

**Table 1:** Genes comprised in the overlapping analysis of associated canonical pathways of cluster 2.

Clone	Gene description	Canonical pathway
C2_36119	Breast cancer type 2 susceptibility protein homolog	2 3
C3_c26352	Calpain-2 catalytic subunit	9
C3_c55605	Calpain-8-like	9
C2_8602	Cyclin-dependent kinase 2	4 5 6 7 8 9 10 11
C2_7311	Cyclin-dependent kinase 4	2 4 5 6 8 9 10 11
C2_13531	DNA endonuclease RBBP8	3
FM148633	DNA mismatch repair protein Msh2	1 2 3
C2_11812	DNA mismatch repair protein Msh6	3
C2_18591	DNA polymerase alpha catalytic subunit	10
C2_10309	DNA polymerase delta catalytic subunit	1
C2_12300	DNA repair protein complementing XP-C cells	2
C2_19297	Fanconi anemia group D2 protein	2 3
C2_39274	G1/S-specific cyclin-D1	2 4 5 6 8 9 10 11
C2_1122	Guanine nucleotide-binding protein-like 3	4 11
C2_4447	Hypoxia-inducible factor 1-alpha	4
C2_35678	Microsomal glutathione S-transferase 2	10
C2_4674	Nocturnin	6
C2_2183	Nuclear factor erythroid 2-related factor 2	10
C2_2403	Nucleophosmin	2
C2_3598	P21-activated protein kinase-interacting protein 1-like	11
C2_18561	Phosphatidylinositol 3-kinase regulatory subunit alpha	2 4
C2_228	Proliferating cell nuclear antigen	1 4 5 7
C2_39921	Protein-glutamine gamma-glutamyltransferase 2	10
C3_c18240	Proto-oncogene c-Fos-like	10
C2_2839	Replication factor C subunit 3	1 2 3 7
C2_9764	Replication factor C subunit 5	1 2 3 7
C2_6841	Replication protein A 70 kDa DNA-binding subunit	1 2 3 7
C2_15396	Retinoblastoma-associated protein	2 3 4 6 8 9 10 11
C2_4245	Ribonucleoside-diphosphate reductase subunit M2 B	4
C2_11938	Serine/threonine-protein kinase Chk1	2 3 4 7 10

The clone code for each gene in the Nutrigroup database (www.nutrigroup-iats.org/seabreamdb) is indicated. Canonical pathway number correspondence is stated in Fig. 3b.

**Table 2:** Genes comprised in the overlapping analysis of associated canonical pathways of cluster 3.

Clone	Gene description	Canonical pathway
C2_299	14-3-3 protein beta/alpha-1	3
C2_4264	26 S protease regulatory subunit 4	1
C2_3002	26 S protease regulatory subunit 7	1
C2_514	26 S protease regulatory subunit 8	1
C2_2728	26 S proteasome non-ATPase regulatory subunit 1	1
C2_1006	26 S proteasome non-ATPase regulatory subunit 3	1
C2_3430	26 S proteasome non-ATPase regulatory subunit 4	1
C2_364	26 S proteasome non-ATPase regulatory subunit 7	1
C2_58026	26 S proteasome non-ATPase regulatory subunit 8	1
C2_14888	78 kDa glucose-regulated protein	1 5 6 9
C2_18402	Actin	13
C2_3814	Aldehyde dehydrogenase family 9 member A1	11
C2_7839	Aurora kinase A-A	3
C2_1023	Calreticulin	5 6
C2_997	Catalase (CAT)	13
C2_7899	Cell division cycle protein 20 homolog	1 2
C2_30599	Cell division cycle protein 23 homolog	1 2
C2_1877	Chromobox protein homolog 5	7
C2_4302	Cyclin-A2	4 10 11
C2_75402	Cyclin-dependent kinase 1-B	2 3 4 7 8 10 12
C2_1055	Cyclin-dependent kinase inhibitor 1	3 4 7 10 11 12
C2_1862	Cyclin-dependent kinases regulatory subunit 1	3
C2_3866	DNA-directed RNA polymerase II subunit RPB7	12
C2_7938	DNA-directed RNA polymerase II subunit RPB9	12
C2_213	DnaJ homolog subfamily A member 1	1 9 13
C2_2999	DnaJ homolog subfamily B member 11	1 9 13
C2_10236	DnaJ homolog subfamily C member 10	1 9 13
C2_5322	DnaJ homolog subfamily C member 17	1 9 13
C2_4773	DnaJ homolog subfamily C member 3	1 5 6 9 13
C2_45195	Double-strand break repair protein MRE11A	7 12
C2_10928	E3 ubiquitin-protein ligase UBR1	1
C2_1490	Endoplasmin (GRP-94)	1 2 5 6 9 11 14
C2_3801	G2/mitotic-specific cyclin-B1	2 3 4 7 8 12
C2_11108	G2/mitotic-specific cyclin-B2	2 3 4 7 8
C2_18447	G2/mitotic-specific cyclin-B3	2 3 4 7 8
C2_117130	Glutathione S-transferase A	11 13
C2_15999	Heat shock 70 kDa protein 1	1 6 9
C2_5798	Heat shock protein beta-8	1 9 13
C2_4132	Heat shock protein HSP 90-alpha 1	1 2 9 11 14
C2_2149	Heme oxygenase	13 4 12
C2_14642	Histone deacetylase 3	4 12
FP337424	Kinesin-like protein KIF11-B	2
C2_10006	Mediator of RNA polymerase II transcription subunit 20	1
C2_7344	NEDD8	11
C2_45763	Nuclear receptor coactivator 7	11
C2_16956	Nuclear receptor subfamily 0 group B member 2	11
C2_6115	Polyubiquitin-C	1 12
C2_276	Proteasome subunit alpha type-1	1
C2_89	Proteasome subunit alpha type-5	1
C2_979	Proteasome subunit alpha type-6	1
C2_99283	Proteasome subunit beta type-1	1
C2_4220	Proteasome subunit beta type-2	1
C2_909	Proteasome subunit beta type-7	1
C2_33651	Protein aurora borealis	3
C2_3894	Protein disulfide-isomerase	6 14
C2_3572	Protein regulator of cytokinesis 1	2
C2_36036	Serine/threonine-protein kinase PLK1	2 3
C2_17621	Serine/threonine-protein kinase PLK4	2
C2_17492	Serine/threonine-protein kinase TAO3	5
C2_1685	Solute carrier family 12 member 1	9
C2_21997	Solute carrier family 12 member 2	9
C2_2037	Transcription factor Dp-1	4 10 11
C2_11275	Transforming growth factor beta-3	4 11
C2_15562	Transitional endoplasmic reticulum ATPase	6 13
C2_3614	U4/U6.U5 tri-snRNP-associated protein 2	1
C2_1964	Ubiquitin carboxyl-terminal hydrolase 14	1 13
C2_21971	Ubiquitin carboxyl-terminal hydrolase 28	1
C2_24706	Ubiquitin carboxyl-terminal hydrolase 3	1
C2_3488	Ubiquitin-conjugating enzyme E2 C	1 14
C2_1905	Ubiquitin-conjugating enzyme E2 G1	1 14
C2_3425	Ubiquitin-conjugating enzyme E2 S	1 14
C2_68747	Wee1-like protein kinase	2 3 4 12

The clone code for each gene in the Nutrigroup database (www.nutrigroup-iats.org/seabreamdb) is indicated. Canonical pathway number correspondence is stated in Fig. 4a.

**Table 3:** Genes comprised in the overlapping analysis of associated canonical pathways of cluster 4.

Clone	Gene description	Canonical pathway
C2_5203	Acetylcholine receptor subunit alpha	3
C2_34121	Adenylate cyclase type 7	2
C2_5274	Baculoviral IAP repeat-containing protein 4	2 6
C2_64743	Bone morphogenetic protein receptor type-1B	2
C2_3535	Caspase-3	2 6
C2_8280	Caspase-9	2 6
C2_14042	G1/S-specific cyclin-D2	1 2
C2_91393	G1/S-specific cyclin-E1	1 2 4
C2_99915	Insulin receptor substrate 1	2
C2_8684	Integrin alpha-5	2 3 5
C3_c11836	Laminin subunit beta-1-like	3
C2_96193	Mothers against decapentaplegic homolog 9	2
C2_10260	M-phase inducer phosphatase 1	1 2 4
C2_76810	Muscle, skeletal receptor tyrosine-protein kinase	3
C2_12647	NF-kappa-B inhibitor epsilon	2 6
C2_5958	Nicastrin	2
C2_28111	Pro-neuregulin-1, membrane-bound isoform	1 3 5
C3_c57938	Pro-neuregulin-4, membrane-bound isoform-like	3 5
C2_4242	Protein max	1 2
C2_7590	Ral guanine nucleotide dissociation stimulator	2
C2_7026	Ras-related protein R-Ras2	2 3 5
C2_38744	Rho-related GTP-binding protein RhoU	2
C2_12356	Ribosomal protein S6 kinase beta-2	5
C2_40491	Serine/threonine-protein kinase D3	2 5
C2_10954	Transcription factor E2F4	1 2 4
C3_lrc4088	Transcriptional regulator Myc-1-like	1 2 4 5

The clone code for each gene in the Nutrigroup database (www.nutrigroup-iats.org/seabreamdb) is indicated. Canonical pathway number correspondence is stated in Fig. 4b.

### Expression of several genes in different clusters is correlated with core clock genes

**Figure 5:**
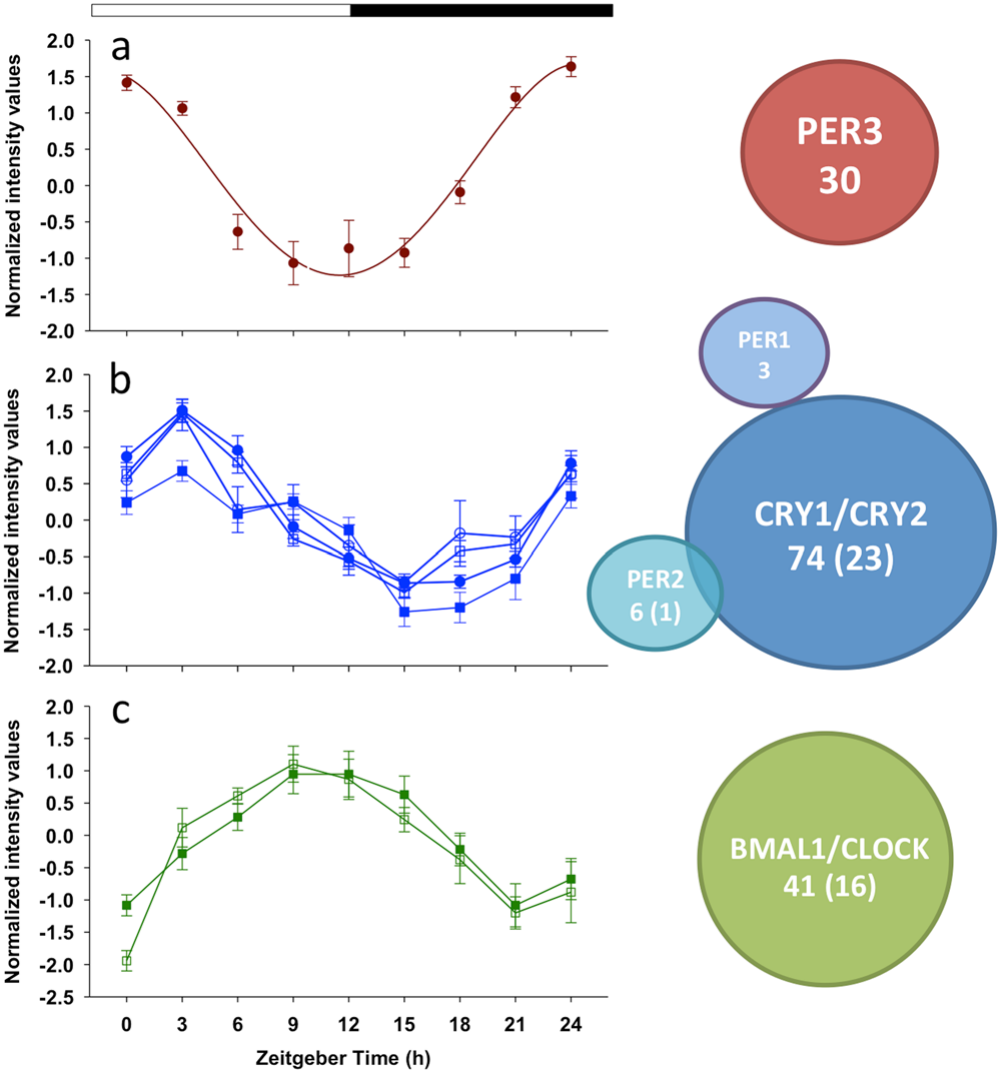
Correlation analysis of differentially expressed genes. Venn diagram (at the right) showing the number of genes significantly correlated (Spearman coefficient > 0.95) to each clock gene in their respective cluster (red for cluster 1, blue for cluster 2, green for cluster 3). In diagrams representing two clock genes, the number in parenthesis indicates the number of genes significantly correlated to both clock genes. (**a**) Average expression profiles (mean ± SEM) of cluster 1 genes significantly correlated to *per3*. (**b**) Average expression profiles (mean ± SEM) of cluster 2 genes significantly correlated to *cry1* (blue circles), *cry2* (white squares), *per1* (blue squares) and *per2* (white circles). (**c**) Average expression profiles (mean ± SEM) of cluster 3 genes significantly correlated to *bmal1* (white squares) and *clock* (green squares).

Seven genes in clusters 1–3 were unequivocally identified as core clock genes. These were the circadian protein homolog 3 (*per3*) in cluster 1; *per1*, *per2* and cryptochromes 1 and 2 (*cry1* and *cry2*) in cluster 2; and aryl hydrocarbon receptor nuclear translocator-like (*bmal1*) and circadian locomotor output cycles kaput (*clock*) in cluster 3. We next sought to establish whether the expression of genes within each cluster may be related to the expression pattern observed for these core clock genes. Interestingly, correlation analysis (Spearman coefficient > 0.95) showed that several genes in clusters 1, 2 and 3 shared the expression dynamics of the identified core clock genes within the corresponding cluster. Figure 5 shows the number of related genes for each clock gene and their average expression profiles. The entire list of significantly correlated genes to these clock genes and their normalized intensity values is provided as a supplemental material (Supplementary Table S2). Nine differentially expressed genes, including three clock genes, covering a wide range of hybridization intensities and fold-change variations were chosen for real-time qPCR analysis, and results were consistent (r = 0.87) with those of the microarray analysis (Supplementary Fig. S1).

## Discussion

While the use of whole-body fish larvae in microarray studies was suggested to be a limiting factor because some tissue-specific effects may be buried by overall gene expression^29^, we found significant daily variations in the expression of both genes expressed in poorly represented tissues (e.g., pineal gland, retina) and those ubiquitously expressed. Additionally, the number of genes detected to have a significant variation through the day, after false positive correction, was relatively high (2,229) compared to those found by other microarrays to vary through ontogeny (~200)^4^ or in response to nutritional programming interventions (924–1,787)^9^. This may result from the remarkable variation in fish daily physiology, and from the update of the nutrigroup-IATS *S. aurata* nucleotide database that yields a powerful microarray tool with approximately 14,000 unique sequences, specifically enriched in actively expressed genes of the intestine and whole-larvae tissues. This represents a significant improvement over previous *S. aurata* arrays used to wide-underline gene expression in fish challenged with nutritional and environmental stressors^30–33^. Noteworthy, all genes present in this new microarray were annotated, and a single probe was designed for each gene. This might avoid potential deviation due to over-representation of multiple probes for a unique transcript and likely contributed to the reliable and clear analytical results, especially when statistical and functional analyses were envisaged. A first glimpse of the differential transcriptomic expression at each sampling point by PCA showed the sequential displacement of each sampling point along the two components that explained 81% of the whole variance, resulting in the striking figure of a 24-h circular cycle (Fig. 1). The shortest distances were observed between successive samples, while the highest distances were found between samples taken with a 12 h difference. This pattern clearly reveals that several genes are expressed sequentially along the day cycle in the entire larva.

### Daily transcriptome organization in the growing larvae

Clustering analysis revealed the existence of 4 clusters of genes differentially expressed throughout the day (Fig. 2). Temporal and functional analysis of the first cluster, referred to as cluster 1, suggested that phototransduction genes are increasingly expressed during the dark period, likely as preparation for the next light phase. Concomitantly, we observed the up-regulation of genes involved in the TR/RXR activation pathway, in agreement with the role of the thyroid hormone (TH) cascade in light signal transduction^34^. TH and its receptors also affect a wide range of metabolic processes. In this regard, the expression of key genes related to lipid and carbohydrate metabolism were particularly enhanced during the night (Fig. 3a). This is the case of CYP7A1, which catalyzes the first reaction in cholesterol catabolism for bile acid synthesis^35^ and PCK1, which is a rate-controlling step of gluconeogenesis and hepatic glucose output^36^. TH action in vertebrates is also associated with changes in metabolic efficiency, modulating the up-regulation of mitochondrial uncoupling proteins (UCPs)^37,38^. This close association was evidenced for *ucp1* and *ucp2–3*, which were all included in cluster 1 (Fig. 3a). Previous results in *S. aurata* revealed that *ucp2* and *3* are up-regulated under feed restriction in aerobic muscle tissues or with aging or nutrient deficiencies in the glycolytic skeletal muscle^39,40^. This metabolic feature would reflect an increased flux of fatty acids towards skeletal muscle, which might also occur in larvae during overnight fasting and the first hours after the light onset. Hence, cluster 1 could be considered a metabolic regulator, considering its temporal pattern, high amplitude of expression, and the inclusion of TR/RXR activation pathway genes.

Cluster 2 also included genes with key roles in both direct light response (e.g., *opn4*) and transmission/amplification of the visual signal (e.g., *pde*) (Supplementary Table S1), which may act in concert with phototransduction genes from cluster 1. However, cluster 2 genes remained actively expressed 3 h after lights were turned on (Fig. 2c), suggesting that they may play a role in light entrainment. This is supported by the presence of the clock genes involved in light entrainment, *per2* ^41^ and *cry1* ^42^, and other light-responsive genes in retinal ganglions (*opn4*)^43^ and pineal gland (e.g., *pinopsin*)^44^ (Supplementary Table S1). Functional analysis of genes in cluster 2 outlined several interrelated canonical pathways involved in the transition through the G1/S stages of the cell cycle (Fig. 3b). The G1/S transition stage is a boundary between one active growth stage of cells (G1 phase) and DNA replication (S phase)^45^. Notably, key genes for G1/S transition such as *cyclin D* and *cdk4* ^46^, and DNA replication such as *pcna* ^47^, were observed in this cluster (Table 1). Response to DNA damage during S phase appears to derive from the up-regulation of interrelated molecular pathways such as those of BRCA1, GADD45, and p53 (Fig. 3b). Taken together, it is plausible that the moderate feed intake of *S. aurata* larvae during the morning^22^ (Supplementary Fig. S2) plays a role in determining the growth scope of the new day: cells check starting DNA quality and whether there are sufficient raw materials to replicate the DNA.

Genes in cluster 3 began to significantly increase their expression when the light was turned on, with maximal values 9–12 h later (Fig. 2c), coincident with the maximal feeding activity (Supplementary Fig. S2). The main canonical pathways in cluster 3 were related to cell cycle, including “G2/M DNA Damage Checkpoint Regulation” (Fig. 4a). During G2, cells continue to grow after DNA duplication in S, and during M phase mitosis occurs. *ccna2* (*cyclin A2*), *cdk1 (cyclin dependent kinase 1)*, and *ccnab1* (*cyclin B1)* (Table 2) were represented in this cluster. Cyclin A modulates the activity of CDK1 during the transition from G2 to M^48^ and is replaced by cyclin B to regulate the progression of M^49^, which may occur about 6-hours after maximal mitotic gene expression^50^. Interestingly, some genes in this cluster are involved in myogenesis, such as *mef2c, clock* and *myh* ^51–53^ (Supplementary Table S1). Moreover, our data indicate that during most of the feeding period and probably during early night, *S. aurata* larvae achieve a low level of ROS by the Nrf2-Mediated Oxidative Stress Response pathway (Fig. 4a) and resultant up-regulation of antioxidant defense proteins (e.g., glutathione S-transferase, heme oxygenase, Table 2). This shift in antioxidant defense strategy (i.e., limiting ROS production during overnight fasting to ROS-scavenging during feeding) likely results from the high energy cost of growth during G2 phase and muscle differentiation. Protein synthesis, an energetically expensive process, is likely to be enhanced during the second half of the feeding phase, as genes involved in the folding of nascent proteins (e.g., *hsp40*, *hsp70*, and *hsp90*) (Supplementary Table S1) were at their maximum of expression.

Analysis of cluster 4 genes revealed that molecular processes related to cell morphology, proliferation, growth, and development, were up-regulated over nearly the entire dark period (Figs 2c and 4b). Significant canonical pathways in this cluster included Neuregulin Signaling and Agrin Interactions at the Neuromuscular Junction (Fig. 4b). The concomitant up-regulation of these pathways^54,55^ suggests that neuromuscular junction formation in *S. aurata* larvae occurs during the second half of the dark period and likely extends to the next morning. Cluster 4 also appears to prepare non-differentiated cells for upcoming processes the next morning, such as proliferation, as myogenic progenitor cells (MPCs) are strongly stimulated by IGF-II^56^ present in cluster 4. This is also supported by G1/S Checkpoint Regulation and Estrogen-mediated S-phase Entry in cluster 4. In addition, we found *dio3* in this cluster (Supplementary Table S1), whose protein product might protect already differentiated tissues from T_3_-induced muscle differentiation^57^ or gate overall TR/RXR (cluster 1) effects to the next morning.

Cell cycle-regulated transcription in single cells is grouped into three main waves, coincident with the transition from G1 to S, G2 to M and M to G1^46^, but studies on whole organisms, including fishes, are scarce^58–60^. A striking outcome of this study is that 24-h variations in whole *S. aurata* larvae transcriptome resemble cell cycle progression (Supplementary Fig. S2). This observation revealed an unexpected synchrony in the whole larvae, as it is established that somatic cell cycles are usually not synchronous after early embryonic stages^58^. This situation at the whole larvae may be equivalent to local cell cycle synchrony of vertebrate adult tissues under high cell proliferation conditions (e.g., regeneration)^61^. As it has been proposed that cells with an interdivision time near to 24 h proliferate faster^62^, the 24-h synchrony in the cell cycle at the whole organism level may result in the high growth rate of fish larvae^26^ and led us to suggest a cell cycle-based model for daily growth of *S. aurata* larvae as detailed in Supplementary Fig. S2. As it is well established that clock genes play key roles in the circadian organization of metabolism^16^, cell cycle^63^, and myogenesis^64^, we next sought to establish whether core clock genes may drive the observed expression patterns.

### Putative role of clock genes in the daily organization of the larvae transcriptome

Virtually nothing is known about clock-driven transcription in the larvae of fishes other than zebrafish^2,3^. Our study provides evidence that the circadian clock plays a key role in daily transcriptome organization in *S. aurata* larvae. PER3 promotes stability and nuclear translocation of PER1/PER2^65^ and thus, it was not surprising that *per3* expression peak occurred (cluster 1) before *per1/per2* peak (cluster 2) (Figs 2, 5). Expression of *per3* (cluster 1) and *per2* (cluster 2) may be related to the negative control of PPARγ transcriptional activity and adipogenesis^66,67^ (*pparg* in cluster 2, Supplementary Table S1) during the last hours of the dark phase and the first hour of the next morning, suggesting that clock genes from one cluster may be functionally related to genes from the same or the next cluster.

The expression of several genes in cluster 2 was significantly correlated with *per1*/*per2* and *cry1*/*cry2* during the dark period (Fig. 5). The activation of *per* and *cry* genes in larvae occurred after 0–3 h of maximal *clock*/*bmal1* expression, in agreement with the activation of *per* and *cry* genes by CLOCK/BMAL1^13^. PER and CRY may act over several cluster 2 genes carrying E-box sequences^13^, and these primary clock-controlled genes will in turn regulate second order clock-controlled genes in cluster 2 and 3. The *per* and *cry* expression later decreased through the light phase, likely as a result of their feedback regulation assisted by casein kinase I (CK1)^68^. We observed *ck1* in cluster 4, attaining maximal expression three hours before the onset of light (Supplementary Table S1). Thus, a putative high availability of all CRY, PER, and CKI in the morning may facilitate their translocation to the nucleus and the interaction with CLOCK/BMAL1 heterodimers, thereby inhibiting transcription of *cry* and *per* genes.

The *bmal1* and c*lock* (cluster 3) up-regulation in *S. aurata* occurred during the light phase; *bmal1* rhythmicity is known to be driven by changes in its promoter occupancy by RORα (activator) and REV-ERBα (repressor)^69^. Both *rev-erba* and *bmal1* exhibited a similar expression pattern in *S. aurata* cluster 3, while *rora* was observed in cluster 2 (Fig. 5, Supplementary Table S1). These results indicate a finely tuned regulation of *clock*/*bmal1* expression during the day; after transcription of *rora* during the night, RORα proteins may mediate the up-regulation of *bmal1* during the day under negative regulation of REV-ERBα. This last transcription factor is also a key coordinator between circadian rhythms and metabolism, regulating metabolic pathways such as lipid and bile acid metabolism, adipogenesis, and gluconeogenesis^70^. Intriguingly, no core clock genes were identified in cluster 4, and thus this group of genes may be controlled indirectly by clock components of cluster 3 through their network output, especially transcription factors (6 and 13 in clusters 3 and 4, respectively). We are aware, however, that not all cycling genes described in our work must be directly or indirectly controlled by the clock but instead may be driven by other signals such as feeding in phase with the clock. We also acknowledge that functional interpretation of our correlation analysis may be hampered by the variable phase delay between the peak in protein accumulation and the peak in its respective transcript. Further experimental and computational approaches are needed to investigate this issue.

In general, main clock genes from clusters 1 and 2 (*per1, 2, and 3, cry1* and *2*) peaked at dawn and those from cluster 3 (*bmal1*, *clock*, and *nfil3*) at dusk. These temporal patterns are in line with those observed before in *S. aurata* brain^20^ and whole larvae^22^, and in zebrafish muscle^71^ and whole larvae^3^. However, zebrafish *cry1* and *cry2* were observed in antiphase^3,71^, while these genes were in phase in *S. aurata*. Additionally, in whole zebrafish larvae, Li *et al*. found that *per2* rapidly increased its expression after light exposure (peak after 3 h)^2^, whereas we found that *per2* was transcribed through all night in *S. aurata* to peak in the morning (also after 3 h of light), indicating differences among fish species in the responsiveness to light of *per2*. In addition, it is well known that chromatin remodeling is crucial for the clock function^72^. We observed histone acetyltransferases in cluster 2 (KAT2A and KAT7) and cluster 4 (KAT2B and KAT5) (Supplementary Table S1), which are transcriptional activators. Subsequently, genes coding for transcriptional repressors such as histone deacetylases (HDAC3) and methyltransferases (EHMT2, SETDB1-B) were found in cluster 3 (Supplementary Table S1). Hence, at least two waves of expression of chromatin remodeling enzymes may mediate clock-driven circadian transcription in *S. aurata* larvae.

### Paving the way to indicators of circadian synchrony discovery

A plethora of studies during the last decade and recent research have resulted in an extended list of useful biomarkers for nutritional status, growth, metabolism and health in various farmed fish species (e.g., www.nutrigroup-iats.org/arraina-biomarkers). However, compared to juveniles, relatively few biomarkers are available for larval stages. We suggest that genes showing significant correlated synchrony with core clock genes may be suitable candidate biomarkers of circadian physiology, and those retained after filtration by pathways overlapping have the potential to be highly informative, as they may reflect the concerted action of several pathways leading to a particular phenotype. Taking this into account, we identified different candidate biomarkers of cell cycle progression, neuromuscular development and growth, and protection against oxidative stress (Supplementary Table S3) that offer many evaluation possibilities as briefly explained below.

It is well established that in animals, growth, health, and well-being depend on the synchronization of endogenous biological rhythms and environmental clues. Conventionally, circadian rhythm disturbance assessment requires repeated measurements of biomarkers through the 24 h cycle. Our study provides extensive information on daily variations of candidate genes (Supplementary Table S3) to follow this approach in *S. aurata* larvae. However, when several sampling points are not practical under routine aquaculture operations, we suggest to alternatively use a single sampling point to assess the expression of two subsets of selected genes from antiphase clusters (e.g., cluster 1 and 3), to find ratio indicators of synchrony. The use of these algorithms has the additional advantage of decreased variability of synchrony indicators among individuals or groups.

Also, we suggest that ratios resulting from the combined analysis of candidate biomarker genes at two different times may be informative about a given process scope. For example, it may be worthwhile to explore whether some combination of indicators of cell cycle progression (e.g., G1/S specific cyclin D1 at ZT3: G2/M specific cyclin B1, 2, or 3 at ZT12, Supplementary Table S3) are suitable predictors of larval growth scope. In addition, the occurrence of a higher number of MPCs resulting from proliferation during early development in teleosts may be advantageous for future growth due to increased fiber number (i.e., hyperplasia)^53,73^ as occurred in salmon^74^ and cod^75^. For this reason, we suggest the combined evaluation of *pcna* and other indicators of proliferation at ZT3 and *myh*-*striated muscle* and others indicators of differentiation at ZT 12 (Supplementary Table S3), as predictors of musculature development in *S. aurata* larvae. Likewise, the combined assessment of genes involved in antioxidant responses would lead to the selection of larvae batches with higher metabolic capacity. For instance, voluntary energy intake in fish was suggested to be limited by oxidative metabolism capacity, likely due to the detrimental effects of ROS^76^. Up-regulation of genes involved in the maintenance of the oxidative status was observed in Senegalese sole^77^ and cod^78^ larvae under high growth rate conditions. We suggest the combined evaluation of *upc1/2/3* at ZT0 and *gst*, *hmox*, and several *hsp* at ZT12 (Supplementary Table S3) as putative indicators of the scope of oxidative metabolism of larvae.

In summary, this study demonstrates that the molecular circadian clock and metabolic rhythms are highly synchronized in early life stages, likely allowing *S. aurata* larvae to grow at a high rate. Our results also offer the possibility to identify early predictors of fish performance taking into account the changes in daily physiology. Given that *S. aurata* is a commercially relevant fish species for which genomic resources are increasingly available, this study opens new opportunities to unravel the complexity of daily gene regulation, with implications for fundamental and applied research.

## Methods

### Experimental setup

*S. aurata* larvae were reared at ICMAN-CSIC animal experimentation facilities (REGA number ES110280000311) in three circular 250-L tanks under constant temperature (19 °C) and salinity (34‰) and a 12 h light: 12 h darkness cycle. The light was switched on at zeitgeber time (ZT) 0 (09:00 h local time) and off at ZT 12 (21:00 h local time). The larvae were fed *ad libitum* with rotifers *(Brachionus rotundiformis* Bs-strain and *B. plicatilis* S-1-strain) supplied at a density of 10 rotifers/mL and enriched with the microalgae *Nannocholopsis gaditana* from day 4 post-hatching (dph) and subsequently with *Artemia sp*. nauplii from 18 dph until the end of the experiment^79^. Larvae were sampled at 30 dph (middle of the larval stage) during a 24 h cycle. Six individuals (two per tank) were taken every 3 hours (00:00, 03:00, 06:00, 09:00, 12:00, 15:00, 18:00, 21:00 and 24:00 h ZT) and preserved in RNA*later* (Ambion). All experimental procedures complied with the Guidelines of the European Union Council (2010/63/EU) for the use and experimentation of laboratory animals and were reviewed and approved by the Spanish National Research Council (CSIC) bioethical committee.

### RNA extraction for microarray analysis

Total RNA was extracted from whole larvae using an Ultra-Turrax T8 (IKA^®^-Werke) and the NucleoSpin^®^ RNA II kit (Macherey-Nagel), including the on-column RNase-free DNase digestion included with the kit. RNA quantity was measured spectrophotometrically at 260 nm with a BioPhotometer Plus (Eppendorf) to a yield of 3 to 15 µg. RNA quality was checked in a Bioanalyzer 2100 and with the RNA 6000 Nano kit (Agilent Technologies). RIN (RNA integrity number) measurements ranged between 8.4 and 10, indicative of clean and intact RNA.

### Transcriptome database construction and annotation

Blast comparisons were conducted between the assembled sequences of the Nutrigroup-IATS nucleotide database^27^ and those from pyrosequencing of 454 libraries of larval origin^28^ to combine both into a unique database. In the case of overlapping sequences with shared homology, the longest was retained. Larval sequences with no equivalent in the previous Nutrigroup-IATS database were annotated by searching sequence homologies against 24 different nucleotide and protein databases previously reported^27^, and subjecting them to the same algorithm of frame shift detection to correct potential 454 sequencing errors at homopolymer regions^80^. The updated *S. aurata* nucleotide database was hosted at www.nutrigroup-iats.org/seabreamdb, and it contains 3,388 larval annotated sequences (e-value < 1e-5) of a total of 20,565 non-redundant sequences encoding for 14,546 unique transcripts.

### Microarray construction, hybridization and data analysis

The updated *S. aurata* database was the basis for a custom high-density oligo-microarray (8 × 15 K) (sea_bream_nutrigroup_array v.3), that was designed and printed using the eArray web tool (Agilent). The array comprised 60-oligomer probes for 13,939 different *S. aurata* genes and was used herein for circadian transcriptomic profiling of 30-day-old larvae. The design of the array was stored in the NCBI Gene Expression Omnibus (GEO) database under accession identifier GPL19579. Total RNA (150 ng) from individual fish (n = 6 for each group) were labeled with cyanine 3-CTP (Low Input Quick Amp Labelling Kit, Agilent), and 600 ng of each labeled cRNA were hybridized to microarray slides that were analyzed with an Agilent G2565C Microarray Scanner according to the manufacturer´s protocol. Data were extracted using the Agilent Feature Extraction Software 11.5.1.1 and deposited in the GEO database under accession identifier GSE64481. Microarray data analysis was performed with Genespring GX 13.0 software (Agilent). After quality control assessment, raw data (median intensity of each spot) were extracted and corrected for background with the Agilent Feature Extraction plug-in, and the intensity values were normalized using the 75^th^ percentile shift. Functional pathway analysis was performed with IPA software (www.ingenuity.com). For each gene, the Uniprot accession of the annotation equivalent for one of the three higher vertebrates model species in IPA (human, rat or mouse) was assigned.

### Real-time qPCR validation of microarray results

Nine differentially expressed genes covering a wide range of low and high hybridization intensity levels and fold-change variations were chosen for real-time qPCR analysis: *cry1* (GenBank Accession Number JQ965014), *clock* (JQ965015), *bmal1* (JQ965013), *ucp3* (EU555336), *pcna* (KF857335), *catalase* (JQ308823), *26 S proteasome non-ATPase regulatory subunit 4* (*psmd4*; KM522789), *cyp7a1* (KX122017) and *surfeit locus protein* (*surf*; KC217650). Validation was performed on the same individual samples used for microarray analyses (6 individuals for each of the nine sampling points) by real-time qPCR, using PerfeCTa™ SYBR® Green FastMix™ (Quanta BioSciences) in a Mastercycler ep *gradient* S Realplex^2^ (Eppendorf). Primer design, reverse transcription, qPCR optimization, and qPCR reactions were performed as previously detailed^33^. Specificity of reaction was verified by melting curve analyses and electrophoresis. Data were normalized to β-actin using the ΔΔCt method^81^ and fold-changes were referred to fish at 24 h zeitgeber time.

### Statistical analysis

Microarray results from the nine experimental groups were analyzed by one-way ANOVA (corrected P-value < 0.05, Benjamini-Hochberg), PCA, k-means clustering and correlation analysis with similar entities by means of the Genespring GX 13.0 software (Agilent). PCA eigenvalues were determined by means of Genesis software (release 1.7.7). Optimal number of clusters was determined on the within-group sum of squares calculated by means of the k-means script in R. Ingenuity Pathway analysis used Fisher’s exact test was used to calculate a P-value reflecting the probability that the association between the set of molecules and a given pathway was due only to chance. Threshold of the P-value for association was set to 0.01. In overlapping pathway analysis, settings were selected to guarantee a minimum of 4 common genes between different canonical pathways.

### Data availability

The updated *S. aurata* nucleotide database is available at www.nutrigroup-iats.org/seabreamdb. The design of the array was stored in the NCBI Gene Expression Omnibus (GEO) database under accession identifier GPL19579 and data obtained were deposited in the GEO database under accession identifier GSE64481. All other data generated or analyzed during this study are included in this published article and its Supplementary Information files.

## Electronic supplementary material

Supplementary Information
