# Supplementary material for: The circadian transcriptome of marine fish (Sparus aurata) larvae reveals highly synchronized biological processes at the whole organism level
Source: Sci Rep. 2017 Oct 11;7:12943. doi: 10.1038/s41598-017-13514-w (PMC5636797; doi:10.1038/s41598-017-13514-w)
Supplement: Supplementary file 1 — Supplementary Information [file 41598_2017_13514_MOESM1_ESM.pdf]

The circadian transcriptome of marine fish (*Sparus aurata*) larvae reveals highly synchronized biological processes at the whole organism level

M. Yúfera, E. Perera, J.A. Mata-Sotres, J. Calduch-Giner, G. Martínez-Rodríguez, J. Pérez-Sánchez

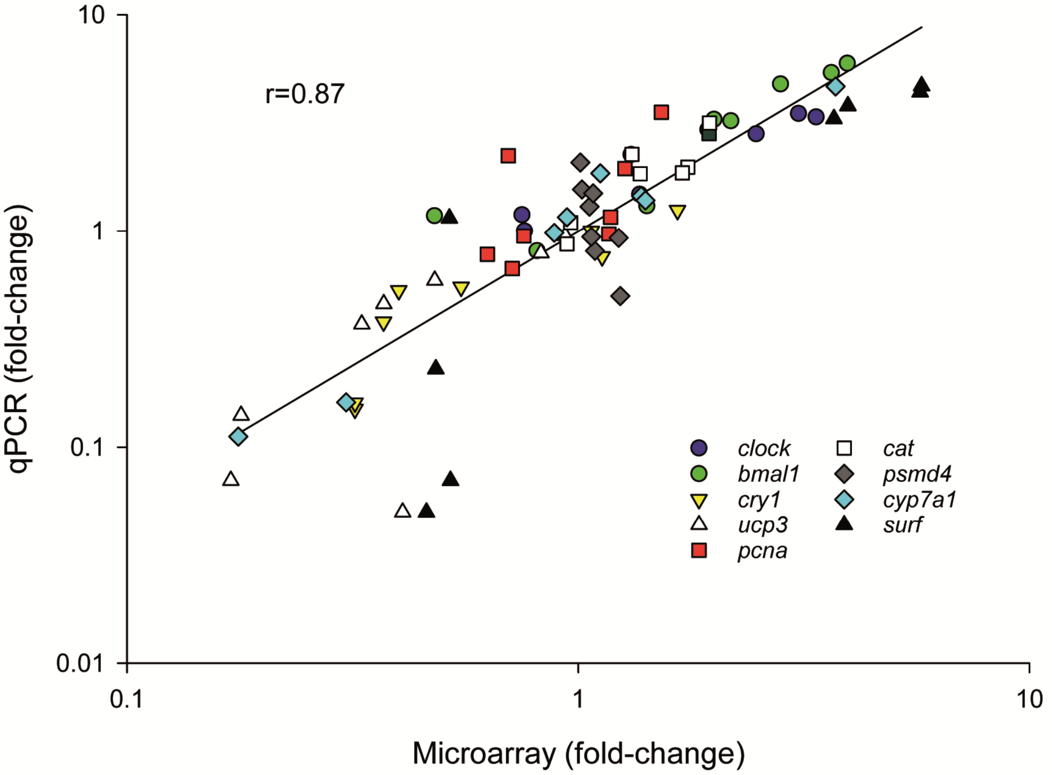

Primers pairs used for qPCR

Gene	Forward primer (5' → 3')	Reverse primer (5' → 3')	Amplicon size (pb)	E	R^2
clock	TGCGTTTGATGACCAGGTGT	GAATTCTTCATTGGGCTCCTCC	100	0.95	0.98
bmal1	CAGAGCCTGTTTGACTACCTTC	TAGCGTCAATGAGCCTCTCT	100	0.90	0.99
cry1	TCCTTCTTCCAGCAGTTCTTCC	GGCGTTCCAGGGATCATAAATAT	141	0.99	0.99
ucp3	AGGTGCGACTGGCTGACG	TTCGGCATACAACCTCTCCAAAG	108	0.99	0.99
pcna	CGTATCTGCCGTGACCTGT	AGAACTTGACTCCGTCCTTGG	72	0.99	0.99
cat	TGGTCGAGAACTTGAAGGCTGTC	AGGACGCAGAAATGGCAGAGG	147	0.94	0.97
psmd4	CATCCACACCTGCTCTACCAGACTTCA	CGTAGGCGATCTGTTCATCCTCTGTCAT	59	0.94	0.99
cyp7a1	CCCTGCTATTAAAGTCCCACCTCT	ATCGTAGGTAGGCTGGAGGATTC	69	0.84	0.99
surf	AGATGGAAGGTGAAGTGGAGGTGGTC	GCGTTGCTCTGTCTGCCGAACT	52	0.96	0.98
actb	TCTTCCAGCCATCCTTCCTCG	TGTTGGCATACAGGTCCTTACGG	108	1	0.99

clock: circadian locomotor output cycles kaput; *bmal1*: aryl hydrocarbon receptor nuclear translocator-like protein 1; *cry1*: cryptochrome 1; *ucp3*: uncoupling protein 3; *pcna*: proliferating cell nuclear antigen; *cat*: catalase; *psmd4*: 26S proteasome non-ATPase regulatory subunit 4; *cyp7a1*: cholesterol 7-alpha-monooxygenase; *surf*: surfeit locus protein; *actb*: beta-actin

Supplementary Figure S1. qPCR validation of microarray results. Correlation (r=0.87) plot of fold-change values for selected genes analyzed by microarray (X-axis) and qPCR (Y-axis). Fold-changes referred to the 24 h sampling point. Primers used for results validation by qPCR are shown in the bottom table, with amplicon sizes (bp), reaction efficiencies (E) and coefficients of determination (R^2). Primer design, reverse transcription, and qPCR optimization were performed as before[33]. qPCR reactions were performed with 10 ng of cDNA, 200 nM of each primer, an initial denaturation step for 10 min at 95°C; 40 cycles of denaturing for 15 s at 95°C, annealing and extension for 45 s at 60°C; and a final melting curve from 60°C to 95°C for 20 min. Data were normalized to β-actin using the ΔΔCt method[81].

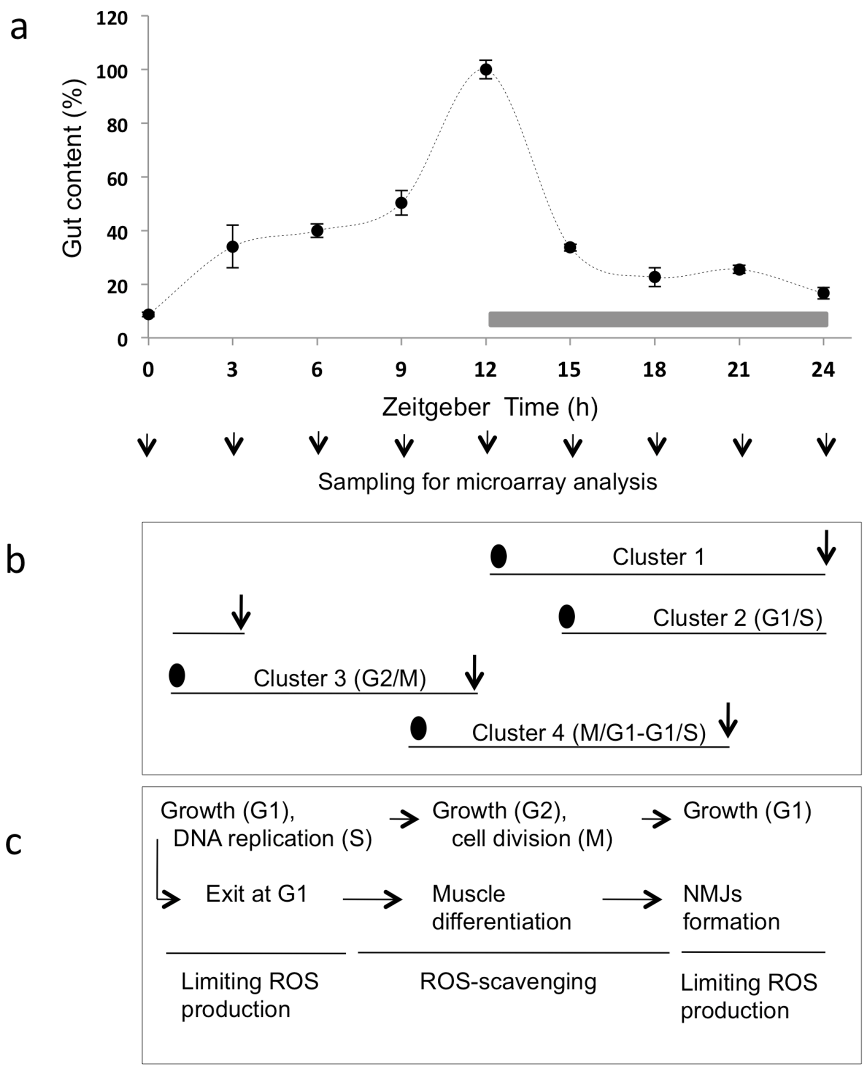

Supplementary Figure S2. Daily feed intake and circadian regulation of growth and metabolism in *S. aurata* larvae. a) Gut fullness (*Artemia*) percentage of gilthead seabream larvae at 30 dph under culture conditions (mean ± SEM, n=10). Grey bar indicates dark phase. Modified from a previous work[22]. Arrows indicate sampling time for microarray analysis. **b)** Consecutive up-regulation of clusters 1 to 4 gating whole-organism cell cycle. Black ovals mean activation. Arrows indicate maximal gene expression. **c)** Inferred phases of *S. aurata* larvae growth (above the line) and oxidative metabolism (under the line). NMJs: neuromuscular junctions. *S. aurata* larvae daily growth may be separated into three periods: *i*) A first period during the morning when cells grow without replicating DNA (equivalent to G1) and decide whether to replicate the DNA (equivalent to S), likely in the afternoon. During this period, cells that exit G1 begin to differentiate. *ii*) A second period comprising the second half of the light phase (maximal feeding activity), in which larvae cells keep growing (equivalent to G2), and the first half of the dark phase when cells divide (equivalent M phase). In this period, terminal muscle cell differentiation occurs. *iii*) A third period, during the second half of the night, when cell differentiation is blocked, neuromuscular junction formation occurs, and cell proliferation is activated again to start a new cycle.

The circadian transcriptome of marine fish (*Sparus aurata*) larvae reveals highly synchronized biological processes at the whole organism level

M. Yúfera, E. Perera, J.A. Mata-Sotres, J. Calduch-Giner, G. Martínez-Rodríguez, J. Pérez-Sánchez

Supplementary Table S1. List of differentially expressed genes (P < 0.05, one-way ANOVA with corrected P value, tukey´s HSD post hoc test, Benjamin-Hocherg multiple testing correction) with fold-change values referred to fish at 24 h zeitgeber time.

Gene Name	Fold-Change								Cluster
	00/24	03/24	06/24	09/24	12/24	15/24	18/21	21/24	
2-epi-5-epi-valiolone synthase	1.29	1.25	-1.21	-1.99	-5.19	-5.80	-4.10	-1.41	1
2-epi-5-epi-valiolone synthase-like	1.25	1.24	-1.37	-2.42	-6.67	-6.75	-3.57	-1.32	1
60S acidic ribosomal protein P0-like	1.23	-2.31	-7.22	-13.87	-7.57	-2.92	1.82	3.85	1
60S ribosomal protein L13a	-1.45	-1.54	-2.50	-2.51	-3.33	-2.39	-1.58	-1.31	1
ADP-ribosylation factor-like protein 5B	-1.26	-1.89	-3.31	-6.53	-4.89	-2.76	-2.52	1.11	1
ADP-ribosylation factor-like protein 5C	-1.12	-1.73	-3.11	-6.30	-4.38	-2.58	-2.30	1.13	1
Alpha-crystallin B chain	-1.18	-1.75	-5.00	-14.23	-27.11	-21.03	-4.20	-1.54	1
Alpha-N-acetylgalactosaminide alpha-2.6-sialyltransferase 1	-1.12	-1.56	-3.81	-3.48	-4.52	-3.51	-1.20	1.18	1
Ammonium transporter Rh type B	1.17	-1.15	-2.39	-3.28	-3.76	-2.47	-1.02	1.44	1
Ammonium transporter Rh type C 2	1.39	1.12	-1.62	-3.10	-3.59	-3.95	-2.04	-1.04	1
Angiopoietin-related protein 7	1.05	-1.48	-2.06	-2.35	-2.73	-1.95	-1.47	-1.02	1
Angiotensin-converting enzyme 2	1.25	-1.03	-1.49	-1.81	-2.08	-1.60	-1.33	1.17	1
Ankyrin repeat domain-containing protein 33B	1.04	-1.36	-4.04	-8.38	-3.27	-2.35	-2.49	1.19	1
Ankyrin repeat domain-containing protein 9	-1.05	-1.40	-1.24	-2.46	-3.87	-3.38	-2.47	-1.26	1
Ankyrin repeat domain-containing protein 9-like	-1.07	-1.54	-1.26	-2.46	-3.82	-3.71	-2.78	-1.25	1
Aquaporin-10	1.33	-1.19	-3.18	-6.36	-6.99	-5.00	-1.80	1.44	1
Arginase. non-hepatic 2	-1.13	-1.07	-1.97	-3.29	-4.18	-7.46	-5.29	-2.23	1
Arrestin-C	-1.06	-1.32	-1.54	-2.73	-4.15	-2.63	-3.98	-1.13	1
Aspartoacylase	-1.01	-1.33	-1.98	-2.56	-2.37	-1.74	-1.34	1.18	1
ATP-binding cassette sub-family G member 2	1.26	1.07	-1.46	-1.89	-2.62	-1.58	-1.04	1.17	1
Beta.beta-carotene 15.15'-monooxygenase	1.37	1.03	-2.62	-6.02	-6.53	-4.45	-3.49	1.34	1
Carboxypeptidase O	1.41	-1.04	-1.44	-1.76	-2.78	-2.17	-1.12	1.54	1
Caspase-7	1.59	1.22	-1.50	-3.51	-9.12	-4.33	-3.69	-1.21	1
CC chemokine CK3	-1.28	-1.60	-2.87	-7.17	-5.86	-3.59	-1.56	-3.22	1
Cholesterol 7-alpha-monooxygenase	1.38	-1.13	-3.27	-5.67	-3.72	1.12	-1.06	1.41	1
Circularly permutated Ras protein 1	1.20	-1.08	-1.84	-3.04	-3.96	-3.99	-2.21	-1.33	1
Class E basic helix-loop-helix protein 40	-1.02	1.20	-1.07	-6.26	-21.62	-30.69	-6.83	-1.81	1
Collagen alpha-3(VI) chain-like	-1.10	-1.58	-1.89	-2.53	-3.43	-2.10	-1.99	-1.10	1
Cyclic nucleotide-gated cation channel alpha-3	1.18	1.09	-2.28	-5.29	-3.22	-1.48	-1.33	1.58	1
Cyclic nucleotide-gated channel cone photoreceptor subunit alpha-like	1.11	-1.28	-3.50	-5.67	-3.41	-1.39	-1.96	1.67	1
Cyclic nucleotide-gated olfactory channel	1.07	1.38	-1.54	-2.29	-1.66	-2.30	-1.05	1.21	1
Cytidine deaminase	-1.28	-1.43	-2.01	-4.69	-3.27	-2.65	-1.82	-1.17	1
Cytochrome P450 1A1	-1.07	1.00	-1.71	-3.30	-3.26	-3.59	-2.11	-1.04	1
Cytoskeletal protein Sojo-like	-1.14	-1.12	-1.83	-2.80	-3.29	-2.32	-1.84	-1.18	1
Cytosolic 5'-nucleotidase III-like protein	-1.51	1.13	-1.84	-2.41	-4.18	-2.17	-1.13	-1.02	1
Digestive cysteine proteinase 3	2.09	1.50	-1.02	-2.11	-2.13	-1.14	1.58	3.89	1
E3 ubiquitin-protein ligase MYLIP-A	1.20	1.15	-1.11	-1.91	-2.38	-3.30	-3.21	-1.15	1
Endonuclease 8-like 1	-1.15	1.32	-1.47	-3.17	-4.30	-4.81	-4.86	-2.55	1
Epidermal growth factor receptor kinase substrate 8-like protein 1-like isoform X2	-1.08	-1.38	-2.83	-3.20	-2.86	-2.06	-1.74	-1.26	1

Gene Name	Fold-Change								Cluster
	00/24	03/24	06/24	09/24	12/24	15/24	18/21	21/24	
Eukaryotic translation initiation factor 4E-binding protein 3-like	-1.20	-1.50	-2.38	-3.33	-3.45	-2.50	-1.96	-1.25	1
Excitatory amino acid transporter 5	-1.03	-1.06	-1.65	-5.14	-8.14	-6.11	-1.95	1.95	1
Gap junction Cx32.2 protein	1.13	-1.06	-1.76	-3.23	-4.61	-4.16	-2.00	-1.17	1
Glutaminase kidney isoform. mitochondrial	1.09	1.02	-2.50	-3.05	-4.00	-3.88	-1.49	-1.10	1
Glycogenin-1	1.00	-1.16	-1.19	-1.71	-2.30	-1.35	-1.40	1.72	1
Growth hormone receptor type II	-1.57	-2.05	-3.98	-4.36	-3.91	-2.13	-1.93	-1.09	1
Guanylyl cyclase-activating protein 2	1.28	1.04	-1.75	-3.79	-1.80	-1.55	-1.65	1.29	1
Hyaluronan synthase 1	1.06	-1.08	-2.70	-4.00	-14.50	-5.69	-1.86	-1.29	1
Hypothetical protein LOC100707031	1.25	-1.04	-1.32	-2.00	-2.62	-2.14	-2.16	1.34	1
Hypoxia-inducible factor 3-alpha	-1.32	-1.57	-1.95	-3.42	-2.74	-2.35	-1.85	-1.43	1
Ileal sodium/bile acid cotransporter	-1.00	-1.30	-3.08	-3.58	-5.88	-3.56	-1.99	-1.43	1
Inositol 1.4.5-triphosphate receptor-interacting protein	-1.31	-1.14	-1.80	-1.97	-2.63	-2.98	-1.68	-1.21	1
Interferon-related developmental regulator 2	1.34	-1.27	-1.44	-2.31	-2.41	-1.81	-1.03	1.39	1
Interleukin-12 subunit beta	1.14	-1.09	-1.41	-2.18	-2.56	-1.43	-1.57	1.06	1
Intestinal-type alkaline phosphatase	1.50	1.09	-2.08	-3.66	-5.08	-1.93	-1.16	1.58	1
Large neutral amino acids transporter small subunit 4	1.05	-1.11	-2.08	-2.13	-2.85	-2.05	-1.37	-1.06	1
Leucine-rich repeat. immunoglobulin-like domain and transmembrane domain-containing protein 1	1.00	1.23	-1.93	-6.94	-7.28	-5.22	-6.11	-2.70	1
Leucine-rich repeat. immunoglobulin-like domain and transmembrane domain-containing protein 3	1.19	1.56	-1.97	-4.26	-4.82	-2.84	-3.60	-2.58	1
Leucine-rich repeat-containing protein 17	-1.04	1.01	-1.69	-2.88	-3.61	-4.64	-2.91	-1.47	1
Leucine-rich repeat-containing protein 30	-1.04	-1.47	-1.66	-2.52	-3.81	-3.49	-2.62	-1.51	1
LIM domain only protein 7-like	1.01	-1.01	-1.63	-2.16	-2.20	-1.85	-1.09	-1.02	1
Low affinity cationic amino acid transporter 2	1.26	1.06	-1.58	-2.01	-2.81	-2.01	-1.37	1.11	1
L-serine dehydratase/L-threonine deaminase	-1.13	1.45	-1.10	-3.19	-13.33	-57.63	-22.01	-9.44	1
L-threonine 3-dehydrogenase. mitochondrial	-1.12	-1.74	-3.90	-6.68	-7.34	-8.02	-3.45	-1.47	1
Lysyl oxidase homolog 2	-1.06	-1.50	-1.92	-2.36	-3.37	-2.81	-1.91	-1.11	1
Macrophage-expressed gene 1 protein	1.16	-1.36	-1.65	-2.67	-2.83	-1.48	1.06	1.29	1
Membrane progestin receptor gamma-B	1.16	-1.17	-1.89	-3.18	-3.33	-2.81	-1.26	1.18	1
Mitochondrial uncoupling protein 1 (UCP1)	-1.22	-1.04	-1.76	-2.47	-3.05	-2.74	-1.66	-1.07	1
Mitochondrial uncoupling protein 2	1.13	-1.21	-1.58	-3.16	-2.96	-2.50	-2.40	-1.31	1
Mitochondrial uncoupling protein 2 (UCP2)	-1.21	-1.42	-1.76	-5.20	-12.97	-14.69	-13.67	-3.47	1
Mitochondrial uncoupling protein 3 (UCP3)	-1.07	-2.08	-3.02	-5.59	-5.89	-2.70	-2.45	-1.21	1
Monocarboxylate transporter 12-B	-1.23	-1.74	-4.00	-6.66	-7.04	-3.44	-1.88	-1.05	1
Mucolipin-2	1.00	-1.36	-2.77	-2.98	-3.84	-2.31	-1.95	-1.06	1
Na(+)/H(+) exchange regulatory cofactor NHE-RF1	1.23	1.19	-1.40	-2.12	-2.16	-2.97	-1.75	-1.05	1
Na(+)/H(+) exchange regulatory cofactor NHE-RF4	-1.03	-1.04	-1.76	-2.34	-2.62	-2.21	-1.38	1.01	1
NADPH--cytochrome P450 reductase	-1.34	-1.48	-1.74	-2.41	-3.29	-3.01	-2.67	-1.22	1
Nef-associated protein 1	-1.09	-1.26	-2.13	-3.50	-2.44	-2.16	-1.32	-1.20	1
Neurocalcin-delta B	-1.10	-2.25	-3.39	-4.01	-4.89	-2.78	-2.63	1.26	1
Neurofilament heavy polypeptide-like isoform X2	-1.19	-1.04	-1.18	-1.74	-2.67	-2.03	-1.42	1.26	1

Gene Name	Fold-Change								Cluster
	00/24	03/24	06/24	09/24	12/24	15/24	18/21	21/24	
Nuclear receptor subfamily 1 group D member 2	1.02	-1.13	-3.83	-12.74	-8.78	-3.64	-1.45	-1.38	1
Nuclear receptor subfamily 4 group A member 1	1.09	-1.79	-2.25	-4.03	-4.38	-1.80	-1.42	1.42	1
PAP-associated domain-containing protein 5	1.13	1.02	-1.39	-2.01	-2.23	-2.09	-1.30	1.06	1
Peptidyl-prolyl cis-trans isomerase FKBP5	-1.46	-1.88	-1.37	-3.62	-5.70	-5.17	-2.75	1.93	1
Period circadian protein homolog 3	-1.17	-1.49	-4.83	-6.52	-6.66	-5.90	-3.31	-1.33	1
Phosducin	1.17	1.44	-1.15	-2.21	-2.60	-2.58	-2.64	-1.09	1
Phosphodiesterase 6H. cGMP-specific. cone. gamma	1.21	1.64	1.25	-2.14	-6.12	-16.92	-15.49	-1.83	1
Phosphoenolpyruvate carboxykinase. cytosolic [GTP]	-1.29	-2.82	-9.53	-13.30	-11.84	-16.69	-12.21	-1.80	1
Polyserase-2-like	1.17	1.74	-1.32	-1.06	-1.47	-5.54	-1.31	1.27	1
Probable N-acetyltransferase 8B	1.44	-1.71	-5.43	-12.10	-6.30	-2.27	1.26	1.98	1
Proline dehydrogenase 1. mitochondrial	-1.46	-1.87	-4.37	-9.84	-17.53	-28.00	-5.87	-1.73	1
Prostaglandin E synthase	-1.08	-1.17	-1.69	-2.91	-3.20	-2.62	-1.97	-1.31	1
Protein EFR3 homolog B	1.43	1.38	-1.43	-2.69	-2.56	-3.26	-2.15	1.13	1
Protein FAM176A	1.02	-1.23	-2.13	-3.74	-3.68	-3.68	-2.69	-1.11	1
Protein FAM46A	-1.02	1.01	-1.40	-2.33	-2.79	-2.55	-1.58	-1.40	1
Protein phosphatase 1 regulatory subunit 3C	1.11	1.02	1.08	-2.61	-2.76	-2.05	-1.18	1.27	1
Protein phosphatase 1K. mitochondrial	-1.24	-1.25	-2.14	-2.55	-2.61	-2.39	-1.93	-1.25	1
Protein Tob1	-1.13	-1.66	-2.55	-3.84	-3.95	-2.28	-1.66	-1.18	1
Protein tyrosine phosphatase domain-containing protein 1	1.02	-1.02	-2.15	-2.29	-2.14	-2.53	-1.57	-1.01	1
Purpurin	1.16	1.21	-1.46	-2.26	-1.84	-2.89	-4.07	-1.06	1
Purpurin-like	1.25	1.30	-1.57	-2.89	-2.46	-3.85	-7.02	-1.35	1
Retinol-binding protein 2	1.11	1.06	-1.24	-1.94	-3.01	-4.04	-2.21	-1.33	1
Rhotekin	1.26	1.59	1.09	-1.72	-3.06	-4.36	-2.76	-1.44	1
S-adenosylmethionine decarboxylase proenzyme	-1.18	-1.58	-2.43	-3.39	-3.88	-4.09	-2.20	-1.28	1
Sarcospan	1.31	-1.40	-1.87	-1.97	-2.34	-2.29	-1.53	-1.05	1
Serine/threonine-protein kinase NIM1-like	1.06	-1.35	-1.77	-3.85	-6.24	-4.50	-2.98	1.08	1
SH2 domain-containing protein 3C	-1.12	-1.37	-2.03	-2.07	-5.19	-2.16	-1.86	-1.39	1
Sodium- and chloride-dependent GABA transporter ine	1.37	1.06	-1.25	-1.83	-3.05	-2.80	-1.89	-1.00	1
Sodium/glucose cotransporter 1	1.36	1.31	-1.36	-1.82	-1.95	-1.76	1.03	1.47	1
Sodium/nucleoside cotransporter 2	1.26	1.03	-1.46	-1.82	-2.76	-1.37	-1.02	1.37	1
Solute carrier family 12 member 7	1.00	-1.30	-1.78	-2.65	-2.46	-1.66	-1.40	1.17	1
Solute carrier family 13 member 2	1.08	-1.04	-1.58	-2.33	-2.27	-1.82	-1.42	1.01	1
Solute carrier family 15 member 1	1.32	-1.67	-4.68	-9.80	-6.35	-2.10	1.23	1.92	1
Solute carrier family 25 member 33	-1.08	-1.29	-3.10	-3.45	-3.53	-4.21	-2.17	-1.63	1
Solute carrier family 26 member 6	1.29	1.01	-1.42	-2.35	-2.87	-2.30	-1.92	1.05	1
Sorting nexin-16	1.04	-1.09	-2.61	-2.93	-3.78	-2.68	-2.17	-1.25	1
Sushi domain-containing protein 2	1.17	-1.19	-1.53	-1.99	-2.35	-2.01	-1.24	1.194	1
Telethonin	-1.15	1.05	-1.59	-2.82	-3.27	-5.35	-3.22	-1.75	1
Thyrotroph embryonic factor	-1.37	-1.90	-5.29	-8.09	-7.38	-3.66	-1.85	1.30	1
Transcription factor CP2-like protein 1	-1.03	-1.01	-1.58	-2.43	-4.40	-5.16	-3.59	-1.13	1
Transcription factor VBP	-1.02	-1.21	-2.08	-3.62	-6.21	-7.04	-3.31	-1.41	1
Transmembrane protein 179B-like	1.12	1.03	-1.18	-2.22	-3.38	-3.24	-2.52	-1.03	1

Gene Name	Fold-Change								Cluster
	00/24	03/24	06/24	09/24	12/24	15/24	18/21	21/24	
Tryptophan 2.3-dioxygenase A	-1.35	-1.86	-5.56	-17.98	-14.79	-2.22	-1.00	1.30	1
TSC22 domain family protein 3	-1.19	-1.27	-1.23	-2.58	-4.96	-3.29	-2.22	-1.19	1
Tumor protein D54-like	1.10	-1.41	-2.33	-5.17	-4.67	-2.26	-1.74	1.41	1
Ubiquitin carboxyl-terminal hydrolase 2	1.35	-1.01	-2.07	-4.33	-6.19	-7.53	-2.48	1.02	1
UDP-glucuronosyltransferase 2B17	1.11	-1.08	-1.52	-2.45	-3.09	-3.33	-2.10	-1.19	1
Uncharacterized protein C14orf118 homolog	-1.57	-1.90	-4.82	-9.82	-9.10	-5.41	-3.01	-1.25	1
Uncharacterized protein C1orf51	-1.14	-1.88	-3.74	-5.24	-3.79	-3.22	-1.32	2.48	1
Uncharacterized protein KIAA1737	-1.20	-2.96	-3.41	-7.04	-10.97	-4.65	-2.83	3.55	1
Uridine phosphorylase 2	-1.26	-1.40	-3.23	-7.01	-12.42	-7.03	-3.09	-2.15	1
Xaa-Pro aminopeptidase 2	-1.08	-1.08	-1.40	-2.13	-2.99	-2.94	-2.45	-1.27	1
XK-related protein 9	1.12	1.05	-1.54	-2.55	-3.95	-5.34	-2.93	-1.11	1
Zinc finger protein 40	-1.33	-1.33	-1.95	-2.96	-4.43	-2.82	-2.01	1.02	1
17-beta-hydroxysteroid dehydrogenase 14	1.17	1.19	1.13	-1.06	-1.14	-1.23	-1.20	-1.03	2
1-acyl-sn-glycerol-3-phosphate acyltransferase delta	1.11	-1.02	-1.03	-1.14	-1.32	-1.24	-1.08	1.08	2
23 kDa integral membrane protein-like	1.09	1.29	-1.14	-1.31	-1.17	-1.61	1.06	1.09	2
28S ribosomal protein S11. mitochondrial	-1.11	-1.15	-1.15	-1.26	-1.49	-1.40	-1.19	-1.23	2
28S ribosomal protein S2. mitochondrial	1.04	-1.07	-1.29	-1.44	-1.45	-1.46	-1.23	-1.15	2
2-acylglycerol O-acyltransferase 1	1.07	1.20	-1.27	-1.30	-1.51	-1.71	-1.51	-1.16	2
2-acylglycerol O-acyltransferase 2-A	1.17	1.34	-1.10	-1.20	-1.41	-1.64	-1.46	-1.12	2
2-amino-3-ketobutyrate coenzyme A ligase. mitochondrial	1.16	1.38	1.21	-1.04	1.06	-1.36	-1.12	-1.23	2
2-methoxy-6-polyprenyl-1.4-benzoquinol methylase. mitochondrial	-1.03	1.12	1.09	-1.09	-1.33	-1.35	-1.23	-1.33	2
39S ribosomal protein L12. mitochondrial	1.03	1.15	1.16	1.01	-1.15	-1.33	-1.09	-1.14	2
39S ribosomal protein L19. mitochondrial	-1.06	-1.09	-1.21	-1.32	-1.38	-1.33	-1.14	-1.18	2
39S ribosomal protein L23. mitochondrial	1.01	-1.04	-1.03	-1.28	-1.27	-1.37	-1.38	-1.14	2
39S ribosomal protein L34. mitochondrial	1.03	-1.13	-1.02	-1.14	-1.37	-1.34	-1.16	-1.11	2
39S ribosomal protein L42. mitochondrial	-1.08	-1.05	-1.08	-1.11	-1.16	-1.27	-1.20	-1.06	2
4-hydroxyphenylpyruvate dioxygenase	-1.12	1.25	1.61	1.47	1.11	-1.58	-2.43	-2.37	2
60S acidic ribosomal protein P0	-1.19	-1.10	-1.60	-2.08	-1.84	-1.87	-1.52	-1.25	2
60S ribosomal export protein NMD3	-1.04	1.05	-1.14	-1.55	-1.52	-1.93	-1.57	-1.41	2
60S ribosomal protein L5-B	1.12	1.20	1.06	-1.06	-1.32	-1.45	-1.13	1.01	2
60S ribosomal protein L7-like 1	1.02	1.06	1.09	-1.21	-1.53	-1.94	-1.37	-1.39	2
60S ribosome subunit biogenesis protein NIP7 homolog	-1.18	-1.12	-1.11	-1.39	-1.66	-2.02	-1.47	-1.39	2
Abhydrolase domain-containing protein 2-A	-1.00	-1.01	-1.25	-1.37	-1.39	-1.42	-1.38	-1.00	2
Abhydrolase domain-containing protein 4	-1.13	1.32	-1.01	-1.34	-1.39	-1.41	-1.72	-1.35	2
Acetyl-coenzyme A synthetase 2-like	1.13	1.35	1.26	1.21	-1.00	-1.30	-1.08	-1.37	2
Actin. non-muscle 6.2	1.19	1.09	1.10	1.11	1.17	1.04	-1.83	-1.07	2
Actin-binding LIM protein 1	-1.01	-1.11	-1.42	-1.41	-1.39	-1.38	-1.36	-1.06	2
Activator of basal transcription 1	-1.03	-1.04	1.02	-1.15	-1.25	-1.58	-1.27	-1.20	2
Acyl-CoA-binding domain-containing protein 4	1.47	1.63	1.75	1.43	-1.21	-1.48	-1.23	-1.22	2
Adenine phosphoribosyltransferase	-1.13	1.01	-1.43	-1.52	-1.43	-1.89	-1.07	-1.04	2
Adenosine deaminase	-1.04	1.02	-1.31	-1.97	-2.43	-2.90	-2.17	-1.41	2
Adenylate kinase isoenzyme 6	-1.04	-1.03	-1.00	-1.04	-1.19	-1.32	-1.23	-1.17	2

Gene Name	Fold-Change								Cluster
	00/24	03/24	06/24	09/24	12/24	15/24	18/21	21/24	
Adenylosuccinate synthetase isozyme 2	1.18	1.40	1.41	1.28	-1.00	-1.44	-1.19	-1.27	2
ADM	1.20	-1.01	1.03	-1.23	-1.88	-2.04	-1.66	-1.55	2
ADP-ribosylation factor-like protein 11	-1.00	1.11	-1.19	-1.39	-1.33	-1.41	-1.64	-1.19	2
ADP-ribosylation factor-like protein 11-like	1.07	1.18	-1.08	-1.35	-1.29	-1.40	-1.58	-1.12	2
Adrenodoxin. mitochondrial	1.09	1.22	1.18	-1.04	-1.10	-1.29	-1.11	-1.11	2
Advillin	1.59	1.58	1.29	1.40	1.08	-1.56	-1.05	-1.17	2
Alcohol dehydrogenase class-3 chain L	1.15	-1.03	-1.21	-1.24	-1.83	-2.86	-1.71	-1.47	2
Alkylated DNA repair protein alkB homolog 8	-1.18	-1.26	-1.43	-1.54	-1.75	-1.74	-1.36	-1.33	2
Alpha3-fucosyltransferase	-1.07	1.10	1.12	-1.00	1.21	-1.36	-1.98	-1.19	2
Alpha-catulin	1.01	1.03	-1.31	-1.66	-1.59	-1.58	-1.20	1.06	2
Amidophosphoribosyltransferase	1.29	1.41	1.07	-1.20	-1.34	-1.50	-1.21	-1.07	2
Ammonium transporter Rh type A	-1.00	1.04	1.02	-1.06	-1.38	-1.42	-1.56	-1.32	2
Angio-associated migratory cell protein	1.15	1.10	1.03	-1.23	-1.49	-1.48	-1.05	-1.11	2
Angiopoietin-related protein 1	1.20	1.09	1.23	-1.13	-1.28	-1.41	-1.35	-1.15	2
Apolipoprotein A-IV-like isoform X1	1.53	1.77	1.53	1.30	1.14	-1.17	-1.13	-1.42	2
Apoptosis regulatory protein Siva	-1.09	-1.29	-1.52	-2.22	-2.02	-1.70	-1.50	-1.43	2
Apoptosis-enhancing nuclease	1.12	-1.16	-1.13	-1.28	-1.65	-1.63	-1.03	-1.07	2
Arf-GAP domain and FG repeats-containing protein 1	-1.22	1.09	-1.09	-1.02	-1.16	-1.32	-1.10	-1.14	2
Ataxin-1	1.09	1.06	-1.19	-1.52	-1.33	-1.76	-1.37	-1.31	2
ATP synthase-coupling factor 6. mitochondrial	-1.20	-1.16	-1.24	-1.51	-2.00	-2.39	-2.02	-1.76	2
ATP-binding cassette sub-family B member 6. mitochondrial	-1.03	1.03	-1.10	-1.39	-1.43	-1.25	-1.08	-1.19	2
ATP-dependent DNA helicase Q4	1.27	1.10	1.08	-1.20	-1.61	-1.48	-1.26	-1.12	2
ATP-dependent RNA helicase DDX18	1.03	-1.05	-1.05	-1.35	-1.71	-2.10	-1.46	-1.40	2
ATP-dependent RNA helicase DDX24	-1.04	-1.07	-1.12	-1.50	-1.80	-2.43	-1.68	-1.35	2
ATP-dependent RNA helicase DDX54	1.01	-1.01	-1.02	-1.17	-1.17	-1.79	-1.23	-1.22	2
ATP-dependent RNA helicase DDX55	1.00	-1.26	-1.65	-1.85	-2.01	-1.83	-1.64	-1.22	2
ATP-dependent RNA helicase DQX1	1.15	1.17	-1.15	-1.29	-1.16	-1.34	-1.04	1.11	2
ATP-dependent zinc metalloprotease YME1L1	1.10	1.35	-1.05	-1.35	-2.09	-2.35	-2.05	-1.57	2
Band 3 anion transport protein	1.34	1.42	1.24	1.27	1.20	-1.05	-1.17	-1.01	2
Bcl-2-related ovarian killer protein homolog B	1.18	1.19	-1.46	-1.60	-2.20	-2.93	-2.18	-1.31	2
Beta.beta-carotene 9'.10'-oxygenase	1.19	1.04	-1.17	-1.45	-2.54	-3.84	-4.19	-1.93	2
Beta-1.4-galactosyltransferase 6	1.03	1.24	1.13	1.12	1.17	-1.04	-1.01	1.02	2
Beta-tectorin	-1.07	1.42	1.09	-1.31	-2.02	-3.07	-1.42	-1.01	2
Beta-tectorin-like	1.16	1.28	1.11	-1.27	-1.43	-1.76	-1.43	-1.05	2
Bicaudal D-related protein 1	1.11	1.01	-1.38	-1.18	-2.04	-1.70	-1.59	-1.47	2
Bifunctional polynucleotide phosphatase/kinase	1.24	1.19	-1.19	-1.55	-2.25	-2.17	-2.00	-1.29	2
Bisphosphoglycerate mutase	1.09	-1.39	-1.19	-1.53	-2.25	-1.72	-1.14	-1.10	2
Brain-specific angiogenesis inhibitor 1-associated protein 2-like	1.15	1.02	1.01	-1.03	1.25	1.01	-1.41	-1.00	2
Calcium-binding mitochondrial carrier protein SCaMC-1-A	1.29	1.24	1.15	-1.26	-1.24	-1.46	1.06	-1.02	2
Calcium-binding mitochondrial carrier protein SCaMC-2-A	-1.38	1.34	1.13	-1.03	-1.97	-4.30	-2.48	-1.43	2
Calpain-2 catalytic subunit	1.17	1.33	1.10	-1.06	1.01	-1.21	-1.51	-1.12	2

Gene Name	Fold-Change								Cluster
	00/24	03/24	06/24	09/24	12/24	15/24	18/21	21/24	
Calpain-8-like	-1.05	1.03	1.09	-1.00	-1.04	-1.13	-2.46	-1.33	2
CapZ-interacting protein	-1.10	-1.12	-1.40	-1.69	-2.08	-1.39	-1.43	-1.05	2
Carbonyl reductase [NADPH] 1	-1.02	1.16	1.23	1.15	1.21	-1.14	-1.11	-1.15	2
CCAAT/enhancer-binding protein zeta	-1.01	-1.15	-1.32	-1.59	-1.80	-2.37	-1.66	-1.55	2
CD109 antigen-like	1.19	1.27	1.29	1.26	1.56	1.03	-1.75	-1.09	2
Cell death activator CIDE-3	1.25	1.60	1.47	1.16	-1.18	-1.68	-1.53	-1.83	2
Cell division control protein 45 homolog	1.21	1.31	1.51	1.05	-1.53	-1.46	-1.37	-1.31	2
Cell division cycle 7-related protein kinase	-1.05	-1.16	-1.05	-1.32	-1.82	-1.58	-1.42	-1.36	2
Centromere protein H	1.14	-1.02	1.09	-1.06	-1.54	-1.11	-1.09	-1.12	2
Centromere protein M	1.25	1.67	1.81	1.61	1.14	1.21	1.05	-1.14	2
Centromere protein T	1.26	1.39	1.87	1.66	1.15	1.22	-1.11	-1.24	2
Chloride intracellular channel protein 5	-1.06	-1.14	-1.26	-1.37	-1.70	-1.64	-1.43	-1.01	2
Cholesterol 25-hydroxylase-like protein	-1.28	-1.25	-1.62	-2.12	-3.53	-3.03	-2.72	-2.33	2
Choline-phosphate cytidylyltransferase A	1.05	1.26	1.16	1.06	-1.28	-1.24	-1.38	-1.24	2
Chromatin assembly factor 1 subunit B	1.24	1.25	1.48	1.33	-1.33	-1.29	-1.33	-1.21	2
Chromosome transmission fidelity protein 18 homolog	1.42	1.55	1.80	1.18	-1.14	-1.33	-1.13	-1.07	2
Chromosome transmission fidelity protein 8 homolog	-1.01	1.10	1.11	-1.13	-1.52	-1.60	-1.31	-1.30	2
Cirhin	1.08	1.12	-1.13	-1.41	-1.63	-1.88	-1.34	-1.24	2
Coiled-coil domain-containing protein 137	-1.04	-1.12	-1.14	-1.38	-1.53	-1.61	-1.26	-1.19	2
Coiled-coil domain-containing protein 86	-1.08	-1.08	1.02	-1.21	-1.51	-1.82	-1.44	-1.35	2
Coiled-coil domain-containing protein 90A. mitochondrial	1.00	1.10	-1.07	-1.20	-1.26	-1.28	-1.17	-1.11	2
Coiled-coil-helix-coiled-coil-helix domain-containing protein 8	1.01	1.08	1.07	1.08	-1.08	-1.10	-1.03	-1.19	2
Collagen alpha-5(IV) chain	1.22	-1.01	-1.00	1.09	-1.01	1.05	-1.24	-1.03	2
Complement C1q tumor necrosis factor-related protein 5	1.13	1.01	-1.04	-1.21	-1.86	-2.01	-2.92	-1.72	2
Complement receptor type 1	1.27	1.21	-1.06	-1.36	-1.31	-1.21	-1.14	1.02	2
Coronin-2A	-1.16	-1.38	-1.64	-1.90	-2.50	-1.84	-2.37	-1.28	2
Crossover junction endonuclease EME1	1.16	1.25	1.35	1.33	-1.06	-1.12	-1.23	-1.17	2
Cryptochrome DASH	1.02	1.33	1.15	-1.15	-1.70	-1.84	-1.64	-1.52	2
Cryptochrome-1	1.07	1.66	1.13	-1.83	-2.71	-3.12	-3.08	-2.49	2
Cryptochrome-2	-1.00	1.79	1.11	-1.86	-2.52	-3.09	-2.09	-1.95	2
CST complex subunit CTC1	-1.07	1.50	1.53	-1.03	1.05	1.16	-1.03	-1.23	2
CTD small phosphatase-like protein 2-A	1.01	1.02	-1.10	-1.38	-1.49	-1.47	-1.47	-1.09	2
Cyclin-dependent kinase 4	-1.04	1.02	1.09	1.11	-1.07	-1.15	-1.07	-1.19	2
Cyclin-dependent kinase 5 activator 2	-1.12	-1.04	-1.69	-2.24	-2.41	-2.80	-2.18	-1.48	2
Cyclin-dependent kinase 9	-1.16	1.14	1.14	1.16	1.11	-1.32	1.00	-1.13	2
Cystathionine gamma-lyase	-1.02	1.10	-1.06	-1.25	-1.48	-1.73	-1.33	-1.31	2
Cystatin-C	-1.20	-1.07	1.05	-1.12	-1.11	-1.11	-1.64	-1.39	2
Cystatin-F	1.25	1.10	-1.08	-1.07	-1.04	-1.14	-1.56	-1.10	2
Cysteine desulfurase. mitochondrial	1.08	1.66	-1.32	-1.34	-1.18	-1.45	1.09	-1.24	2
Cysteine dioxygenase type 1	-1.07	-1.03	-1.54	-2.01	-2.54	-2.02	-1.91	-1.14	2
Cysteine-rich secretory protein 2	1.14	1.58	1.00	1.09	-1.06	-1.58	-2.54	-1.28	2
Cytochrome c iso-1/iso-2	-1.07	1.17	-1.45	-2.01	-1.62	-1.59	-1.53	1.05	2

Gene Name	Fold-Change								Cluster
	00/24	03/24	06/24	09/24	12/24	15/24	18/21	21/24	
Cytochrome c oxidase assembly protein COX15 homolog	1.17	1.54	1.21	1.15	-1.06	-1.10	-1.03	-1.06	2
Cytochrome P450 2B4	-1.18	1.09	-1.19	-1.10	-1.34	-1.97	-1.82	-1.54	2
Cytochrome P450 2C55	1.05	1.05	-1.28	-1.43	-1.88	-2.01	-1.25	-1.05	2
Cytochrome P450 2D26	1.11	1.42	1.15	1.24	-1.09	-1.29	-1.42	-1.39	2
Cytochrome P450 2J1	1.13	1.80	1.13	-1.16	1.01	-1.99	-1.42	-1.56	2
Cytochrome P450 2J6	-1.08	1.28	1.13	1.13	1.03	-1.47	-1.49	-1.69	2
Cytochrome P450 3A27	1.07	1.22	-1.03	1.03	-1.31	-1.35	-1.28	-1.09	2
Cytolysin RTX-A	1.03	-1.01	1.11	-1.11	-1.06	-1.23	-2.14	-1.27	2
Cytolysin Src-1	-1.01	1.00	1.07	-1.17	-1.07	-1.18	-1.78	-1.24	2
DDB1- and CUL4-associated factor 13	-1.05	-1.07	-1.11	-1.36	-1.55	-1.60	-1.41	-1.31	2
Dehydrogenase/reductase SDR family member 1	1.11	1.60	1.31	-1.23	-1.40	-2.46	-2.01	-1.71	2
Dehydrogenase/reductase SDR family member 12	1.00	1.58	1.33	1.13	1.06	-1.41	-1.28	-1.63	2
Dehydrogenase/reductase SDR family member 13-like	1.12	1.34	1.03	-1.30	-1.71	-2.18	-2.34	-1.35	2
DENN domain-containing protein 2C	1.04	1.12	1.05	1.02	1.00	-1.04	-1.34	-1.13	2
Deoxycytidine kinase	1.41	1.61	1.91	1.34	-1.13	-1.08	-1.16	-1.34	2
Deoxynucleotidyltransferase terminal-interacting protein 2	-1.05	-1.07	-1.09	-1.38	-1.62	-1.99	-1.46	-1.26	2
Deoxyribodipyrimidine photo-lyase	-1.18	1.25	1.28	1.24	1.04	-1.20	-1.47	-1.45	2
Deoxyribonuclease-2-beta	1.37	1.09	-1.20	-1.77	-1.75	-1.60	-1.17	1.06	2
Diacylglycerol O-acyltransferase 2	1.07	-1.06	-1.84	-1.93	-1.84	-1.92	-1.85	-1.08	2
Digestive organ expansion factor	-1.05	-1.31	-1.08	-1.77	-1.44	-1.86	-1.57	-1.19	2
Digestive organ expansion factor homolog	-1.03	-1.13	-1.03	-1.50	-1.41	-1.72	-1.36	-1.02	2
Dihydroorotate dehydrogenase (quinone). mitochondrial	1.05	1.25	1.31	1.24	1.05	-1.04	1.04	-1.15	2
Dimethyladenosine transferase	-1.03	-1.14	-1.26	-1.50	-1.47	-1.73	-1.39	-1.17	2
Dimethyladenosine transferase 1. mitochondrial	-1.21	-1.20	-1.20	-1.32	-1.43	-1.56	-1.15	-1.22	2
Dimethyladenosine transferase 2. mitochondrial	1.00	-1.01	-1.24	-1.44	-1.46	-1.66	-1.16	-1.05	2
Diphthine synthase-like	-1.04	1.05	1.05	-1.25	-1.33	-1.75	-1.58	-1.33	2
DNA cross-link repair 1A protein	-1.41	1.48	-1.08	-1.72	-1.75	-1.29	-1.07	-1.65	2
DNA endonuclease RBBP8	1.23	1.30	1.40	1.22	-1.50	-1.18	-1.18	-1.45	2
DNA mismatch repair protein Msh2	1.39	1.49	1.56	1.17	-1.33	-1.80	-1.24	-1.21	2
DNA mismatch repair protein Msh6	1.44	1.44	1.43	-1.10	-1.25	-1.77	-1.16	-1.11	2
DNA polymerase alpha catalytic subunit	1.33	1.25	1.31	1.12	-1.54	-1.58	-1.28	1.01	2
DNA polymerase alpha subunit B	1.43	1.37	1.39	1.06	-1.25	-1.35	-1.11	-1.02	2
DNA polymerase delta catalytic subunit	1.29	1.31	1.32	-1.21	-1.58	-1.86	-1.43	-1.15	2
DNA polymerase delta subunit 3	1.20	1.02	1.14	-1.05	-1.37	-1.26	-1.07	-1.13	2
DNA polymerase epsilon subunit 2	1.14	1.21	1.28	-1.09	-1.70	-1.66	-1.20	-1.10	2
DNA polymerase epsilon subunit 4	-1.01	-1.22	-1.31	-1.59	-1.71	-1.68	-1.31	-1.18	2
DNA polymerase subunit gamma-2. mitochondrial	1.09	1.01	1.01	-1.09	-1.19	-1.32	-1.20	-1.19	2
DNA primase large subunit	1.05	1.05	1.15	-1.04	-1.56	-1.55	-1.38	-1.19	2
DNA primase small subunit	1.28	1.18	1.29	1.05	-1.37	-1.48	-1.14	-1.14	2
DNA repair and recombination protein RAD54B	1.19	1.24	1.63	1.49	-1.08	1.02	-1.27	-1.27	2

Gene Name	Fold-Change								Cluster
	00/24	03/24	06/24	09/24	12/24	15/24	18/21	21/24	
DNA repair and recombination protein RAD54-like	1.32	1.58	1.64	1.41	-1.15	-1.05	-1.26	-1.29	2
DNA repair protein complementing XP-C cells	-1.01	1.42	-1.08	-1.08	-1.27	-1.13	-1.19	-1.24	2
DNA repair protein RAD51 homolog B	1.08	1.31	1.61	1.34	-1.23	-1.28	-1.35	-1.44	2
DNA repair protein RAD52 homolog	1.06	1.19	1.42	1.36	-1.16	-1.06	-1.39	-1.61	2
DNA replication complex GINS protein PSF1	1.07	1.11	1.42	1.07	-1.31	-1.38	-1.26	-1.24	2
DNA replication complex GINS protein PSF2	1.21	1.10	1.08	-1.27	-2.01	-1.86	-1.36	-1.18	2
DNA replication licensing factor MCM3	1.32	1.14	-1.06	-1.37	-1.86	-1.86	-1.26	-1.07	2
DNA replication licensing factor MCM4	1.09	-1.07	-1.10	-1.37	-2.60	-2.08	-1.46	1.06	2
DNA replication licensing factor MCM5	1.33	1.07	1.07	-1.57	-2.09	-2.23	-1.54	-1.13	2
DNA replication licensing factor MCM6	1.40	1.08	1.13	-1.31	-1.79	-1.96	-1.43	-1.07	2
DNA-directed RNA polymerase I subunit RPA1	-1.15	-1.37	-1.52	-1.90	-2.10	-2.18	-1.61	-1.31	2
DNA-directed RNA polymerase I subunit RPA12	-1.10	-1.10	-1.13	-1.37	-1.38	-1.53	-1.32	-1.27	2
DNA-directed RNA polymerase I subunit RPA49	1.14	1.06	-1.02	-1.36	-1.68	-1.89	-1.35	-1.21	2
DNA-directed RNA polymerase III subunit RPC2	1.09	-1.10	-1.07	-1.21	-1.43	-1.52	-1.16	-1.17	2
DNA-directed RNA polymerase III subunit RPC3	-1.05	-1.16	-1.30	-1.42	-1.51	-1.51	-1.24	-1.18	2
DNA-directed RNA polymerases I and III subunit RPAC1	1.01	1.02	1.06	-1.10	-1.29	-1.57	-1.12	-1.15	2
DnaJ homolog subfamily A member 3. mitochondrial	1.15	1.17	1.08	-1.04	-1.10	-1.36	-1.11	-1.14	2
DnaJ homolog subfamily C member 22	-1.10	1.23	1.14	1.28	1.10	-1.05	-1.39	-1.28	2
DnaJ homolog subfamily C member 4	-1.16	-1.30	-1.27	-1.56	-2.26	-2.17	-1.76	-1.39	2
DnaJ homolog subfamily C member 9	1.04	1.09	1.40	1.12	-1.14	-1.23	-1.08	-1.06	2
DPH3 homolog	1.02	-1.10	-1.06	-1.45	-1.27	-1.74	-1.23	-1.15	2
Drebrin-like protein	1.00	-1.02	-1.31	-1.54	-1.51	-1.55	-1.45	-1.18	2
E3 ubiquitin-protein ligase Itchy homolog	-1.32	-1.37	-2.12	-2.17	-2.38	-2.32	-1.63	-1.41	2
E3 ubiquitin-protein ligase RAD18	-1.09	-1.05	-1.18	-1.17	-1.32	-1.29	-1.10	-1.13	2
E3 ubiquitin-protein ligase RNF13	-1.07	-1.08	-1.52	-1.71	-1.69	-1.69	-1.38	-1.26	2
Ectodysplasin-A receptor-associated adapter protein-like isoform X1	1.11	1.12	-1.24	-1.34	-1.26	-1.51	-1.14	-1.16	2
Ectonucleotide pyrophosphatase/phosphodiesterase family member 7	1.23	1.08	-1.14	-1.49	-1.93	-1.90	-1.83	-1.20	2
EH domain-containing protein 2	1.08	1.03	-1.01	-1.29	-1.36	-1.42	-1.16	-1.01	2
Elongation factor 1-alpha 2	1.27	1.22	-1.07	-1.02	-1.12	-1.45	-2.20	-1.16	2
Elongation factor G. mitochondrial	1.01	1.28	1.29	1.13	-1.01	-1.27	-1.06	-1.18	2
Endophilin-A1	1.09	1.07	-1.10	-1.18	-1.17	-1.32	-1.15	1.08	2
Endothelin-converting enzyme 2	-1.07	-1.09	-1.18	-1.64	-2.17	-2.19	-1.53	-1.48	2
Envoplakin-like	1.11	1.11	-1.26	-1.39	-1.27	-1.56	-1.23	1.13	2
Ephrin-A1	1.14	-1.10	-1.53	-1.64	-1.88	-1.51	-1.48	-1.02	2
Epidermal growth factor receptor kinase substrate 8-like	1.15	-1.04	-1.24	-1.46	-1.44	-1.25	-1.66	-1.09	2
Epidermal growth factor receptor kinase substrate 8-like protein 1-like	1.18	1.13	-1.25	-1.51	-1.45	-1.29	-1.29	1.07	2
Epidermal growth factor receptor kinase substrate 8-like protein 2	1.36	1.46	1.23	1.03	-1.23	-1.21	-1.20	-1.05	2
Epimerase family protein SDR39U1	1.08	1.47	1.19	-1.16	-1.48	-1.30	-1.49	-1.46	2
Equilibrative nucleoside transporter 1	1.04	1.05	1.14	-1.08	-1.20	-1.23	1.05	1.00	2
Erythroid membrane-associated protein	-1.01	1.01	-1.07	-1.16	-1.15	-1.51	-1.55	-1.22	2

Gene Name	Fold-Change								Cluster
	00/24	03/24	06/24	09/24	12/24	15/24	18/21	21/24	
Ethanolamine-phosphate cytidylyltransferase	-1.10	1.12	1.17	-1.09	-1.36	-2.50	-2.01	-1.54	2
Eukaryotic initiation factor 4A-II	1.10	1.13	-1.06	-1.19	-1.10	-1.59	-1.33	-1.10	2
Eukaryotic translation elongation factor 1 epsilon-1	-1.04	-1.05	1.00	-1.10	-1.31	-1.27	-1.06	-1.08	2
Eukaryotic translation initiation factor 4E type 3	1.03	1.07	-1.09	-1.43	-1.74	-1.60	-1.20	-1.19	2
Eukaryotic translation initiation factor 6	-1.07	-1.02	-1.00	-1.07	-1.29	-1.48	-1.31	-1.29	2
Exosome complex component CSL4	-1.07	-1.23	-1.88	-2.55	-2.15	-2.06	-2.06	-1.37	2
Exosome complex component RRP4	1.01	1.00	1.16	1.06	-1.10	-1.31	-1.12	-1.14	2
Exosome complex component RRP41	-1.05	1.02	1.14	-1.06	-1.28	-1.56	-1.29	-1.34	2
Exosome complex component RRP45	-1.16	-1.08	1.01	-1.20	-1.34	-1.54	-1.10	-1.13	2
Exosome complex exonuclease RRP44	1.18	-1.09	-1.13	-1.46	-1.28	-1.46	1.03	-1.11	2
Extracellular matrix protein 2	1.29	-1.04	1.16	-1.05	-1.09	-1.06	-1.73	-1.22	2
Fanconi anemia group D2 protein	1.37	1.73	2.13	2.00	1.23	1.12	-1.11	-1.16	2
Fanconi anemia group I protein	1.29	1.43	1.67	1.13	-1.19	-1.22	-1.39	-1.36	2
FAST kinase domain-containing protein 3	1.04	1.19	-1.40	-1.64	-1.56	-1.54	-1.62	-1.21	2
F-box only protein 42	1.10	1.16	1.16	1.09	1.06	-1.01	-1.02	-1.05	2
F-box/LRR-repeat protein 14	1.08	1.16	1.06	1.03	-1.03	-1.16	-1.16	-1.03	2
F-box/LRR-repeat protein 5	1.08	1.18	1.01	-1.33	-1.33	-1.52	-1.10	1.13	2
Ferrochelatase. mitochondrial	1.04	1.82	-1.17	-1.55	-1.49	-2.34	-1.96	-1.51	2
Fibroblast growth factor 23	-1.09	1.07	-1.05	-1.66	-1.85	-1.98	-1.63	-1.46	2
Fibroleukin	-1.20	-1.25	-1.52	-1.87	-2.15	-2.31	-1.89	-1.40	2
Flotillin-1	-1.06	-1.03	1.19	1.17	-1.00	-1.05	-1.23	-1.20	2
Folylpolyglutamate synthase. mitochondrial	1.02	1.01	-1.39	-1.94	-2.11	-2.06	-1.15	-1.06	2
Forkhead box protein E1	-1.24	2.47	2.14	-1.11	1.34	1.07	1.54	1.46	2
G patch domain-containing protein 4	-1.26	-1.33	-1.03	-1.41	-1.63	-1.99	-1.34	-1.26	2
G1/S-specific cyclin-D1	-1.10	-1.01	-1.14	-1.20	-1.27	-1.36	-1.26	-1.17	2
Gamma-glutamyltranspeptidase 1	1.51	1.50	-1.05	-1.34	-1.69	-2.08	-1.36	1.01	2
Gamma-tubulin complex component 2	1.05	1.10	1.10	-1.02	-1.25	-1.23	-1.23	-1.25	2
Ganglioside GM2 activator	1.36	1.46	1.09	-1.09	-1.05	-1.01	-2.05	-1.17	2
Gap junction gamma-2 protein	1.01	-1.04	-1.18	-1.37	-1.46	-1.37	-1.32	1.05	2
Gem-associated protein 4	1.05	1.17	1.15	-1.07	-1.08	-1.30	-1.15	-1.13	2
Gliomedin	-1.31	-1.29	-1.45	-2.06	-1.93	-3.15	-3.11	-1.34	2
Glucose-6-phosphatase	-1.50	-1.02	-1.37	-2.32	-3.09	-2.69	-2.01	-1.52	2
Glucose-6-phosphatase 2	-1.50	1.13	1.55	1.05	-1.57	-1.90	-4.50	-2.43	2
Glutamate-rich WD repeat-containing protein 1	1.09	1.11	1.14	-1.14	-1.38	-1.92	-1.26	-1.31	2
Glutamyl aminopeptidase	1.88	1.65	-1.02	-1.27	-1.95	-1.95	-1.62	1.11	2
Glycerol-3-phosphate dehydrogenase 1-like protein	-1.03	1.08	1.21	1.12	-1.04	-1.12	-1.15	-1.08	2
Glycoprotein gp2-like	1.18	1.38	-1.30	-1.55	-1.83	-2.03	-1.14	1.03	2
Glypican-1	1.26	1.21	1.15	1.02	1.06	-1.06	-1.06	-1.00	2
Golgi pH regulator	1.03	1.02	-1.15	-1.33	-1.74	-1.93	-1.50	-1.20	2
Growth factor receptor-bound protein 10-like	-1.29	-1.39	-1.44	-1.56	-1.96	-1.98	-1.42	-1.49	2
GrpE protein homolog 1. mitochondrial	1.02	1.17	1.21	1.02	-1.12	-1.41	-1.06	-1.23	2
GTP cyclohydrolase 1 feedback regulatory protein	-1.10	1.08	1.15	-1.02	1.07	-1.13	-1.32	-1.31	2
GTPase IMAP family member 8-like	1.25	-1.08	-1.13	-1.40	-1.41	-1.02	-1.82	-1.08	2

Gene Name	Fold-Change								Cluster
	00/24	03/24	06/24	09/24	12/24	15/24	18/21	21/24	
Guanine nucleotide-binding protein-like 3	-1.02	1.10	-1.03	-1.45	-1.65	-2.22	-1.29	-1.25	2
Guanine nucleotide-binding protein-like 3-like protein	1.01	-1.01	-1.02	-1.26	-1.42	-1.62	-1.30	-1.20	2
H/ACA ribonucleoprotein complex non-core subunit NAF1	-1.12	-1.28	-1.51	-1.75	-1.92	-1.85	-1.43	-1.24	2
H/ACA ribonucleoprotein complex subunit 1	1.14	1.13	1.15	-1.06	-1.34	-1.59	-1.19	-1.19	2
H/ACA ribonucleoprotein complex subunit 3	-1.02	-1.01	1.02	-1.08	-1.42	-1.74	-1.38	-1.27	2
H/ACA ribonucleoprotein complex subunit 4	-1.09	-1.22	-1.09	-1.28	-1.51	-2.07	-1.50	-1.34	2
H-2 class II histocompatibility antigen. A-K alpha chain	1.55	-1.09	-1.07	-1.29	-1.37	-1.06	-1.20	1.01	2
HAUS augmin-like complex subunit 1	1.12	1.06	1.12	-1.07	-1.44	-1.12	-1.15	-1.19	2
Heme oxygenase 2	-1.05	1.01	1.01	-1.28	-1.49	-1.62	-1.66	-1.40	2
Hepatic triacylglycerol lipase	1.04	-1.07	-1.17	-1.73	-2.35	-1.61	-1.37	-1.01	2
Hephaestin-like protein 1	1.06	1.11	-1.12	-1.21	-1.45	-1.46	-1.02	1.08	2
Heterogeneous nuclear ribonucleoprotein A/B	-1.22	-1.19	-1.14	-1.39	-1.93	-2.03	-1.80	-1.21	2
HIG1 domain family member 1A	-1.41	-1.40	-1.37	-1.68	-2.43	-2.46	-2.59	-1.44	2
High affinity cationic amino acid transporter 1	-1.06	1.16	1.28	-1.07	-2.35	-3.70	-3.51	-2.44	2
High choriolytic enzyme 2	1.35	2.13	1.46	1.33	1.12	-2.25	-1.43	-1.07	2
High mobility group nucleosome-binding domain-containing protein 3	-1.03	-1.10	-1.06	-1.35	1.09	1.03	-1.49	-1.17	2
HIRA-interacting protein 3	1.12	1.20	1.40	1.24	-1.08	-1.24	-1.11	-1.15	2
Histidine protein methyltransferase 1 homolog	-1.20	-1.24	-1.31	-1.98	-1.69	-2.03	-1.38	-1.23	2
Histone acetyltransferase KAT2A	-1.22	-1.04	-1.21	-1.40	-2.00	-1.63	-1.13	1.12	2
Histone acetyltransferase KAT7	-1.01	-1.20	-1.46	-1.67	-1.75	-1.80	-1.41	-1.10	2
Histone acetyltransferase type B catalytic subunit	1.06	1.12	1.26	1.16	-1.03	-1.09	-1.06	-1.07	2
Histone chaperone asf1b-B	1.27	1.62	1.56	1.33	-1.05	-1.12	1.25	1.04	2
Histone RNA hairpin-binding protein	1.14	1.24	1.30	1.03	-1.26	-1.45	-1.22	-1.25	2
Histone-binding protein RBBP4	1.08	1.05	1.43	1.27	-1.04	-1.10	-1.01	-1.10	2
Importin-7	1.18	1.17	1.08	-1.08	-1.18	-1.51	-1.17	-1.14	2
Inactive rhomboid protein 1	-1.21	-1.10	1.14	-1.05	-1.13	-1.20	-1.33	-1.18	2
Influenza virus NS1A-binding protein homolog A	-1.19	1.13	1.35	1.32	1.15	-1.38	-1.45	-1.41	2
Inositol-3-phosphate synthase 1-A	1.10	1.36	-1.00	-1.18	-1.95	-2.87	-2.03	-1.56	2
Interferon regulatory factor 1	1.57	-1.05	1.03	-1.17	-1.78	-1.16	-1.24	-1.27	2
Intermediate filament protein ON3-like	1.18	1.32	-1.29	-1.42	-1.27	-2.32	-2.12	-1.51	2
Intracellular hyaluronan-binding protein 4	1.28	1.19	-1.02	-1.20	-1.93	-2.24	-1.64	1.00	2
Iron-sulfur cluster assembly 1 homolog. mitochondrial	-1.07	1.11	-1.22	-1.38	-1.46	-1.31	-1.24	-1.16	2
Isobutyryl-CoA dehydrogenase. mitochondrial	-1.08	1.08	-1.12	-1.15	-1.43	-1.54	-1.56	-1.36	2
Jun dimerization protein 2	1.04	-1.05	-1.15	-1.51	-2.35	-1.98	-1.63	1.05	2
Kelch domain-containing protein 4	1.02	1.04	1.06	-1.11	-1.28	-1.32	-1.19	-1.14	2
Keratin. type I cytoskeletal 19	1.08	1.11	-1.01	-1.43	-1.59	-1.80	-1.43	-1.27	2
Keratin. type II cytoskeletal	1.50	1.56	1.13	-1.03	-1.34	-1.86	-2.71	-1.71	2
Keratin. type II cytoskeletal 8	1.17	1.21	1.24	1.37	1.11	1.06	-1.12	-1.17	2
Kinesin-like protein KIF15	1.19	1.18	1.45	1.42	-1.05	1.29	-1.11	-1.19	2
Kinetochore-associated protein 1	1.49	1.73	2.00	1.64	1.25	1.10	-1.31	-1.21	2
KRR1 small subunit processome component homolog	1.10	1.03	1.02	-1.03	-1.27	-1.31	-1.14	-1.05	2

Gene Name	Fold-Change								Cluster
	00/24	03/24	06/24	09/24	12/24	15/24	18/21	21/24	
Krueppel-like factor 6	-1.03	-1.19	-1.35	-1.78	-1.73	-1.80	-1.47	-1.26	2
Kynurenine--oxoglutarate transaminase 1. mitochondrial	-1.12	-1.06	-1.11	-1.53	-1.71	-1.62	-1.31	-1.26	2
La-related protein 4	-1.00	-1.01	-1.55	-1.95	-1.92	-2.75	-1.55	-1.10	2
Leucine-rich glioma-inactivated protein 1	1.14	1.43	1.15	-1.01	1.06	-1.07	-1.11	1.14	2
Leucine-rich repeat neuronal protein 4	-1.02	-1.04	-1.08	-1.35	-1.36	-1.24	-1.18	-1.04	2
Leucine-rich repeat-containing protein 58	-1.13	1.01	-1.32	-1.18	-2.43	-1.99	-1.59	-1.69	2
Lipocalin	1.20	-1.22	1.20	1.17	-1.15	1.31	-2.06	-1.38	2
LMBR1 domain-containing protein 2-B	-1.06	-1.02	-1.35	-1.80	-2.43	-2.77	-2.45	-1.53	2
LON peptidase N-terminal domain and RING finger protein 1	-1.12	1.33	-1.33	-2.10	-2.72	-4.71	-3.69	-2.12	2
LON peptidase N-terminal domain and RING finger protein 3	-1.15	4.83	5.07	6.32	3.79	-1.99	-2.71	-2.35	2
Low affinity vacuolar monovalent cation/H(+) antiporter	1.00	1.05	1.03	-1.17	-1.31	-1.21	-1.17	-1.16	2
Lymphatic vessel endothelial hyaluronic acid receptor 1	-1.58	-1.48	-1.10	-2.27	-3.50	-4.14	-2.20	-1.33	2
Lysine-specific demethylase NO66	-1.07	-1.11	1.03	-1.14	-1.31	-1.46	-1.23	-1.23	2
LysM and putative peptidoglycan-binding domain-containing protein 3	1.05	1.08	-1.03	-1.18	-1.28	-1.46	-1.44	-1.19	2
Lysophosphatidic acid phosphatase type 6	-1.00	1.12	1.09	1.04	-1.11	-1.19	-1.10	-1.16	2
Lysozyme C. milk isozyme-like	1.09	1.29	1.18	-1.00	1.14	-1.05	-2.09	-1.34	2
Magnesium transporter MRS2 homolog. mitochondrial	1.03	-1.06	-1.15	-1.28	-1.38	-1.37	-1.12	-1.03	2
MAGUK p55 subfamily member 6	1.06	1.42	1.29	1.10	-1.27	-1.76	-1.26	-1.31	2
Mdm2-binding protein	1.33	1.47	1.24	1.14	1.07	1.11	1.02	-1.51	2
Meiotic nuclear division protein 1 homolog	1.16	1.36	1.40	1.05	-1.66	-1.70	-1.67	-1.56	2
Melanopsin-A-like	1.06	1.61	1.64	1.40	1.14	-1.42	-2.20	-1.06	2
Metallo-beta-lactamase domain-containing protein 1	-1.09	-1.01	-1.10	-1.41	-1.40	-1.53	-1.27	-1.27	2
Methionine aminopeptidase 1	1.09	-1.01	1.07	1.00	-1.13	-1.30	-1.15	-1.13	2
Methylmalonic aciduria and homocystinuria type D homolog. mitochondrial	-1.14	-1.12	-1.19	-1.57	-1.76	-1.93	-1.63	-1.16	2
Methyltransferase-like protein 10	-1.19	-1.25	-1.36	-1.47	-1.61	-1.60	-1.25	-1.15	2
Methyltransferase-like protein LOC121952 homolog	1.09	1.03	1.25	-1.01	-1.73	-1.54	-1.15	-1.52	2
Microfibrillar-associated protein 2	1.04	-1.05	-1.10	-1.30	-1.52	-1.26	-1.13	-1.10	2
Microsomal glutathione S-transferase 2	1.18	1.40	1.30	-1.05	-1.12	-1.54	-4.94	-1.72	2
Mid1-interacting protein 1-B	1.07	1.25	1.28	1.11	1.18	-1.21	-1.07	-1.08	2
Mid1-interacting protein 1-like	1.34	1.17	1.40	-1.12	1.11	-1.80	-2.62	-1.52	2
Midnolin-like	-1.24	1.18	-1.17	-1.23	-1.06	-1.29	-1.12	-1.24	2
Mitochondrial carnitine/acylcarnitine carrier protein CACL	-1.19	-1.00	-1.56	-1.81	-2.05	-2.04	-1.20	-1.20	2
Mitochondrial glutamate carrier 2	1.28	1.50	1.09	-1.48	-1.51	-2.13	-1.21	1.02	2
Mitochondrial import inner membrane translocase subunit tim16	-1.09	-1.04	-1.25	-1.50	-1.68	-1.68	-1.45	-1.24	2
Mitochondrial import receptor subunit TOM20 homolog	-1.08	-1.05	-1.14	-1.39	-1.48	-2.20	-1.43	-1.19	2
Mitochondrial ribonuclease P protein 1	1.06	-1.12	-1.18	-1.46	-1.36	-1.42	-1.11	-1.01	2
Mitogen-activated protein kinase 13	-1.12	-1.07	-1.02	-1.36	-1.18	-1.19	-1.38	-1.00	2
Mitogen-activated protein kinase 14B	1.22	1.17	-1.00	-1.07	-1.10	-1.22	-1.55	1.02	2

Gene Name	Fold-Change								Cluster
	00/24	03/24	06/24	09/24	12/24	15/24	18/21	21/24	
Mitogen-activated protein kinase kinase 6	1.06	1.13	1.06	-1.27	-1.59	-1.71	-1.88	-1.27	2
MKI67 FHA domain-interacting nucleolar phosphoprotein	-1.12	-1.02	-1.14	-1.32	-1.48	-1.87	-1.19	-1.26	2
MKL/myocardin-like protein 1	1.06	1.10	-1.30	-1.61	-1.58	-1.39	-1.33	1.01	2
MOSC domain-containing protein 1. mitochondrial	-1.11	-1.02	1.08	-1.18	-1.19	-1.29	-1.34	-1.22	2
MOSC domain-containing protein 2. mitochondrial	-1.20	1.05	-1.14	-1.89	-2.52	-2.93	-2.40	-1.53	2
M-phase phosphoprotein 6	1.02	-1.06	1.03	-1.14	-1.20	-1.41	-1.27	-1.19	2
MRNA turnover protein 4 homolog	-1.05	-1.02	1.04	-1.20	-1.43	-1.76	-1.23	-1.27	2
Myb-related protein B	1.33	1.41	1.39	1.13	-1.51	-1.68	-1.34	-1.20	2
MYC-induced nuclear antigen	1.08	1.13	1.16	1.03	-1.01	-1.24	-1.12	-1.12	2
Myeloid-associated differentiation marker homolog	1.05	1.16	1.12	-1.13	-1.41	-1.45	-1.62	-1.24	2
Myocilin	1.15	1.25	-1.44	-1.42	-1.48	-1.68	-1.16	-1.04	2
N(6)-adenine-specific DNA methyltransferase 2	-1.17	-1.04	1.01	-1.27	-1.36	-1.68	-1.42	-1.31	2
N-acetyltransferase 10	1.00	1.01	1.13	-1.02	-1.24	-1.70	-1.11	-1.20	2
N-acetyltransferase ESCO2	1.14	1.47	2.01	1.76	1.06	-1.05	-1.13	-1.43	2
NADH-cytochrome b5 reductase 2	1.21	1.28	1.53	1.42	-1.20	-1.10	-1.10	-1.24	2
Nascent polypeptide-associated complex subunit alpha. muscle-specific form	1.15	1.14	1.02	-1.11	1.04	-1.21	-1.55	-1.19	2
N-chimaerin	-1.07	1.20	-1.06	-1.18	-1.09	-1.19	-1.23	-1.14	2
NEDD4 family-interacting protein 1-like	-1.06	-1.10	-1.16	-1.45	-1.55	-1.61	-1.51	-1.19	2
Neurogenic differentiation factor 1	1.26	2.52	3.51	3.18	1.97	1.03	-1.20	-1.16	2
Neurogenic differentiation factor 6-A	1.27	2.55	3.22	3.09	1.89	-1.03	-1.13	-1.14	2
Neuroguidin	-1.20	1.01	1.07	-1.03	-1.35	-1.46	-1.25	-1.03	2
Neuropeptide Y receptor type 1	1.16	1.45	1.03	-1.18	-1.47	-1.37	1.09	1.17	2
NHP2-like protein 1	-1.16	-1.17	-1.02	-1.18	-1.50	-1.81	-1.35	-1.39	2
Nicotinate phosphoribosyltransferase	-1.16	-1.02	-1.25	-1.41	-1.58	-1.78	-1.56	-1.33	2
NIPA-like protein	1.15	1.17	1.35	1.08	-1.23	-1.29	-1.24	-1.15	2
N-lysine methyltransferase setd6	1.02	1.09	1.27	1.12	-1.00	-1.22	1.04	-1.07	2
Nocturnin	-1.03	-1.04	-1.32	-1.67	-1.90	-1.75	-1.45	-1.26	2
Notchless protein homolog 1	-1.04	-1.06	-1.14	-1.30	-1.49	-2.18	-1.29	-1.02	2
Nuclear autoantigenic sperm protein	1.32	1.21	1.47	1.12	-1.78	-1.88	-1.38	-1.33	2
Nuclear factor erythroid 2-related factor 2	1.05	-1.08	-1.30	-1.50	-1.49	-1.53	-1.55	-1.41	2
Nuclear pore complex protein Nup107	1.18	1.17	1.46	1.32	1.13	1.04	-1.01	-1.0	2
Nuclear pore complex protein Nup155	1.04	1.13	1.16	-1.10	-1.40	-1.73	-2.22	-1.39	2
Nuclear pore glycoprotein p62	1.13	1.15	1.38	1.16	1.01	-1.23	-1.07	-1.18	2
Nuclear receptor ROR-beta-like	-1.16	2.29	2.89	1.81	-1.58	-3.00	-6.07	-4.01	2
Nuclear receptor ROR-gamma	1.11	1.33	1.02	-1.27	-1.81	-2.60	-3.06	-1.50	2
Nuclear receptor subfamily 5 group A member 2	-1.05	1.07	1.15	1.16	1.00	-1.43	-1.57	-1.25	2
Nucleolar and coiled-body phosphoprotein 1	-1.06	-1.05	-1.05	-1.20	-1.56	-2.41	-1.49	-1.187	2
Nucleolar complex protein 2 homolog	1.02	1.03	-1.01	-1.27	-1.46	-2.14	-1.30	-1.264	2
Nucleolar complex protein 4 homolog	-1.13	-1.26	-1.18	-1.44	-1.71	-1.92	-1.51	-1.364	2
Nucleolar GTP-binding protein 2	-1.17	-1.28	-1.63	-1.99	-2.20	-2.37	-1.37	-1.021	2
Nucleolar MIF4G domain-containing protein 1	1.06	-1.23	-1.18	-1.22	-1.64	-1.77	-1.27	-1.16	2

Gene Name	Fold-Change								Cluster
	00/24	03/24	06/24	09/24	12/24	15/24	18/21	21/24	
Nucleolar pre-ribosomal-associated protein 1	-1.00	-1.12	-1.24	-1.52	-1.82	-1.92	-1.43	-1.35	2
Nucleolar protein 11-like	-1.31	-1.42	-1.15	-1.41	-1.53	-1.94	-1.64	-1.37	2
Nucleolar protein 12	-1.01	-1.18	-1.23	-1.45	-1.64	-1.88	-1.33	-1.22	2
Nucleolar protein 14	1.03	1.08	-1.12	-1.41	-1.32	-1.82	-1.24	-1.19	2
Nucleolar protein 56	-1.22	-1.41	-1.34	-1.73	-1.88	-2.11	-1.72	-1.32	2
Nucleolar protein 58	-1.20	-1.32	-1.27	-1.60	-1.89	-2.59	-1.79	-1.39	2
Nucleolar protein 6	1.12	1.04	1.02	-1.29	-1.48	-1.95	-1.35	-1.29	2
Nucleolar protein 6-like	1.06	1.04	1.03	-1.20	-1.57	-1.96	-1.22	-1.21	2
Nucleolar protein 8	-1.13	-1.23	-1.00	-1.35	-1.81	-1.58	-1.50	-1.10	2
Nucleolin	1.05	1.15	1.27	1.13	-1.12	-1.31	-1.10	-1.13	2
Nucleophosmin	-1.23	-1.11	1.01	-1.28	-1.42	-1.81	-1.31	-1.42	2
Olfactomedin-like protein 2A	1.01	1.18	1.29	-1.04	-1.27	-1.36	-1.76	-1.49	2
Oocyte zinc finger protein XlCOF19	-1.08	-1.16	-1.17	-1.17	-1.25	-1.46	-1.31	-1.23	2
Opioid-binding protein/cell adhesion molecule	1.01	1.34	-1.18	-1.28	-1.25	-1.51	-1.12	1.04	2
Opsin-5-like	-1.10	-1.12	-1.51	-1.77	-2.01	-1.95	-1.61	-1.09	2
Origin recognition complex subunit 2	1.25	1.16	1.09	-1.09	-1.57	-1.64	-1.32	-1.1	2
Origin recognition complex subunit 3	1.24	1.08	1.01	-1.24	-1.73	-1.59	-1.32	-1.25	2
Origin recognition complex subunit 4	1.24	1.29	1.38	1.05	-1.50	-1.85	-1.24	-1.14	2
Origin recognition complex subunit 5	-1.01	1.02	1.07	-1.30	-1.86	-2.04	-1.66	-1.44	2
Ornithine decarboxylase	-1.03	1.33	1.52	1.43	1.11	-1.25	-1.18	-1.42	2
Otopetrin-2	-1.00	1.10	-1.17	-1.24	-1.14	-1.65	-1.92	-1.41	2
OTU domain-containing protein 3	1.12	1.12	-1.06	-1.35	-1.42	-1.38	-1.20	-1.16	2
Oxidoreductase NAD-binding domain-containing protein 1	-1.12	-1.10	-1.57	-1.53	-1.55	-1.55	-1.60	-1.25	2
P21-activated protein kinase-interacting protein 1-like	-1.08	-1.17	1.01	-1.21	-1.51	-1.84	-1.31	-1.33	2
Pantothenate kinase 2. mitochondrial	-1.03	-1.10	-1.14	-1.37	-1.28	-1.39	-1.24	-1.08	2
PDZ and LIM domain protein 5	-1.12	-1.27	-1.77	-2.18	-2.31	-2.51	-1.99	-1.40	2
Peptidoglycan-recognition protein SC2	-1.29	1.13	-1.35	-1.92	-1.64	-2.49	-2.03	-1.10	2
Period circadian protein homolog 1	-1.06	1.27	-1.19	-1.05	-1.44	-3.00	-2.88	-2.18	2
Period circadian protein homolog 2	-1.05	1.76	-1.40	-1.30	-2.14	-2.78	-1.75	-1.81	2
Periodic tryptophan protein 1 homolog	1.08	1.03	1.11	-1.10	-1.39	-1.68	-1.30	-1.21	2
Periplakin	-1.01	1.05	-1.03	-1.28	-1.64	-1.75	-1.66	-1.13	2
Periplakin-like	-1.16	1.15	-1.00	-1.06	-1.45	-1.83	-1.98	-1.23	2
Peroxisomal membrane protein 11A	-1.20	-1.13	1.14	-1.31	-2.26	-2.38	-1.79	1.05	2
Peroxisome proliferator-activated receptor gamma	1.04	1.30	-1.26	-1.99	-1.87	-1.34	-1.30	1.03	2
Peroxisome proliferator-activated receptor gamma coactivator-related protein 1	1.13	1.11	1.23	-1.12	-1.42	-2.07	-1.52	-1.09	2
Pescadillo	1.01	-1.07	-1.26	-1.33	-1.68	-1.72	-1.24	-1.34	2
Phenylalanine-4-hydroxylase	-1.04	1.37	1.30	1.15	-1.03	-1.35	-2.02	-1.67	2
Phosphatidylinositol 3-kinase regulatory subunit alpha	-1.08	-1.19	-1.35	-1.51	-1.70	-1.52	-1.40	-1.01	2
Phosphatidylinositol 4-phosphate 5-kinase-like protein 1	1.05	1.09	-1.16	-1.65	-1.67	-1.89	-1.79	1.04	2
Phosphoglycerate mutase 1	1.23	1.27	1.30	1.25	1.18	1.14	-1.02	1.05	2
Phosphoglycolate phosphatase	-1.08	1.05	-1.01	-1.08	-1.27	-1.37	-1.20	-1.16	2

Gene Name	Fold-Change								Cluster
	00/24	03/24	06/24	09/24	12/24	15/24	18/21	21/24	
Phospholipase B1. membrane-associated	-1.13	1.01	-1.07	-1.39	-1.52	-1.68	-1.73	-1.16	2
Phytanoyl-CoA dioxygenase domain-containing protein 1	1.14	1.27	1.24	1.17	-1.17	-1.23	-1.22	-1.22	2
PIN2/TERF1-interacting telomerase inhibitor 1	-1.11	-1.26	-1.55	-2.11	-2.49	-2.81	-2.08	-1.54	2
Pinopsin-like	1.09	1.24	1.28	1.21	1.15	-1.01	-1.32	-1.04	2
PKHD domain-containing transmembrane protein C17orf101 homolog	1.10	1.13	1.39	1.22	1.24	-1.06	1.00	-1.05	2
Pleckstrin homology domain-containing family G member 5	1.05	-1.06	-1.31	-1.39	-1.42	-1.36	-1.32	1.10	2
Poly [ADP-ribose] polymerase 1	1.18	1.14	1.12	1.07	-1.11	-1.23	-1.09	-1.01	2
Polyamine-modulated factor 1	1.23	1.18	1.30	1.08	-1.35	-1.02	-1.16	-1.25	2
Polycomb group RING finger protein 5	1.11	1.10	-1.22	-1.67	-1.53	-1.59	-1.37	-1.17	2
Potential tRNA (adenine-N(1)-)-methyltransferase catalytic subunit TRMT61B	-1.16	1.00	-1.29	-2.19	-1.97	-2.35	-2.04	-1.51	2
Pre-rRNA-processing protein TSR1 homolog	1.14	1.12	1.10	-1.12	-1.31	-1.69	-1.19	-1.23	2
Pre-rRNA-processing protein TSR2 homolog	-1.01	1.03	1.20	1.14	-1.01	-1.21	-1.14	-1.13	2
Probable ATP-dependent RNA helicase DDX10	-1.42	-1.11	-1.38	-1.88	-1.90	-1.93	-1.23	-1.22	2
Probable ATP-dependent RNA helicase DDX11	1.29	1.42	1.34	-1.09	-1.63	-1.67	-1.48	-1.41	2
Probable ATP-dependent RNA helicase DDX20	-1.10	-1.03	-1.03	-1.18	-1.22	-1.24	-1.13	1.00	2
Probable ATP-dependent RNA helicase DDX27	-1.06	-1.01	-1.05	-1.21	-1.37	-1.65	-1.31	-1.28	2
Probable ATP-dependent RNA helicase DDX28	-1.03	1.03	1.03	-1.12	-1.23	-1.39	-1.06	-1.09	2
Probable ATP-dependent RNA helicase DDX47	-1.03	-1.05	-1.11	-1.31	-1.52	-1.76	-1.39	-1.22	2
Probable ATP-dependent RNA helicase DDX49	-1.06	-1.11	-1.22	-1.56	-1.73	-1.84	-1.31	-1.27	2
Probable ATP-dependent RNA helicase DDX5	1.16	1.30	1.61	1.38	1.38	-1.01	-1.10	-1.19	2
Probable ATP-dependent RNA helicase DDX56	1.03	-1.10	-1.29	-1.68	-1.71	-2.01	-1.33	-1.37	2
Probable dimethyladenosine transferase	-1.09	-1.22	-1.29	-1.50	-1.57	-1.98	-1.45	-1.20	2
Probable RNA-binding protein 19	1.08	1.17	-1.04	-1.11	-1.35	-1.52	1.17	1.02	2
Proliferating cell nuclear antigen	1.17	1.18	1.53	1.27	-1.43	-1.59	-1.40	-1.32	2
Proline synthase co-transcribed bacterial homolog protein	1.00	1.15	1.06	-1.08	-1.14	-1.24	-1.30	-1.29	2
Prostacyclin synthase	1.01	-1.05	-1.12	-1.49	-1.54	-1.86	-1.69	-1.20	2
Prostaglandin E synthase 2	-1.03	1.09	1.16	1.08	-1.08	-1.16	-1.13	-1.15	2
Protein ACN9 homolog. mitochondrial	-1.10	-1.09	-1.20	-1.30	-1.46	-1.37	-1.25	-1.17	2
Protein AHNAK2-like	1.25	1.20	-1.23	-1.41	-1.30	-1.91	-1.38	-1.16	2
Protein arginine N-methyltransferase 3	1.13	1.26	1.30	1.12	-1.18	-1.51	-1.30	-1.30	2
Protein arginine N-methyltransferase 7	1.03	1.09	1.10	1.00	-1.15	-1.37	-1.17	-1.16	2
Protein C16orf88	1.06	-1.02	1.25	-1.00	-1.21	-1.31	-1.11	-1.19	2
Protein ccsmst1	1.05	1.21	-1.09	-1.28	-1.27	-1.31	-1.17	-1.17	2
Protein CNPPD1	-1.10	-1.17	-1.48	-1.61	-1.66	-1.75	-1.53	-1.22	2
Protein FAM136A	-1.03	-1.05	-1.06	-1.10	-1.32	-1.56	-1.20	-1.18	2
Protein FAM161A	-1.12	1.84	1.64	1.49	1.01	-1.28	-1.28	-1.74	2
Protein FAM195A	-1.08	-1.08	-1.34	-1.62	-1.52	-1.72	-1.18	-1.14	2
Protein G7d	1.01	1.10	1.01	-1.30	-1.36	-1.28	-1.65	-1.19	2
Protein HEG	-1.13	-1.23	-1.61	-1.67	-1.95	-1.89	-1.51	-1.29	2
Protein kinase C delta type	1.02	1.11	1.12	1.06	1.07	1.10	-1.28	-1.10	2
Protein LBH	1.01	1.15	1.13	-1.04	-1.18	-1.58	-1.74	-1.73	2
Protein LTV1 homolog	-1.06	1.00	-1.21	-1.39	-1.52	-1.72	-1.31	-1.19	2

Gene Name	Fold-Change								Cluster
	00/24	03/24	06/24	09/24	12/24	15/24	18/21	21/24	
Protein MAK16 homolog	-1.01	-1.06	-1.01	-1.18	-1.33	-1.72	-1.27	-1.23	2
Protein MCM10 homolog	1.23	1.10	1.09	-1.10	-1.52	-1.58	-1.26	-1.28	2
Protein MIS12 homolog	1.34	1.26	1.63	1.14	-1.27	-1.21	-1.10	-1.25	2
Protein MRVI1	-1.08	-1.12	-1.39	-1.67	-2.08	-2.28	-1.70	-1.22	2
Protein NPAT	-1.04	-1.16	-1.06	-1.24	-1.31	-1.27	-1.20	-1.13	2
Protein phosphatase 1 regulatory subunit 3C-B	1.11	1.36	1.65	-1.05	-1.25	-1.24	-1.06	1.28	2
Protein RRP5 homolog	1.06	1.10	1.07	-1.20	-1.32	-1.89	-1.16	-1.24	2
Protein S100-A1	1.22	1.18	1.06	1.02	1.14	-1.05	-1.61	-1.29	2
Protein S100-G	1.04	1.15	-1.00	-1.09	-1.03	-1.17	-1.68	-1.23	2
Protein SCO1 homolog. mitochondrial	1.02	1.27	1.22	1.17	1.01	-1.10	-1.14	-1.10	2
Protein SDA1 homolog	1.01	1.10	1.00	-1.28	-1.55	-2.05	-1.57	-1.38	2
Protein slowmo homolog 2	-1.27	1.25	-1.39	-1.87	-2.40	-3.62	-1.97	-1.59	2
Protein TBRG4	-1.05	1.19	1.11	1.01	-1.12	-1.28	-1.17	-1.16	2
Protein transport protein Sec23A	1.29	1.50	1.44	1.32	1.33	-1.16	-1.15	-1.23	2
Protein transport protein Sec23B	1.05	1.28	1.24	1.20	1.20	-1.56	-1.24	-1.15	2
Protein VPRBP	1.24	1.11	-1.15	-1.32	-1.35	-1.36	-1.23	-1.01	2
Protein zwilch homolog	1.23	1.26	1.53	1.42	-1.10	-1.03	-1.04	-1.17	2
Protein-glutamine gamma-glutamyltransferase 2	1.16	1.22	1.41	-1.18	-1.45	-3.13	-2.91	-2.00	2
Proto-oncogene c-Fos-like	-1.45	1.70	-1.26	-1.43	-1.40	-2.37	-2.19	-1.74	2
Pumilio domain-containing protein C14orf21	1.07	-1.06	-1.39	-1.86	-2.00	-2.41	-1.55	-1.33	2
Pumilio domain-containing protein KIAA0020	-1.14	-1.08	-1.06	-1.26	-1.38	-2.07	-1.35	-1.17	2
Pupal cuticle protein G1A. putative	1.04	-1.10	-1.27	-1.11	1.01	-1.31	-2.46	-1.29	2
Putative all-trans-retinol 13.14-reductase	-1.03	-1.05	-1.10	-1.21	-1.27	-1.26	-1.28	-1.23	2
Putative ATP-dependent RNA helicase DHX30	1.05	1.05	-1.22	-1.29	-1.06	-1.56	-1.22	-1.19	2
Putative ATP-dependent RNA helicase DHX57	-1.11	1.18	1.17	1.03	1.07	-1.32	-1.11	-1.20	2
Putative pre-mRNA-splicing factor ATP-dependent RNA helicase PRP1	1.20	4.64	4.22	2.66	3.63	-1.25	1.00	1.29	2
Putative ribosomal RNA methyltransferase 1	-1.14	-1.22	-1.24	-1.30	-1.35	-1.35	-1.23	-1.13	2
Putative ribosomal RNA methyltransferase NOP2	-1.14	-1.18	-1.09	-1.30	-1.69	-2.10	-1.51	-1.37	2
Putative rRNA methyltransferase 3	-1.17	-1.26	-1.15	-1.41	-1.84	-2.40	-1.63	-1.42	2
Putative transferase CAF17 homolog. mitochondrial	-1.06	-1.08	-1.16	-1.31	-1.28	-1.30	-1.12	-1.14	2
Ran guanine nucleotide release factor	1.02	-1.05	1.04	-1.38	-1.28	-1.50	-1.40	-1.24	2
Rap guanine nucleotide exchange factor 4	1.24	1.29	-1.29	-1.39	-1.65	-2.06	-1.23	1.07	2
Ras-GEF domain-containing family member 1B-A	-1.33	-1.11	-1.19	-1.58	-2.24	-2.33	-2.20	-1.32	2
RecQ-mediated genome instability protein 2	1.09	1.41	1.29	1.05	-1.42	-1.63	-1.23	-1.32	2
Regulator of telomere elongation helicase 1	1.03	-1.01	1.15	1.20	-1.11	-1.22	-1.16	-1.28	2
Replication factor C subunit 3	1.18	1.24	1.31	1.09	-1.33	-1.36	-1.19	-1.21	2
Replication factor C subunit 5	-1.18	-1.28	-1.54	-2.15	-2.82	-1.98	-1.42	-1.26	2
Replication protein A 32 kDa subunit	1.11	1.14	1.22	1.12	-1.48	-1.46	-1.21	-1.22	2
Replication protein A 70 kDa DNA-binding subunit	1.23	1.43	1.59	1.39	-1.30	-1.55	-1.28	-1.16	2
Response gene to complement 32 protein	-1.03	-1.11	-1.67	-1.69	-1.97	-1.76	-1.83	-1.50	2
REST corepressor 1	1.13	1.05	1.02	-1.10	-1.19	-1.27	-1.18	-1.12	2
Retinal cone rhodopsin-sensitive cGMP 3'.5'-cyclic phosphodiesterase subunit gamma	1.30	1.75	1.77	-1.01	-1.53	-2.47	-7.73	-1.99	2

Gene Name	Fold-Change								Cluster
	00/24	03/24	06/24	09/24	12/24	15/24	18/21	21/24	
Retinal rod rhodopsin-sensitive cGMP 3'.5'-cyclic phosphodiesterase subunit gamma-like	1.22	1.48	1.24	-1.15	-1.38	-1.71	-2.85	-1.12	2
Retinoblastoma-associated protein	1.14	1.04	-1.10	-1.55	-1.71	-1.51	-1.21	-1.05	2
Retinoic acid receptor responder protein 3-like	1.04	-1.01	-1.66	-1.26	-1.27	-1.77	-1.85	-1.15	2
Rhamnose-binding lectin	1.35	1.15	-1.18	1.15	1.03	-1.23	-2.61	-1.32	2
Rho GTPase-activating protein 15	-1.27	-1.30	-1.45	-1.84	-2.16	-1.91	-2.00	-1.37	2
Rho-related GTP-binding protein RhoN	1.08	1.09	-1.16	-1.27	-1.35	-1.46	-1.20	-1.05	2
Riboflavin kinase	-1.04	1.36	-1.12	-1.48	-1.59	-1.90	-1.34	-1.03	2
Riboflavin transporter 2-A	1.09	2.12	2.14	1.54	1.12	-1.75	-2.02	-2.01	2
Ribonuclease H2 subunit A	1.03	1.04	1.31	1.14	-1.20	-1.22	-1.31	-1.20	2
Ribonucleases P/MRP protein subunit POP1	-1.01	1.00	-1.18	-1.44	-1.76	-1.81	-1.37	-1.42	2
Ribonucleoside-diphosphate reductase subunit M2	1.47	2.07	2.02	1.57	1.18	1.17	-1.05	-1.29	2
Ribonucleoside-diphosphate reductase subunit M2 B	-1.33	1.09	-1.48	-1.69	-1.31	-1.48	-1.35	-1.52	2
Ribose-phosphate pyrophosphokinase 1	1.16	1.03	1.11	1.03	-1.17	-1.11	-1.03	1.01	2
Ribose-phosphate pyrophosphokinase 2	1.02	1.17	-1.06	-1.11	1.00	-1.41	-1.46	-1.11	2
Ribosomal L1 domain-containing protein 1	-1.26	-1.29	-1.29	-1.54	-2.02	-2.77	-1.89	-1.34	2
Ribosomal protein 63. mitochondrial	-1.10	-1.11	-1.10	-1.12	-1.27	-1.33	-1.24	-1.15	2
Ribosomal RNA processing protein 1 homolog A	-1.12	-1.09	1.00	-1.21	-1.44	-1.95	-1.39	-1.31	2
Ribosomal RNA processing protein 1 homolog B	-1.08	-1.10	1.01	-1.24	-1.49	-1.85	-1.39	-1.23	2
Ribosomal RNA small subunit methyltransferase NEP1	-1.11	-1.23	-1.27	-1.55	-1.86	-2.01	-1.54	-1.34	2
Ribosomal RNA-processing protein 7 homolog A	-1.02	-1.08	-1.09	-1.24	-1.56	-1.69	-1.34	-1.26	2
Ribosome biogenesis protein BMS1 homolog	1.04	1.11	1.10	1.09	-1.10	-1.29	-1.13	-1.10	2
Ribosome biogenesis protein bop1	-1.01	1.10	1.16	-1.07	-1.30	-1.56	-1.44	-1.24	2
Ribosome biogenesis protein BRX1 homolog	1.21	1.08	1.09	-1.18	-1.53	-1.84	-1.18	-1.27	2
Ribosome biogenesis protein wdr12	-1.06	1.15	1.07	-1.22	-1.26	-1.80	-1.27	-1.32	2
Ribosome production factor 1	-1.01	-1.14	-1.10	-1.35	-1.54	-1.75	-1.33	-1.23	2
RuvB-like 1	-1.02	-1.03	1.18	1.23	-1.02	-1.13	-1.12	-1.14	2
S-adenosylmethionine synthase isoform type-2	1.24	1.25	1.40	-1.13	-1.57	-2.39	-1.57	-1.38	2
Scavenger receptor class B member 1	1.01	1.16	-1.01	-1.11	-1.40	-1.60	-1.15	-1.16	2
Sel1 repeat-containing protein 1	1.03	-1.03	-1.16	-1.32	-1.48	-1.39	-1.10	-1.12	2
Ser/Thr-rich protein T10 in DGCR region	-1.00	1.05	-1.21	-1.36	-1.42	-1.58	-1.26	-1.17	2
Serine hydroxymethyltransferase. cytosolic	1.19	1.46	1.42	1.39	1.23	1.03	-1.00	-1.14	2
Serine incorporator 2	1.31	1.56	2.05	-1.33	1.43	1.16	-1.39	1.12	2
Serine/threonine-protein kinase 35	-1.16	-1.22	-1.24	-1.46	-2.18	-2.93	-2.54	-1.97	2
Serine/threonine-protein kinase Chk1	1.30	1.24	1.42	1.01	-1.79	-1.84	-1.70	-1.35	2
Serine/threonine-protein kinase RIO1	-1.00	-1.00	-1.08	-1.37	-1.43	-1.93	-1.32	-1.14	2
Serine/threonine-protein kinase RIO2	-1.12	-1.18	-1.31	-1.47	-1.64	-1.83	-1.28	-1.20	2
Serine/threonine-protein kinase SBK2	-1.25	1.26	1.10	-1.15	-1.99	-2.93	-2.85	-1.79	2
Serine/threonine-protein kinase Sgk1	-1.31	1.01	-1.29	-1.56	-2.53	-3.64	-3.74	-2.16	2
Serine/threonine-protein kinase Sgk1-A	-1.03	1.64	-1.08	-1.61	-2.21	-3.37	-2.85	-1.23	2
Serine/threonine-protein kinase ULK4	1.19	1.41	1.45	1.46	1.15	1.01	1.08	1.02	2
Serine-rich adhesin for platelets-like	-1.04	1.37	-1.61	-1.95	-1.68	-2.29	-1.45	-1.37	2
Serum response factor-binding protein 1	-1.05	-1.12	-1.04	-1.13	-1.19	-1.30	-1.24	-1.26	2

Gene Name	Fold-Change								Cluster
	00/24	03/24	06/24	09/24	12/24	15/24	18/21	21/24	
Short-chain dehydrogenase/reductase 3	-1.13	-1.17	-1.09	-1.28	-1.53	-1.78	-1.50	-1.48	2
Sister chromatid cohesion protein DCC1	1.35	1.40	1.78	1.39	-1.10	-1.29	-1.14	-1.20	2
Small glutamine-rich tetratricopeptide repeat-containing protein beta	-1.04	1.07	-1.35	-1.59	-1.45	-1.46	-1.22	-1.04	2
Sodium- and chloride-dependent taurine transporter	1.09	1.18	1.03	-1.14	-1.34	-1.57	-1.58	1.01	2
Sodium-coupled neutral amino acid transporter 3	1.04	1.19	1.10	-1.12	-1.45	-1.74	-1.78	-1.57	2
Sodium-coupled neutral amino acid transporter 5	-1.00	-1.18	-1.23	-1.67	-2.13	-1.63	-1.03	-1.25	2
Sodium-dependent phosphate transporter 1-B	-1.28	-1.13	-1.27	-1.78	-2.37	-2.80	-2.27	-1.43	2
Soluble lamin-associated protein of 75 kDa	-1.43	-1.59	-1.75	-1.84	-2.98	-3.54	-3.13	-2.09	2
Solute carrier family 25 member 34	-1.07	-1.08	-1.41	-1.53	-1.42	-1.50	-1.34	-1.25	2
Solute carrier family 25 member 38-A	-1.01	1.01	-1.19	-1.59	-1.59	-2.45	-2.21	-1.46	2
Solute carrier family 25 member 38-B	1.01	1.05	-1.21	-1.85	-1.86	-2.77	-2.66	-1.44	2
Solute carrier family 25 member 40	1.03	1.03	-1.13	-1.47	-1.61	-1.65	-1.36	-1.25	2
Solute carrier family 41 member 2	-1.01	1.02	-1.10	-1.23	-1.32	-1.25	-1.39	-1.04	2
Solute carrier organic anion transporter family member 2A1	1.04	1.05	-1.53	-1.85	-2.02	-2.03	-1.45	-1.23	2
Sororin-B	1.12	1.14	1.56	1.40	1.03	1.10	-1.07	-1.14	2
Speckle targeted PIP5K1A-regulated poly(A) polymerase-like	1.07	-1.00	1.14	1.05	-1.18	-1.37	-1.31	-1.16	2
Sperm-associated antigen 1	1.10	1.17	-1.20	-1.59	-1.61	-1.67	-1.15	-1.14	2
Spermatogenesis-associated protein 5-like	1.07	1.40	1.45	1.19	-1.08	-1.40	-1.21	-1.31	2
Sphingolipid delta(4)-desaturase DES1	-1.01	1.32	1.33	1.28	1.10	-1.03	-1.12	-1.17	2
SPRY domain-containing protein 4	-1.01	-1.11	-1.29	-1.45	-1.59	-1.51	-1.16	-1.12	2
Stabilin-2	-1.33	1.00	1.02	-1.16	-1.44	-1.59	1.13	1.11	2
Sterol 26-hydroxylase. mitochondrial	1.14	1.28	1.16	-1.09	-1.59	-1.68	-1.51	-1.01	2
Stomatin-like protein 2	-1.02	1.15	1.21	1.03	-1.05	-1.21	-1.05	-1.31	2
Succinate dehydrogenase [ubiquinone] iron-sulfur subunit. mitochondrial	-1.28	1.29	-1.06	-1.34	-1.85	-3.41	-3.45	-2.31	2
Succinate dehydrogenase assembly factor 2. mitochondrial	1.04	1.32	1.04	-1.10	-1.32	-1.46	-1.44	-1.41	2
Sulfite oxidase. mitochondrial	1.03	1.30	-1.12	-1.19	-1.16	-1.23	1.08	1.00	2
Sulfotransferase 1C1	-1.01	1.26	1.14	1.12	-1.02	-1.41	-1.24	-1.43	2
Sulfotransferase family cytosolic 2B member 1	-1.06	1.15	1.25	-1.06	-1.26	-1.36	-1.65	-1.55	2
Sushi domain-containing protein 3	1.20	1.08	-1.21	-1.44	-1.43	-1.52	-1.04	1.11	2
Swi5-dependent recombination DNA repair protein 1 homolog	-1.18	-1.24	-1.16	-1.39	-1.63	-1.44	-1.44	-1.01	2
Synemin-like	-1.01	-1.11	-1.48	-1.91	-2.06	-2.06	-1.65	-1.05	2
Syntabulin-like	1.05	1.27	-1.23	-1.89	-1.86	-2.02	-1.63	-1.00	2
Syntaxin-binding protein 6	-1.16	1.05	-1.09	-1.37	-1.47	-1.45	-1.15	-1.11	2
T-cell activation Rho GTPase-activating protein	1.09	1.10	-1.15	-1.37	-1.53	-1.83	-1.59	-1.02	2
Tctex1 domain-containing protein 1-B	-1.06	1.16	1.10	-1.02	-1.10	-1.36	-1.27	-1.27	2
Telomere-associated protein RIF1	1.14	1.32	1.17	1.00	-1.32	-1.25	-1.02	-1.00	2
Tetratricopeptide repeat protein 22-like	-1.02	-1.08	-1.13	-1.50	-1.66	-1.51	-1.62	-1.05	2
Tetratricopeptide repeat protein 27	1.07	1.22	1.38	1.20	1.08	-1.14	-1.05	-1.17	2
Tetratricopeptide repeat protein 36	-1.04	1.10	1.14	1.18	-1.07	-1.16	-1.29	-1.35	2
Tetratricopeptide repeat protein 4	-1.14	-1.12	-1.11	-1.19	-1.27	-1.50	-1.29	-1.12	2

Gene Name	Fold-Change								Cluster
	00/24	03/24	06/24	09/24	12/24	15/24	18/21	21/24	
Thioesterase superfamily member 4	-1.07	1.06	-1.21	-1.35	-1.46	-1.51	-1.14	-1.11	2
Thioredoxin reductase 3	-1.05	1.13	1.25	1.28	-1.04	-1.17	-1.10	-1.20	2
Thymidine kinase. cytosolic	1.22	1.36	1.88	1.62	-1.03	1.26	1.00	-1.27	2
Thymidylate synthase	1.34	1.32	1.22	-1.23	-1.74	-1.85	-1.46	-1.23	2
Thymocyte nuclear protein 1	1.02	1.01	1.11	-1.13	-1.36	-1.28	-1.24	-1.12	2
TIMELESS-interacting protein	1.30	1.33	1.53	1.08	-1.19	-1.39	1.01	-1.07	2
Transcription factor E2F8	1.03	1.07	1.36	1.20	-1.00	-1.19	-1.13	-1.17	2
Transducin beta-like protein 3	-1.03	-1.01	1.17	-1.12	-1.40	-1.93	-1.35	-1.22	2
Translocator protein	1.00	1.36	1.07	-1.06	-1.15	-1.46	-1.24	-1.29	2
Transmembrane 4 L6 family member 4	1.10	-1.14	-1.20	-1.86	-1.74	-1.90	-1.74	-1.25	2
Transmembrane protease serine 2	1.05	1.22	-1.12	-1.68	-1.66	-1.49	-1.08	-1.15	2
Transmembrane protein 11. mitochondrial	-1.06	-1.02	-1.18	-1.28	-1.36	-1.38	-1.32	-1.08	2
Transmembrane protein 120A	-1.19	1.03	-1.08	-1.06	-1.32	-1.45	-1.36	-1.27	2
Transmembrane protein 128	-1.11	-1.14	-1.08	-1.23	-1.24	-1.48	-1.22	-1.06	2
Transmembrane protein 231	1.05	1.05	1.21	1.16	1.06	-1.05	-1.20	-1.16	2
Transmembrane protein 236	1.04	1.06	-1.39	-1.69	-2.15	-1.82	-1.36	-1.14	2
Transmembrane protein adipocyte-associated 1 homolog	1.22	1.13	-1.19	-1.35	-1.30	-1.36	-1.32	-1.04	2
Tripartite motif-containing protein 16-like protein	1.04	1.34	1.34	1.32	1.35	-1.20	-1.13	-1.06	2
TRNA (adenine-N(1)-)-methyltransferase non-catalytic subunit TRM6	1.09	-1.01	1.13	-1.13	-1.27	-1.57	-1.30	-1.32	2
TRNA (cytosine(34)-C(5))-methyltransferase	-1.02	-1.10	1.05	1.03	-1.31	-1.37	-1.17	-1.20	2
TRNA (guanine-N(7)-)-methyltransferase subunit WDR4	1.06	-1.00	1.00	-1.28	-1.50	-1.79	-1.33	-1.17	2
TRNA-dihydrouridine synthase 4-like	-1.23	-1.13	-1.10	-1.37	-1.44	-1.66	-1.18	-1.17	2
Trypsin	1.05	1.45	1.58	1.40	1.62	1.25	-2.40	-1.43	2
Trypsin-1	1.13	-1.01	-1.36	-2.09	-1.33	-1.19	-2.28	-1.09	2
Tsukushin	-1.30	1.42	-1.15	-1.49	-2.11	-1.94	-1.24	-1.16	2
Tubulin delta chain	1.06	1.12	1.30	1.12	1.15	-1.09	-1.06	-1.03	2
Tubulin--tyrosine ligase-like protein 12	1.10	1.16	1.01	-1.13	-1.16	-1.51	-1.02	-1.16	2
Tuftelin	1.07	1.18	-1.29	-2.34	-2.05	-3.17	-2.77	-1.74	2
Tyrosine aminotransferase	-1.08	1.43	-1.08	-1.97	-3.83	-7.31	-4.08	-3.04	2
Tyrosine-protein phosphatase non-receptor type 22	1.07	-1.21	-1.17	-1.43	-1.51	-1.37	-1.61	1.14	2
U2 small nuclear ribonucleoprotein A'	-1.08	1.07	1.19	1.23	1.01	-1.31	-1.17	-1.13	2
U3 small nucleolar ribonucleoprotein protein IMP3	-1.04	-1.12	-1.03	-1.12	-1.23	-1.39	-1.29	-1.28	2
U3 small nucleolar ribonucleoprotein protein IMP4	-1.02	-1.20	-1.23	-1.41	-1.70	-1.72	-1.44	-1.26	2
U3 small nucleolar RNA-associated protein 15 homolog	1.12	1.10	1.19	-1.07	-1.21	-1.81	-1.27	-1.29	2
U3 small nucleolar RNA-associated protein 6 homolog	1.09	1.08	1.06	-1.22	-1.56	-1.85	-1.29	-1.27	2
U3 small nucleolar RNA-interacting protein 2	-1.01	-1.17	-1.18	-1.39	-1.51	-1.68	-1.31	-1.17	2
U4/U6 small nuclear ribonucleoprotein Prp3	1.09	1.21	1.12	1.05	1.00	-1.16	-1.07	-1.07	2
Ubiquitin carboxyl-terminal hydrolase 9	1.04	1.29	1.19	1.01	-1.15	-1.69	-1.76	-1.58	2
Ubiquitin-conjugating enzyme E2 T	1.12	1.28	1.51	1.29	-1.17	-1.11	-1.35	-1.24	2
UBX domain-containing protein 2A	1.03	1.01	1.01	-1.01	-1.53	-1.38	1.14	1.00	2

Gene Name	Fold-Change								Cluster
	00/24	03/24	06/24	09/24	12/24	15/24	18/21	21/24	
Uncharacterized methyltransferase WBSCR22	-1.01	1.02	1.02	-1.17	-1.38	-1.49	-1.22	-1.20	2
Uncharacterized oxidoreductase C663.06c	1.10	1.15	-1.05	1.25	-1.33	-1.39	1.10	-1.19	2
Uncharacterized protein At5g50100. mitochondrial	-1.10	1.28	1.04	-1.48	-1.96	-2.49	-2.39	-1.73	2
Uncharacterized protein C15orf29	-1.07	-1.08	-1.40	-1.76	-1.54	-1.39	-1.35	-1.17	2
Uncharacterized protein C1orf50 homolog	-1.23	1.10	1.05	-1.42	-1.68	-1.80	-1.65	-1.48	2
Uncharacterized protein C2orf71-like	1.31	1.23	-1.02	-1.59	-1.00	-1.30	-2.11	-1.26	2
Uncharacterized protein C3orf17 homolog	-1.06	-1.41	-1.54	-2.09	-1.86	-1.96	-1.55	-1.38	2
Uncharacterized protein C3orf26 homolog	1.09	1.03	1.08	-1.02	-1.36	-1.55	-1.33	-1.20	2
Uncharacterized protein C4orf14 homolog	1.04	-1.08	1.06	-1.16	-1.25	-1.54	-1.29	-1.25	2
Uncharacterized protein C5orf34 homolog	1.13	1.36	1.43	1.43	-1.23	-1.23	-1.07	-1.33	2
Uncharacterized protein C5orf4	1.12	1.18	1.02	-1.35	-1.16	-1.63	-1.63	-1.23	2
Uncharacterized protein DKFZp761E198	1.05	1.01	-1.10	-1.39	-1.25	-1.32	-1.38	-1.15	2
Uncharacterized protein LOC101156258	1.27	-1.32	-1.49	-1.40	-1.33	-1.25	-5.52	-1.88	2
Uncharacterized protein LOC101156901	-1.02	1.67	-1.07	-1.26	-1.14	-1.59	-1.79	-1.13	2
Uncharacterized protein LOC101158054	1.74	-1.14	-1.49	1.08	-1.66	-1.04	-5.50	-1.53	2
Uncharacterized protein LOC101166720	1.28	2.27	1.44	-1.15	-1.30	-1.80	-1.74	1.04	2
Uncharacterized protein LOC101166828	1.64	-1.07	-1.07	-1.01	-1.04	1.05	-4.95	-1.52	2
Uncharacterized protein LOC101171617	1.49	-1.01	-1.07	1.10	1.01	-1.05	-3.37	-1.22	2
Uncharacterized protein LOC101474366 isoform X1	1.42	1.92	1.71	1.44	1.13	-1.05	-1.25	-1.24	2
Uncharacterized protein LOC101482961	-1.15	-1.45	-1.88	-1.80	-2.89	-2.77	-2.02	-1.38	2
Uncharacterized protein LOC101484993	1.74	-1.02	1.02	1.08	1.06	1.11	-4.18	-1.39	2
Uncharacterized protein MJ0240	1.21	1.45	1.63	1.27	1.00	-1.10	-1.12	-1.19	2
Uncharacterized protein SYNPCC7002_A1628	1.01	1.02	-1.30	-1.39	-1.49	-1.33	-1.27	-1.11	2
Unconventional myosin-Vb	1.24	1.32	1.06	-1.27	-1.04	-1.31	-1.12	1.12	2
Unhealthy ribosome biogenesis protein 2 homolog	1.09	-1.02	-1.09	-1.34	-1.44	-1.61	-1.25	-1.12	2
UPF0498 protein KIAA1191	1.03	1.09	-1.06	-1.23	-1.22	-1.33	-1.21	-1.10	2
UPF0498 protein KIAA1191 homolog	1.14	1.01	-1.12	-1.38	-1.61	-1.70	-1.31	-1.17	2
Uracil-DNA glycosylase	-1.11	-1.24	-1.40	-1.71	-1.81	-1.68	-1.25	-1.17	2
Urea transporter 2	1.03	1.15	-1.10	-2.10	-1.83	-2.25	-1.38	-1.31	2
Vinexin	1.16	-1.00	-1.51	-1.65	-2.12	-1.75	-1.35	-1.09	2
V-set and transmembrane domain-containing protein 5	-1.12	-1.16	-1.32	-1.76	-1.65	-1.57	-1.41	-1.09	2
WD repeat and HMG-box DNA-binding protein 1	1.43	1.49	1.68	1.29	-1.44	-1.57	-1.30	-1.27	2
WD repeat and SOCS box-containing protein 2	1.03	1.13	-1.10	-1.51	-1.62	-2.01	-1.48	-1.24	2
WD repeat-containing protein 36	-1.17	-1.16	-1.06	-1.33	-1.50	-2.06	-1.44	-1.33	2
WD repeat-containing protein 43	-1.70	-1.29	-1.22	-1.50	-1.39	-2.43	-2.16	-1.44	2
WD repeat-containing protein 46	1.03	-1.00	1.09	-1.27	-1.39	-1.67	-1.37	-1.16	2
WD repeat-containing protein 53	-1.00	-1.08	-1.19	-1.63	-1.81	-2.15	-1.78	-1.49	2
WD repeat-containing protein 55	-1.04	-1.17	-1.17	-1.21	-1.46	-2.02	-1.40	-1.28	2
WD repeat-containing protein 75	-1.10	-1.08	-1.25	-1.24	-1.32	-1.86	-1.25	-1.20	2
Zinc finger C3H1 domain-containing protein	1.06	1.20	1.32	1.17	1.13	-1.08	-1.20	-1.00	2
Zinc finger CCHC domain-containing protein 4	-1.10	-1.20	-1.02	-1.28	-1.74	-2.02	-1.39	-1.30	2
Zinc finger CCHC domain-containing protein 9	-1.04	-1.05	-1.09	-1.12	-1.28	-1.34	-1.19	-1.10	2

Gene Name	Fold-Change								Cluster
	00/24	03/24	06/24	09/24	12/24	15/24	18/21	21/24	
Zinc finger protein 36. C3H1 type-like 1	-1.02	-1.19	-1.30	-1.33	-1.48	-1.40	-1.41	-1.22	2
Zinc finger protein 598	1.12	-1.01	-1.29	-1.69	-1.93	-2.12	-1.80	-1.41	2
Zinc finger protein 704	1.06	1.10	-1.14	-1.61	-2.14	-1.67	-1.55	-1.19	2
Zinc finger protein 81	1.05	1.10	-1.06	-1.05	1.05	-1.30	-1.10	-1.15	2
Zinc finger protein ZPR1	-1.19	-1.10	1.05	-1.13	-1.19	-1.76	-1.36	-1.36	2
[Pyruvate dehydrogenase [acetyl-transferring]]-phosphatase 1. mitochondrial	-1.11	1.65	2.36	2.76	1.64	1.23	1.28	1.26	3
[Pyruvate dehydrogenase [acetyl-transferring]]-phosphatase 2. mitochondrial	1.21	1.31	1.52	1.45	1.53	1.29	1.26	-1.01	3
14-3-3 protein beta/alpha-1	1.02	-1.02	1.07	1.12	1.10	1.19	1.13	1.03	3
26S protease regulatory subunit 4	1.22	1.23	1.29	1.35	1.34	1.54	1.44	1.19	3
26S protease regulatory subunit 7	1.03	1.04	1.10	1.26	1.21	1.29	1.49	1.07	3
26S protease regulatory subunit 8	1.05	1.15	1.25	1.23	1.21	1.29	1.45	1.06	3
26S proteasome non-ATPase regulatory subunit 1	1.33	1.37	1.41	1.49	1.55	1.63	1.70	1.17	3
26S proteasome non-ATPase regulatory subunit 3	1.09	1.14	1.13	1.16	1.27	1.29	1.33	1.11	3
26S proteasome non-ATPase regulatory subunit 4	1.01	1.02	1.06	1.08	1.09	1.23	1.24	1.07	3
26S proteasome non-ATPase regulatory subunit 7	-1.02	1.01	1.19	1.31	1.20	1.23	1.32	-1.00	3
26S proteasome non-ATPase regulatory subunit 8	-1.03	1.04	1.17	1.20	1.19	1.28	1.47	1.12	3
2-hydroxyacylsphingosine 1-beta-galactosyltransferase	1.24	1.49	1.50	2.04	2.08	2.26	1.90	1.48	3
3-keto-steroid reductase	1.29	1.85	2.22	2.31	1.70	1.26	-1.10	-1.33	3
5'-AMP-activated protein kinase subunit gamma-3	-1.13	-1.32	1.09	1.98	1.30	2.27	2.04	1.02	3
6-phosphofructo-2-kinase/fructose-2.6-biphosphatase 2	-1.07	1.28	1.56	1.94	2.00	1.50	1.46	1.01	3
6-phosphofructo-2-kinase/fructose-2.6-biphosphatase 3	-1.10	1.22	1.30	2.38	1.96	-1.06	1.66	1.10	3
6-phosphofructokinase. liver type	-1.02	1.17	1.21	1.36	1.40	1.29	1.09	-1.04	3
78 kDa glucose-regulated protein	-1.31	1.07	1.43	2.11	1.79	1.45	1.45	-1.09	3
A disintegrin and metalloproteinase with thrombospondin motifs 1	1.16	1.02	1.03	1.35	2.08	1.52	1.56	1.07	3
A disintegrin and metalloproteinase with thrombospondin motifs 17	1.22	1.22	2.21	3.22	2.64	3.19	2.38	1.44	3
Abhydrolase domain-containing protein 10. mitochondrial	1.05	1.05	1.21	1.28	1.23	1.11	1.08	-1.01	3
Abnormal spindle-like microcephaly-associated protein homolog	1.01	-1.18	-1.04	1.57	1.71	3.39	2.16	1.51	3
Acidic repeat-containing protein	1.16	1.14	1.43	1.83	1.72	2.63	1.54	1.03	3
Actin	1.10	1.12	1.22	1.27	1.34	1.30	1.24	1.07	3
Actin filament-associated protein 1-like 1	-1.06	-1.14	1.02	1.07	1.12	1.36	1.24	1.19	3
Actin. clone 403	1.10	1.17	1.17	1.26	1.39	1.27	1.31	1.11	3
Actin-binding protein anillin	1.09	1.23	1.95	2.51	2.31	3.71	2.01	1.41	3
Activity-dependent neuroprotector homeobox protein	1.01	-1.02	1.22	1.17	1.11	1.08	1.08	1.03	3
Acylamino-acid-releasing enzyme	1.02	-1.05	1.02	1.04	1.03	1.22	1.10	1.04	3
Acyl-coenzyme A thioesterase 4	-1.04	1.05	1.17	1.34	1.17	1.76	1.51	1.11	3
Acyl-protein thioesterase 2	1.01	-1.13	1.09	1.25	1.15	1.33	1.15	1.07	3

Gene Name	Fold-Change								Cluster
	00/24	03/24	06/24	09/24	12/24	15/24	18/21	21/24	
Adenosine 3'-phospho 5'-phosphosulfate transporter 2	-1.07	-1.02	1.14	1.21	1.22	1.17	1.07	-1.06	3
Adenylyltransferase and sulfurtransferase MOCS3	-1.10	-1.03	1.14	1.34	1.27	1.22	1.28	-1.04	3
ADP-ribosylation factor 1	-1.20	-1.19	1.06	1.13	1.08	1.10	1.06	-1.04	3
ADP-ribosylation factor-like protein 3	1.12	1.03	1.22	1.26	1.33	1.24	1.09	1.00	3
Alanine aminotransferase 2-like	1.03	1.24	1.11	1.46	1.41	1.28	1.37	1.00	3
Aldehyde dehydrogenase family 9 member A1	-1.10	1.35	2.13	2.34	2.28	1.84	1.43	1.04	3
Aldehyde dehydrogenase family 9 member A1-B	1.06	1.09	1.26	1.51	1.47	1.72	1.59	1.11	3
Aldo-keto reductase family 1 member B15	1.18	1.23	1.55	1.83	1.73	1.58	1.44	1.00	3
Alpha-1.2-mannosyltransferase ALG9	1.08	1.17	1.27	1.36	1.41	1.26	1.34	1.10	3
Alpha-1-inhibitor 3	1.02	1.41	1.68	1.53	1.90	1.35	1.05	-1.06	3
Alpha-actinin-1	1.19	1.20	1.35	1.69	1.56	1.13	1.06	1.07	3
Alpha-mannosidase 2C1	1.36	1.20	1.27	1.41	1.47	1.64	1.63	1.37	3
Amiloride-sensitive amine oxidase [copper-containing]	1.27	1.11	1.07	1.35	1.54	1.80	1.55	1.33	3
Amine oxidase [flavin-containing] A	1.17	1.64	1.77	2.56	2.65	2.75	2.48	1.74	3
AMP deaminase 3	-1.05	1.03	1.02	1.38	1.84	2.20	1.56	1.44	3
Angiopoietin-related protein 6	1.24	1.03	1.32	1.37	1.55	1.66	1.83	1.27	3
Ankycorbin	-1.23	-1.24	1.33	1.77	1.96	2.71	1.85	1.25	3
Ankyrin repeat and SOCS box protein 4	-1.06	1.04	1.21	1.62	1.74	1.88	2.27	1.60	3
Ankyrin repeat and SOCS box protein 5	-1.26	-1.55	-1.25	1.38	1.92	2.49	1.61	1.29	3
Ankyrin repeat domain-containing protein 10	-1.08	-1.01	1.26	1.38	1.39	1.22	1.05	1.09	3
Ankyrin repeat domain-containing protein 13C	1.12	1.04	-1.03	1.03	1.03	1.14	-1.07	-1.03	3
Ankyrin repeat domain-containing protein 50	-1.10	-1.06	1.04	1.36	1.42	1.53	1.17	1.07	3
Antigen KI-67	-1.10	-1.09	1.46	1.91	1.56	2.25	1.12	1.14	3
AP-4 complex subunit sigma-1	1.14	1.19	1.28	1.67	1.46	1.69	1.55	1.40	3
Apolipoprotein B-100	-1.03	-1.07	1.14	1.95	1.55	1.71	1.30	1.07	3
Apoptosis facilitator Bcl-2-like protein 14	-1.04	1.69	2.43	3.12	2.49	2.19	1.33	-1.26	3
Arachidonate 12-lipoxygenase. 12S-type	1.15	1.08	1.36	1.83	1.98	2.09	1.50	1.31	3
Arfaptin-1	1.03	-1.00	1.10	1.23	1.23	1.26	1.10	1.02	3
Arginine/serine-rich coiled-coil protein 2	-1.23	2.63	1.13	1.41	1.31	1.51	1.11	1.61	3
Aryl hydrocarbon receptor nuclear translocator-like protein 1 (BMAL1)	-2.09	2.00	2.81	3.95	3.64	2.18	1.42	-1.24	3
Arylsulfatase K	-1.09	-1.00	1.13	1.26	1.22	1.27	1.29	-1.03	3
Asparagine synthetase [glutamine-hydrolyzing]	1.04	1.28	1.46	1.43	1.25	1.06	1.14	-1.14	3
Ataxin-3	-1.10	-1.21	-1.08	-1.04	1.03	1.13	-1.06	-1.04	3
ATPase family AAA domain-containing protein 1-B	-1.07	1.11	1.34	1.28	1.09	-1.01	1.05	-1.00	3
ATP-binding cassette sub-family E member 1	1.03	1.11	1.54	1.48	1.18	-1.05	1.15	-1.21	3
ATP-binding cassette sub-family F member 2	-1.13	1.02	1.40	1.42	1.12	-1.01	1.09	-1.07	3
Aurora kinase A-A	1.09	1.14	1.70	2.51	2.60	3.87	2.09	1.30	3
Baculoviral IAP repeat-containing protein 5.2	1.15	1.12	1.70	2.13	2.02	3.07	1.66	1.18	3
Bardet-Biedl syndrome 12 protein	1.00	1.40	1.44	1.59	1.55	1.29	1.15	-1.02	3
Bcl-2-like protein 12	-1.13	1.05	1.20	1.50	1.81	1.49	1.29	-1.27	3
Beta-citryl-glutamate synthase B	-1.18	1.28	1.68	2.08	1.77	1.09	-1.07	-1.14	3

Gene Name	Fold-Change								Cluster
	00/24	03/24	06/24	09/24	12/24	15/24	18/21	21/24	
Bifunctional 3'-phosphoadenosine 5'-phosphosulfate synthase 1	1.09	1.09	1.34	1.31	1.36	1.21	1.14	1.05	3
Bile acid-CoA:amino acid N-acyltransferase	-1.01	1.16	1.48	1.52	1.95	2.08	1.26	1.02	3
Biliverdin reductase A	1.03	1.34	1.41	1.45	1.13	1.27	1.17	-1.15	3
Bleomycin hydrolase	-1.04	-1.16	1.05	1.09	1.06	1.29	1.29	1.00	3
Borealin	1.12	1.16	1.79	2.20	2.11	3.08	1.73	1.19	3
Brain-specific serine protease 4	1.09	1.18	1.54	1.68	1.24	1.85	1.20	1.03	3
BRISC and BRCA1-A complex member 1	1.05	-1.06	1.01	1.15	1.15	1.43	1.33	1.14	3
BTB/POZ domain-containing adapter for CUL3-mediated RhoA degradation protein 3	-1.02	1.39	1.19	1.29	1.41	1.23	1.37	1.02	3
Calcium/calmodulin-dependent protein kinase type II delta chain	1.07	1.44	1.66	1.45	1.84	1.66	1.13	1.39	3
Calcium-binding mitochondrial carrier protein SCaMC-2-B	-1.04	1.41	1.61	1.65	1.77	1.63	1.64	-1.12	3
Calcyclin-binding protein	-1.01	-1.09	1.11	1.19	1.12	1.09	1.03	-1.07	3
Calreticulin	1.06	-1.04	1.31	1.53	1.44	1.54	1.60	1.13	3
Calumenin-B	1.09	-1.03	1.30	1.53	1.47	1.52	1.38	1.09	3
cAMP-dependent protein kinase inhibitor alpha	1.16	1.06	1.26	1.63	1.68	2.14	1.70	1.31	3
CAP-Gly domain-containing linker protein 2	-1.07	-1.15	-1.09	1.13	1.22	1.22	1.03	1.17	3
Carboxy-terminal kinesin 2	1.16	1.22	1.48	2.20	1.83	2.86	1.93	1.25	3
Carcinoembryonic antigen-related cell adhesion molecule 6	-1.73	2.13	-1.80	22.29	2.17	2.97	55.19	10.76	3
Cardiomyopathy-associated protein 5	1.23	1.21	2.09	2.84	2.25	2.69	2.02	1.50	3
Carnitine O-palmitoyltransferase 1. muscle isoform	-1.01	1.06	1.15	1.52	1.29	1.07	-1.04	-1.01	3
Cartilage-associated protein	1.07	1.03	1.28	1.46	1.32	1.25	1.17	1.01	3
Casein kinase II subunit alpha	1.00	-1.00	1.19	1.33	1.19	1.24	1.16	1.01	3
Caspase recruitment domain-containing protein 11	1.17	1.19	1.31	1.74	1.89	1.65	1.31	1.20	3
Caspase-8	1.09	1.26	1.45	1.46	1.40	1.41	1.31	1.04	3
Catalase (CPT1A)	-1.04	1.37	1.31	1.95	1.95	1.75	1.70	-1.06	3
Cathepsin K	-1.03	-1.42	1.07	1.21	1.30	1.84	1.25	1.05	3
Cathepsin O	1.07	1.03	1.12	1.33	1.28	1.75	1.91	1.42	3
C-C motif chemokine 21	1.29	1.27	1.36	1.56	1.86	2.38	2.47	1.80	3
C-C motif chemokine 4	1.31	-1.15	1.07	1.38	1.18	1.80	1.54	1.50	3
CCR4-NOT transcription complex subunit 10	1.10	1.03	1.16	1.07	1.15	1.12	-1.09	-1.11	3
Cdc42 effector protein 3	1.05	1.38	1.14	1.45	1.68	2.09	1.96	1.40	3
CDGSH iron-sulfur domain-containing protein 2A	-1.09	-1.11	1.22	1.30	1.20	1.23	1.36	1.10	3
Cell division cycle protein 20 homolog	1.09	-1.04	1.33	1.97	2.18	4.04	2.25	1.47	3
Cell division cycle protein 23 homolog	1.08	1.14	1.29	1.32	1.30	1.15	1.16	1.03	3
Cell division cycle-associated protein 3	1.16	-1.05	1.28	1.95	2.51	4.69	2.16	1.46	3
Centromere protein F	-1.04	1.13	1.44	2.19	1.71	2.78	1.69	1.26	3
Centromere protein I	1.34	1.35	1.74	2.21	2.14	3.09	2.10	1.37	3
Centromere protein K	1.18	1.12	1.50	1.34	1.01	1.22	-1.01	-1.19	3
Centromere protein N	1.20	1.02	1.75	1.83	1.59	2.09	-1.06	-1.03	3
Centromere protein U	1.14	1.41	1.82	1.72	1.22	1.26	1.02	-1.16	3

Gene Name	Fold-Change								Cluster
	00/24	03/24	06/24	09/24	12/24	15/24	18/21	21/24	
Centromere-associated protein E	-1.08	1.02	1.67	2.64	2.03	3.34	1.78	1.57	3
Centrosomal protein of 135 kDa	1.05	1.20	1.45	1.43	1.31	1.37	1.08	1.04	3
Centrosomal protein of 57 kDa	-1.01	1.01	1.25	1.24	1.07	1.22	1.02	-1.17	3
Centrosomal protein POC5	-1.10	-1.02	1.08	1.48	1.41	1.75	1.25	1.04	3
Centrosome-associated protein 350	1.07	1.10	1.16	1.20	1.24	1.26	1.08	-1.02	3
Chitinase domain-containing protein 1	1.04	-1.15	1.12	1.27	1.07	1.29	1.30	1.06	3
Choline transporter-like protein 4	1.11	1.56	1.36	1.97	2.16	2.70	1.82	1.07	3
Chromatin accessibility complex protein 1	-1.08	-1.14	1.01	1.10	-1.04	1.16	1.08	-1.04	3
Chromobox protein homolog 5	1.07	1.12	1.28	1.42	1.41	1.37	1.24	1.05	3
Chromodomain-helicase-DNA-binding protein 1-like	1.14	1.20	1.33	1.35	1.23	1.31	1.35	1.02	3
Cip1-interacting zinc finger protein	1.00	1.10	1.13	1.14	1.27	1.13	1.12	-1.01	3
Circadian locomoter output cycles protein kaput	-1.33	1.31	1.94	3.08	3.37	2.48	1.37	-1.32	3
Clathrin light chain A	-1.01	-1.05	1.07	1.15	1.10	1.20	1.12	1.03	3
Claudin-15	1.27	1.14	1.24	2.29	2.63	3.66	3.05	1.52	3
Cleavage and polyadenylation specificity factor subunit 1	1.05	1.08	1.33	1.50	1.48	1.38	1.29	1.00	3
Cleavage stimulation factor subunit 1	1.13	1.21	1.33	1.39	1.38	1.19	1.05	1.02	3
Cleavage stimulation factor subunit 2 tau variant	-1.28	-1.00	-1.02	1.33	1.17	1.15	1.55	1.24	3
Cleavage stimulation factor subunit 2-like	1.02	1.15	1.37	1.90	1.57	1.39	1.79	1.59	3
Cleft lip and palate transmembrane protein 1 homolog	-1.07	-1.05	1.22	1.28	1.38	1.30	1.30	1.04	3
Coenzyme Q-binding protein COQ10 homolog B. mitochondrial	-1.21	1.42	2.25	2.91	3.42	2.98	2.05	1.38	3
Cofilin-2	1.02	1.01	1.12	1.18	1.05	1.22	1.19	1.04	3
Coiled-coil domain-containing protein 167	-1.29	-1.20	1.09	1.30	1.33	1.32	1.34	1.03	3
Coiled-coil domain-containing protein 43	-1.03	-1.03	1.40	1.34	1.19	1.09	1.21	-1.04	3
Coiled-coil domain-containing protein 61	1.01	1.09	1.34	1.72	1.53	1.30	1.24	1.01	3
Coiled-coil domain-containing protein 77	1.02	-1.13	1.13	1.41	1.36	1.79	1.30	-1.04	3
Coiled-coil domain-containing protein 84	-1.06	1.05	1.09	1.22	1.23	1.32	1.33	1.06	3
Collagen alpha-1(VIII) chain-like	1.30	1.21	1.37	1.34	1.70	1.46	-2.27	-1.21	3
Complement C1q tumor necrosis factor-related protein 3-like isoform 1	1.11	1.07	1.52	1.24	1.91	1.86	-1.86	-1.33	3
Complement C1q-like protein 2-like	1.46	1.43	1.62	1.57	2.11	1.57	-1.95	-1.14	3
Complement component C1q receptor	1.24	1.16	1.56	1.81	1.49	1.62	1.43	1.17	3
Condensin complex subunit 1	-1.12	1.04	1.42	1.63	1.35	1.89	1.19	1.10	3
Condensin complex subunit 2	1.05	1.14	1.85	2.07	1.66	2.42	1.40	1.05	3
Condensin-2 complex subunit D3	1.58	2.14	2.24	2.05	1.85	1.98	1.61	-1.02	3
Condensin-2 complex subunit G2	1.19	1.30	1.53	1.50	1.11	1.22	-1.08	-1.13	3
COP9 signalosome complex subunit 6	-1.08	-1.15	1.09	1.12	1.09	1.20	1.14	1.02	3
CST complex subunit TEN1	1.10	1.10	1.25	1.44	1.45	1.64	1.70	1.26	3
C-X-C chemokine receptor type 7	1.12	1.10	1.01	1.14	1.32	1.35	1.42	1.11	3
Cyclin-A2	1.22	1.35	2.09	2.37	2.16	2.48	1.55	1.12	3
Cyclin-dependent kinase 1	1.20	1.36	1.99	2.40	2.19	3.12	1.75	1.13	3
Cyclin-dependent kinase 14	-1.07	-1.23	-1.04	1.05	1.11	1.26	1.15	1.04	3
Cyclin-dependent kinase 17	-1.04	1.10	1.23	1.48	1.42	1.32	1.11	1.04	3

Gene Name	Fold-Change								Cluster
	00/24	03/24	06/24	09/24	12/24	15/24	18/21	21/24	
Cyclin-dependent kinase 1-B	1.32	1.53	2.21	2.51	2.24	2.98	1.81	1.15	3
Cyclin-dependent kinase inhibitor 1	-1.30	2.18	6.00	9.79	12.84	9.80	2.81	-1.52	3
Cyclin-dependent kinases regulatory subunit 1	1.17	1.30	1.65	1.95	1.94	2.31	1.66	1.21	3
Cyclin-G2	1.06	1.19	1.33	1.59	1.55	1.78	1.32	-1.00	3
Cysteine-rich secretory protein LCCL domain-containing 1	-1.05	1.06	1.27	2.03	2.68	2.45	1.80	1.25	3
Cysteine-rich with EGF-like domain protein 2	-1.01	1.00	1.46	1.47	1.79	1.59	1.54	1.04	3
Cysteinyl-tRNA synthetase. cytoplasmic	-1.17	-1.01	1.36	1.37	1.12	1.07	1.19	1.01	3
Cytochrome b-245 heavy chain	1.17	1.98	2.06	3.01	2.71	3.34	1.76	-1.01	3
Cytochrome P450 26A1	-1.00	1.89	3.84	3.42	2.78	1.05	-1.26	-1.51	3
Cytochrome P450 2D6	1.31	1.73	1.48	1.85	1.73	1.29	1.46	1.11	3
Cytochrome P450 2K1	-1.05	1.28	-1.03	1.24	1.84	1.35	1.49	1.15	3
Cytochrome P450 4B1	1.04	-1.08	-1.21	-1.02	1.10	1.30	1.24	-1.08	3
Cytochrome P450 4X1	1.00	-1.15	-1.03	1.19	1.10	1.30	1.08	-1.11	3
Cytohesin-1	-1.02	1.15	1.30	1.73	1.54	1.55	1.39	1.16	3
Cytoplasmic dynein 1 light intermediate chain 2	-1.13	1.13	1.45	1.53	1.53	1.56	1.32	1.22	3
Cytoskeleton-associated protein 2	1.17	1.07	1.27	2.42	2.74	5.02	1.99	1.23	3
Cytosolic sulfotransferase 2	1.37	2.12	1.87	3.08	2.69	2.29	2.87	1.51	3
Cytosolic sulfotransferase 3	-1.06	1.39	1.48	1.75	1.71	1.19	-1.04	-1.21	3
D(1B) dopamine receptor	1.09	1.27	1.14	1.28	1.44	1.16	-1.09	-1.05	3
D-3-phosphoglycerate dehydrogenase	1.13	1.15	1.63	1.65	1.29	1.16	1.06	-1.14	3
DCN1-like protein 5	-1.05	1.02	1.23	1.30	1.29	1.10	1.11	-1.00	3
Dehydrodolichyl diphosphate synthase	-1.08	1.14	1.27	1.20	1.24	1.10	1.16	-1.01	3
DENN domain-containing protein 3-like	-1.11	1.03	1.20	1.32	1.52	1.42	1.12	1.08	3
Deoxynucleotidyltransferase terminal-interacting protein 1	1.06	1.03	1.13	1.26	1.24	1.23	1.14	-1.02	3
Deoxyuridine 5'-triphosphate nucleotidohydrolase. mitochondrial	1.25	1.40	2.01	1.91	1.10	1.23	1.03	-1.16	3
DEP domain-containing protein 1A	-1.22	-1.11	1.39	1.80	1.26	1.79	1.47	1.02	3
DEP domain-containing protein 1B	-1.52	-1.58	-1.25	1.61	1.34	2.68	1.45	1.33	3
Differentially expressed in FDCP 6	1.02	1.34	1.45	1.68	1.92	1.44	1.17	1.09	3
Dimethylaniline monooxygenase [N-oxide-forming] 2	2.29	1.48	3.65	3.00	4.16	2.31	3.25	1.50	3
Dimethylglycine dehydrogenase. mitochondrial	-1.04	1.48	1.39	2.06	2.21	1.76	1.73	-1.01	3
Diphosphoinositol polyphosphate phosphohydrolase 2	-1.24	-1.09	1.80	2.93	4.32	3.09	2.48	1.45	3
Disks large-associated protein 5	1.23	1.29	1.75	2.28	2.20	3.27	1.84	1.30	3
DNA excision repair protein ERCC-6-like	1.01	1.24	1.96	1.98	1.36	1.43	-1.10	-1.10	3
DNA polymerase kappa	1.00	1.09	1.13	1.38	1.37	1.30	1.22	1.01	3
DNA topoisomerase 3-beta-1	1.17	1.13	1.22	1.40	1.37	1.33	1.26	1.09	3
DNA-directed RNA polymerase II subunit RPB7	-1.00	-1.05	1.08	1.17	1.12	1.20	1.17	-1.06	3
DNA-directed RNA polymerase II subunit RPB9	-1.03	-1.09	1.12	1.12	1.17	1.30	1.08	1.01	3
DnaJ homolog subfamily A member 1	1.02	-1.11	1.15	1.14	1.15	1.24	1.42	1.26	3
DnaJ homolog subfamily B member 11	1.09	1.01	1.22	1.41	1.45	1.28	1.56	1.06	3
DnaJ homolog subfamily C member 10	1.20	1.04	1.22	1.58	1.49	1.55	1.56	1.20	3
DnaJ homolog subfamily C member 17	-1.09	-1.11	-1.03	1.17	1.10	1.31	1.31	1.06	3
DnaJ homolog subfamily C member 3	1.07	1.33	1.51	1.58	1.65	1.28	1.47	1.01	3

Gene Name	Fold-Change								Cluster
	00/24	03/24	06/24	09/24	12/24	15/24	18/21	21/24	
Dolichol phosphate-mannose biosynthesis regulatory protein	-1.14	-1.17	1.16	1.29	1.17	1.34	1.28	1.05	3
Dolichyl-diphosphooligosaccharide--protein glycosyltransferase subunit 2	1.14	1.15	1.34	1.36	1.58	1.52	1.59	1.16	3
Dolichyl-diphosphooligosaccharide--protein glycosyltransferase subunit 4	1.01	-1.04	1.11	1.25	1.19	1.33	1.33	1.13	3
Dolichyl-diphosphooligosaccharide--protein glycosyltransferase subunit DAD1	1.17	-1.04	1.12	1.31	1.17	1.38	1.49	1.02	3
Dolichyl-diphosphooligosaccharide--protein glycosyltransferase subunit STT3A	1.00	1.01	1.12	1.27	1.25	1.39	1.30	1.12	3
Dolichyl-diphosphooligosaccharide--protein glycosyltransferase subunit STT3B	1.36	1.26	1.42	1.46	1.30	1.11	1.36	1.03	3
Doublecortin domain-containing protein 2	-1.15	-1.07	1.26	1.43	1.54	1.41	1.20	1.00	3
Double-strand break repair protein MRE11A	-1.03	-1.00	1.30	1.32	1.17	1.11	1.00	-1.03	3
Dual specificity protein kinase Ttk	1.17	1.23	1.82	2.23	2.05	2.81	1.42	1.10	3
Dual specificity protein phosphatase 10	1.07	-1.12	1.11	1.18	1.11	1.55	1.22	1.09	3
Dual specificity protein phosphatase 22-B	-1.10	-1.13	-1.03	1.00	1.11	1.25	1.23	1.10	3
Dual specificity protein phosphatase 23	-1.10	-1.08	-1.07	1.16	1.09	1.25	1.21	1.13	3
Dual specificity tyrosine-phosphorylation-regulated kinase 2	1.28	1.08	1.20	1.53	1.89	1.87	1.39	1.22	3
Dual specificity tyrosine-phosphorylation-regulated kinase 4	-1.42	-1.74	-1.64	1.63	4.44	1.78	1.34	1.69	3
Dynactin subunit 5	-1.05	-1.08	-1.01	1.05	1.15	1.14	1.14	1.05	3
E3 SUMO-protein ligase CBX4	1.05	1.18	1.05	1.18	1.27	1.20	1.27	1.12	3
E3 ubiquitin-protein ligase DTX4	1.38	2.59	3.36	14.36	41.29	5.80	1.98	1.20	3
E3 ubiquitin-protein ligase HECTD1	-1.60	-1.49	-1.56	1.20	1.32	-1.14	-1.11	1.16	3
E3 ubiquitin-protein ligase RNF144B	-1.16	-1.08	-1.04	1.05	1.54	1.25	1.15	1.05	3
E3 ubiquitin-protein ligase RNF8-B	-1.05	-1.17	-1.04	-1.04	1.04	1.21	-1.01	-1.05	3
E3 ubiquitin-protein ligase UBR1	-1.04	-1.07	1.07	1.17	1.08	1.12	1.09	1.01	3
E3 ubiquitin-protein ligase ZNRF2	-1.01	1.28	1.79	2.45	2.71	2.25	1.09	-1.13	3
Egl nine homolog 1	1.02	1.16	1.46	1.42	1.48	1.35	1.37	1.04	3
EH domain-binding protein 1-like protein 1	-1.33	-1.44	1.05	1.38	1.27	1.57	1.07	1.12	3
EH domain-containing protein 4	1.02	1.06	1.49	1.78	1.56	1.59	1.52	1.16	3
Elongation of very long chain fatty acids protein 1a	-1.11	1.04	1.51	1.72	1.65	1.73	1.47	1.05	3
Endophilin-B1	-1.09	-1.05	-1.01	1.16	-1.00	1.14	1.20	-1.03	3
Endoplasmic reticulum-Golgi intermediate compartment protein 1	-1.01	1.05	1.59	1.83	1.79	1.73	1.91	1.06	3
Endoplasmin (GRP-94)	1.06	-1.08	1.07	1.44	1.46	1.53	1.60	1.17	3
Enhancer of yellow 2 transcription factor homolog B	-1.04	-1.11	-1.08	-1.02	1.04	1.15	1.08	-1.01	3
ER lumen protein retaining receptor 3	1.08	1.02	1.28	1.59	1.44	1.52	1.45	1.12	3
Eukaryotic peptide chain release factor GTP-binding subunit ERF3A	-1.16	-1.07	1.23	1.20	1.07	-1.14	-1.07	-1.22	3
Eukaryotic translation initiation factor 4 gamma 3	-1.01	-1.08	1.21	1.31	1.21	1.16	1.05	-1.05	3
Exportin-1	1.08	1.10	1.30	1.67	1.87	1.67	1.36	1.23	3
Fas-binding factor 1 homolog	-1.04	-1.10	1.00	1.38	1.42	1.59	1.37	1.08	3
FCH domain only protein 2	1.00	1.09	1.08	1.30	1.32	1.33	1.14	1.22	3
FERM. RhoGEF and pleckstrin domain-containing protein 1	1.09	1.22	1.39	1.44	1.49	1.43	1.34	1.13	3
Ferric-chelate reductase 1	-1.58	1.59	1.02	2.41	2.63	3.97	2.65	1.62	3

Gene Name	Fold-Change								Cluster
	00/24	03/24	06/24	09/24	12/24	15/24	18/21	21/24	
FGFR1 oncogene partner 2 homolog	1.09	1.03	1.15	1.18	1.15	1.40	1.33	1.14	3
Fibulin-5	1.22	1.16	1.23	1.31	1.49	1.45	1.35	1.13	3
Forkhead box protein M1	-1.02	1.19	1.79	2.30	2.01	2.86	1.65	1.09	3
Formate--tetrahydrofolate ligase	-1.13	-1.13	1.24	1.43	1.74	1.60	1.45	1.23	3
Four and a half LIM domains protein 3	-1.24	-1.05	1.23	1.40	1.67	1.65	1.36	-1.09	3
Fumarylacetoacetase	1.00	1.58	1.92	2.20	1.79	1.71	1.30	-1.25	3
G2/mitotic-specific cyclin-B1	1.18	1.07	1.43	1.73	2.27	3.50	2.21	1.35	3
G2/mitotic-specific cyclin-B2	1.23	1.43	1.59	2.52	2.48	3.88	2.55	1.60	3
G2/mitotic-specific cyclin-B3	1.10	1.04	1.35	2.02	2.31	4.04	2.17	1.45	3
Gametogenetin-binding protein 2	-1.03	1.15	1.28	1.37	1.43	1.24	1.07	-1.00	3
Gamma-interferon-inducible lysosomal thiol reductase	1.03	1.00	1.11	1.45	1.45	1.77	1.58	1.33	3
Glucagon-1	-1.07	1.33	1.59	1.95	1.56	1.68	1.92	1.04	3
Glucokinase	2.94	3.94	37.42	32.03	18.20	9.64	5.70	1.20	3
Glucose 1.6-bisphosphate synthase	1.14	1.01	1.14	1.40	1.48	1.45	1.61	1.30	3
Glucosidase 2 subunit beta	1.01	-1.06	1.23	1.33	1.51	1.40	1.34	1.13	3
Glutaminyl-peptide cyclotransferase-like protein	1.03	1.12	1.32	1.28	1.11	1.03	-1.09	-1.10	3
Glutathione peroxidase 7	1.50	1.03	2.43	3.85	3.17	3.82	3.82	1.53	3
Glutathione S-transferase A	-1.29	1.06	1.51	1.39	1.21	-1.02	-1.08	-1.10	3
Glycogen phosphorylase. liver form	-1.08	1.12	1.36	1.65	1.72	1.63	1.73	1.07	3
Glycosyltransferase 8 domain-containing protein 1	1.18	1.22	1.33	1.68	1.51	1.46	1.75	1.12	3
Glycylpeptide N-tetradecanoyltransferase 1	1.09	1.09	1.23	1.21	1.18	1.13	1.16	1.02	3
GMP reductase 2	-1.11	-1.03	1.25	1.51	1.39	1.18	1.19	1.07	3
Golgin-45	-1.09	-1.21	-1.05	1.02	1.15	1.28	1.14	1.01	3
GPI transamidase component PIG-S	1.04	1.19	1.13	1.32	1.29	1.18	1.23	1.08	3
G-protein-signaling modulator 2	-1.08	1.02	-1.02	1.53	1.50	2.51	1.60	1.41	3
Grainyhead-like protein 2 homolog	1.29	1.10	1.17	1.19	1.29	1.69	-1.33	1.22	3
Granzyme D	1.27	1.45	1.38	1.70	1.84	1.86	1.85	1.35	3
Growth factor receptor-bound protein 7	1.00	1.06	1.02	1.77	1.51	-1.03	-1.49	-1.42	3
GTP-binding protein GEM	1.18	1.26	1.43	1.56	2.55	2.05	1.07	1.29	3
GTP-binding protein yptV4	-1.12	-1.14	1.05	1.46	1.06	1.23	1.27	-1.01	3
Guanylate kinase	1.14	2.49	3.19	3.85	4.49	3.25	1.09	1.02	3
Guanylin	1.08	1.01	1.35	1.57	1.93	1.83	2.24	1.50	3
HAUS augmin-like complex subunit 8	1.05	1.06	1.21	1.29	-1.04	1.35	1.19	1.01	3
Heat shock 70 kDa protein 1	-1.16	2.19	3.55	3.58	2.99	1.61	1.92	-1.07	3
Heat shock protein beta-8	-1.15	-1.08	1.10	1.11	1.19	1.23	1.24	1.06	3
Heat shock protein HSP 90-alpha 1	1.11	1.21	1.88	3.08	2.64	2.08	1.87	1.23	3
Heme oxygenase	1.13	-1.00	1.74	1.56	1.09	1.02	1.28	-2.25	3
Heme-binding protein 1	1.08	1.09	1.07	1.61	2.17	2.12	1.79	1.31	3
Heparan sulfate 2-O-sulfotransferase 1	1.05	-1.21	-1.03	1.19	-1.01	1.19	1.35	1.03	3
Hepatocyte nuclear factor 4-alpha	-1.02	1.10	1.33	1.44	1.49	1.48	1.54	1.53	3
Hepatoma-derived growth factor	1.05	1.03	1.21	1.19	1.16	1.33	1.15	1.01	3
Hermansky-Pudlak syndrome 1 protein	-1.05	-1.10	-1.18	1.15	1.30	1.30	1.20	1.07	3
Heterogeneous nuclear ribonucleoprotein H	1.12	1.20	1.47	1.50	1.35	1.27	1.26	1.00	3

Gene Name	Fold-Change								Cluster
	00/24	03/24	06/24	09/24	12/24	15/24	18/21	21/24	
Heterogeneous nuclear ribonucleoprotein L	1.05	-1.05	1.07	1.27	1.07	1.25	1.31	1.06	3
Heterogeneous nuclear ribonucleoprotein U	1.13	1.07	1.31	1.52	1.47	1.21	1.39	1.12	3
Heterogeneous nuclear ribonucleoprotein U-like protein 1	1.22	1.25	1.37	1.55	1.64	1.34	1.16	1.17	3
Histone deacetylase 3	-1.02	-1.06	1.14	1.35	1.20	1.35	1.20	1.03	3
Histone H1	1.30	-1.04	1.57	1.62	1.19	1.89	1.10	-1.06	3
Histone H2A.Z	1.02	1.15	1.52	1.55	1.32	1.35	1.30	1.03	3
Histone H3-like centromeric protein A	-1.02	-1.05	1.24	1.53	1.70	2.61	1.98	1.28	3
Histone-lysine N-methyltransferase EHMT2	-1.02	1.04	1.17	1.31	1.35	1.22	1.04	-1.04	3
Histone-lysine N-methyltransferase SETDB1-B	1.13	1.08	1.11	1.22	1.25	1.27	1.17	1.05	3
Homeobox protein CDX-1	1.10	1.14	1.71	1.62	1.84	1.74	1.82	1.13	3
Homeobox protein six1b	-1.05	-1.31	-1.06	1.04	1.13	1.37	1.26	1.03	3
Homer protein homolog 1	-1.18	1.25	1.46	1.71	1.65	1.59	1.42	1.09	3
Homogentisate 1.2-dioxygenase	1.05	1.62	1.80	1.65	1.69	1.17	1.02	-1.48	3
Hydrolethalus syndrome protein 1 homolog	-1.14	-1.14	1.15	1.26	1.27	1.79	1.15	1.16	3
Hypothetical protein LOC100693993	1.10	3.47	7.12	9.55	5.47	1.56	-1.00	-1.06	3
Hypothetical protein LOC100701215	1.17	1.14	1.16	1.10	1.50	1.45	1.34	1.50	3
Hypothetical protein LOC100708826	-1.19	-1.06	1.04	1.01	1.06	1.14	-1.17	-1.07	3
Immediate early response gene 5-like protein	-1.10	-1.01	1.12	1.19	1.07	-1.05	-1.24	-1.16	3
Immunoglobulin superfamily member 5	-1.01	1.38	1.52	1.86	2.06	1.90	1.84	-1.03	3
Importin subunit alpha-2	1.14	-1.05	1.39	2.21	2.87	5.57	2.67	1.52	3
Inactive serine/threonine-protein kinase VRK3	1.00	1.08	1.16	1.39	1.42	1.32	1.33	1.13	3
Inhibitor of growth protein 1	1.01	1.10	1.10	1.19	1.29	1.18	1.10	1.02	3
Inhibitor of growth protein 2	-1.02	-1.01	1.14	1.23	1.17	1.27	1.06	-1.06	3
Inhibitor of nuclear factor kappa-B kinase-interacting protein	1.09	-1.04	1.70	2.02	1.81	1.92	1.68	1.05	3
Inner centromere protein	1.02	1.13	1.80	2.24	2.00	2.88	1.61	1.18	3
Inner centromere protein A	1.16	1.42	1.65	2.15	1.87	2.52	1.85	1.40	3
Inner centromere protein B	-1.07	-1.01	1.71	2.30	1.90	2.93	1.56	1.18	3
Inner ear-specific collagen	1.19	1.35	1.67	2.38	2.89	1.99	-1.41	-1.23	3
Inosine-5'-monophosphate dehydrogenase 1	-1.32	-1.18	-1.06	1.58	1.56	1.07	-1.37	-1.27	3
Inositol oxygenase	-2.03	-1.31	-1.34	1.05	1.46	1.54	1.61	1.12	3
Integrator complex subunit 11	1.03	1.19	1.36	1.23	1.35	1.17	1.17	1.07	3
Integrator complex subunit 12	-1.21	1.02	1.17	1.24	1.26	1.01	1.15	-1.08	3
Interaptin	1.26	1.30	2.00	2.73	2.50	2.14	1.97	1.051	3
Interleukin-1 receptor-like 2	1.07	1.04	1.18	1.65	1.82	1.98	1.42	1.03	3
Interleukin-17 receptor D	1.05	1.45	1.45	1.65	1.84	1.54	-1.04	-1.05	3
Intraflagellar transport protein 52 homolog	1.04	1.04	1.03	1.18	1.21	1.36	1.27	1.04	3
Intraflagellar transport protein 80 homolog	1.15	1.27	1.46	1.88	2.16	2.08	1.83	1.28	3
Inversin	-1.04	-1.10	-1.02	1.14	1.31	1.50	1.31	1.01	3
Isocitrate dehydrogenase [NADP] cytoplasmic	-1.02	1.15	1.32	1.30	1.06	-1.01	-1.03	-1.19	3
Janus kinase and microtubule-interacting protein 1	1.13	1.26	1.24	1.24	1.28	1.22	-1.06	-1.03	3
JmjC domain-containing protein 8	1.13	1.14	1.56	2.07	2.08	2.23	2.17	1.44	3
Katanin p60 ATPase-containing subunit A1	-1.06	-1.07	1.18	1.33	1.28	1.39	1.07	1.04	3
KDEL motif-containing protein 1	1.22	1.05	1.24	1.42	1.21	1.43	1.38	1.03	3

Gene Name	Fold-Change								Cluster
	00/24	03/24	06/24	09/24	12/24	15/24	18/21	21/24	
Methylosome subunit pICln	-1.00	1.14	1.29	1.30	1.24	1.21	1.06	-1.04	3
MIF4G domain-containing protein B	-1.06	1.12	1.09	1.17	1.33	1.13	-1.01	-1.13	3
Mis18-binding protein 1	-1.23	1.27	1.47	1.67	1.57	2.05	1.64	1.41	3
Mitochondrial import receptor subunit TOM34	-1.17	-1.07	1.14	1.11	1.07	1.05	1.05	-1.02	3
Mitochondrial Rho GTPase 1-A	1.02	1.23	1.33	1.30	1.36	1.06	1.05	-1.00	3
Mitotic checkpoint protein BUB3	1.19	1.34	1.73	1.87	1.60	1.55	1.26	1.01	3
Mitotic checkpoint serine/threonine-protein kinase BUB1 beta	1.11	1.27	1.44	1.73	1.39	1.86	1.40	1.13	3
Mitotic spindle assembly checkpoint protein MAD2A	1.09	1.17	1.97	2.36	2.11	2.77	1.51	1.04	3
Mitotic-spindle organizing protein 1	1.07	-1.03	1.14	1.23	1.13	1.39	1.30	1.08	3
MLN64 N-terminal domain homolog	1.02	1.06	1.09	1.25	1.47	1.60	1.43	1.11	3
Mortality factor 4-like protein 1	1.10	1.14	1.43	1.51	1.42	1.37	1.29	1.03	3
Motile sperm domain-containing protein 2	-1.09	1.15	1.49	1.74	1.75	1.71	1.25	-1.01	3
Mucin-3A	-1.13	-1.03	1.22	1.44	1.31	1.16	1.11	-1.06	3
Multiple coagulation factor deficiency protein 2 homolog	-1.03	1.05	-1.01	1.08	1.23	1.37	1.34	1.09	3
Myelin expression factor 2	1.03	-1.02	1.10	1.22	1.29	1.32	1.23	1.01	3
Myeloid leukemia factor 1	-1.21	1.07	1.75	1.97	2.11	2.24	1.88	-1.03	3
Myocyte-specific enhancer factor 2C	-1.05	1.03	1.10	1.25	1.54	1.36	1.31	1.51	3
Myomegalin	1.12	1.11	1.16	1.41	1.55	1.54	1.19	1.03	3
Myosin heavy chain. non-muscle	-1.24	-1.05	1.26	1.92	1.77	3.04	1.70	1.45	3
Myosin heavy chain. striated muscle	-1.04	1.06	1.39	1.94	1.78	2.57	1.49	1.18	3
Myosin light chain kinase family member 4	-1.31	1.04	1.27	1.19	1.11	-1.14	-1.05	-1.09	3
Myosin-Va	1.01	1.33	1.57	1.85	1.90	1.44	1.18	1.03	3
Myosin-Vc	1.02	1.79	2.07	3.17	4.37	3.64	2.29	1.13	3
Myosin-VI	1.18	1.37	1.36	1.76	2.12	2.08	1.52	1.23	3
Myotubularin	1.31	1.37	1.31	1.43	1.40	1.44	1.52	1.32	3
N-acetyltransferase 14	1.10	1.13	1.04	1.03	1.26	1.22	1.17	1.17	3
NADH-ubiquinone oxidoreductase chain 4L	1.23	1.27	1.63	1.55	1.51	1.36	1.22	1.22	3
N-alpha-acetyltransferase 38. NatC auxiliary subunit	1.02	-1.16	-1.01	1.13	1.04	1.23	1.13	1.01	3
NEDD8	-1.04	-1.11	1.08	1.17	1.09	1.31	1.27	1.07	3
Neuroepithelial cell-transforming gene 1 protein	1.11	1.13	1.21	1.32	1.16	1.77	1.51	1.06	3
Neurofilament heavy polypeptide	1.02	1.16	1.55	1.97	1.70	2.27	1.42	1.15	3
Neuron navigator 2	1.11	1.16	1.28	1.55	1.98	1.56	1.41	1.34	3
Ninein-like protein	1.04	1.08	1.14	1.29	1.43	1.59	1.19	1.08	3
Ninjurin-1	-1.42	1.02	1.13	1.03	1.18	1.16	1.06	1.20	3
NIPA-like protein 3	-1.04	-1.12	-1.11	1.03	1.11	1.13	1.02	1.06	3
Nitric oxide synthase-interacting protein	1.17	1.08	1.21	1.24	1.31	1.38	1.21	1.13	3
N-lysine methyltransferase SETD8	-1.29	-1.24	-1.20	1.14	1.27	2.24	1.71	1.37	3
Nostrin	1.03	-1.03	1.02	1.24	1.23	1.30	1.51	1.00	3
NSFL1 cofactor p47	1.16	1.24	1.36	1.44	1.43	1.51	1.56	1.17	3
NTF2-related export protein 2	-1.10	-1.14	1.03	1.11	1.09	1.12	1.03	-1.04	3
Nuclear cap-binding protein subunit 1	1.18	1.16	1.48	1.54	1.48	1.21	1.07	1.02	3
Nuclear factor erythroid 2-related factor 3	-1.15	1.02	1.20	1.12	1.12	-1.05	-1.25	-1.16	3

Gene Name	Fold-Change								Cluster
	00/24	03/24	06/24	09/24	12/24	15/24	18/21	21/24	
Nuclear factor interleukin-3-regulated protein	-1.76	2.14	1.71	3.26	1.57	2.07	-1.18	-1.24	3
Nuclear migration protein nudC	-1.09	-1.05	1.18	1.13	1.01	-1.12	-1.03	-1.17	3
Nuclear mitotic apparatus protein 1	1.01	-1.10	1.49	2.37	2.56	4.65	2.11	1.42	3
Nuclear pore complex protein Nup133	1.24	1.39	1.74	1.86	1.68	1.30	1.27	1.03	3
Nuclear pore complex protein Nup85	1.20	1.26	1.53	1.56	1.29	1.09	1.10	-1.08	3
Nuclear pore complex protein Nup93	-1.09	1.09	1.56	1.62	1.32	1.21	1.14	-1.03	3
Nuclear protein 1	-1.08	-1.69	1.09	1.75	1.94	4.24	3.10	1.59	3
Nuclear receptor coactivator 7	1.00	2.60	3.63	4.06	3.22	1.02	1.14	-1.45	3
Nuclear receptor subfamily 0 group B member 2	1.25	1.55	4.72	4.35	3.20	1.81	-1.06	-2.23	3
Nuclear receptor subfamily 1 group D member 1	1.39	-1.22	-1.04	4.37	15.19	22.75	36.43	21.2	3
Nuclear receptor subfamily 2 group F member 5	1.17	1.30	1.30	1.43	1.50	1.12	1.14	1.01	3
Nucleolar transcription factor 1-like	1.24	1.23	1.49	1.45	1.43	1.22	1.13	1.02	3
Nucleoporin NUP188 homolog	-1.35	-1.02	1.31	1.21	1.20	1.06	-1.09	1.02	3
Nucleoporin NUP53	1.07	1.20	1.48	1.62	1.48	1.46	1.32	1.09	3
Nucleoside diphosphate kinase 6	-1.01	1.00	1.11	1.36	1.20	1.26	1.17	1.07	3
Nucleoside diphosphate kinase. mitochondrial	-1.47	-1.01	1.00	1.56	2.66	2.61	1.93	1.33	3
NudC domain-containing protein 1	1.11	1.07	1.25	1.31	1.14	1.16	1.05	-1.02	3
NXPE family member 3	-1.01	-1.21	-1.18	1.58	1.21	1.84	2.60	1.41	3
Occludin	1.06	1.51	1.50	2.11	2.72	2.46	1.68	1.01	3
Opsin-5	1.14	1.08	1.24	1.54	2.22	4.03	2.24	2.62	3
Oral-facial-digital syndrome 1 protein homolog	-1.09	-1.13	-1.20	1.00	1.13	1.51	1.34	1.01	3
OTU domain-containing protein 6B	-1.01	1.01	1.19	1.26	1.16	1.17	1.22	1.00	3
Pancreatic progenitor cell differentiation and proliferation factor A	-1.24	-1.48	-1.09	1.35	1.60	1.74	1.56	1.54	3
Patched domain-containing protein 3	1.35	6.78	4.47	4.51	3.25	6.06	2.52	-1.09	3
Peptide-N(4)-(N-acetyl-beta-glucosaminyl) asparagine amidase	-1.10	-1.09	-1.05	1.06	1.11	1.18	1.07	-1.01	3
Peptidyl-prolyl cis-trans isomerase E	-1.09	-1.08	1.10	1.09	1.03	-1.03	-1.08	-1.13	3
Peptidyl-prolyl cis-trans isomerase FKBP10	1.26	1.05	1.50	1.77	1.63	1.70	1.66	1.17	3
Peptidyl-prolyl cis-trans isomerase FKBP14	1.11	1.05	1.30	1.56	1.49	1.50	1.34	1.04	3
Peptidyl-prolyl cis-trans isomerase FKBP8	-1.00	-1.08	-1.07	1.15	1.14	1.42	1.31	1.13	3
Peptidyl-prolyl cis-trans isomerase FKBP9	1.28	1.04	1.37	1.71	1.55	1.80	1.50	1.19	3
Peptidyl-prolyl cis-trans isomerase H	1.04	1.01	1.30	1.50	1.31	1.47	1.38	1.10	3
Peptidyl-prolyl cis-trans isomerase NIMA-interacting 4	1.01	-1.08	1.19	1.15	1.15	1.15	1.19	1.03	3
Peptidyl-prolyl cis-trans isomerase-like 1	-1.18	-1.12	1.28	1.41	1.18	1.26	1.15	-1.11	3
Peptidyl-prolyl cis-trans isomerase-like 3	-1.08	-1.13	1.26	1.38	1.35	1.34	1.25	-1.06	3
Peroxisomal membrane protein PMP34	1.01	-1.04	1.19	1.29	1.21	1.58	1.45	1.17	3
PHD finger-like domain-containing protein 5A	1.01	-1.10	1.21	1.31	1.30	1.42	1.36	1.09	3
Phosphatidylinositol 4-kinase beta	1.01	1.04	1.15	1.36	1.54	1.36	1.26	1.07	3
Phosphatidylinositol N-acetylglucosaminyltransferase subunit H	-1.30	1.13	1.09	1.52	1.58	1.40	1.63	1.49	3
Phosphatidylinositol-3.4.5-trisphosphate 3-phosphatase TPTE2	1.17	1.02	-1.02	1.34	1.10	1.31	1.66	1.12	3
Phosphatidylinositol-glycan biosynthesis class F protein	1.01	1.07	1.17	1.24	1.28	1.22	1.39	1.11	3
Phosphatidylinositol-glycan biosynthesis class X protein	-1.09	-1.14	-1.04	1.14	1.20	1.40	1.25	1.09	3

Gene Name	Fold-Change								Cluster
	00/24	03/24	06/24	09/24	12/24	15/24	18/21	21/24	
Phosphatidylserine decarboxylase proenzyme	1.13	1.18	1.45	1.31	1.22	1.06	1.07	-1.06	3
Phospholipase DDHD1	1.01	1.36	1.47	1.55	1.35	1.05	-1.07	1.02	3
Phosphoserine aminotransferase	1.06	1.06	1.44	1.50	1.29	1.42	1.27	1.06	3
Phosphoserine phosphatase	-1.01	1.19	1.53	1.40	1.15	1.05	1.14	-1.11	3
PIH1 domain-containing protein 1	-1.06	-1.06	1.09	1.10	1.16	1.11	1.01	1.01	3
Plasma glutamate carboxypeptidase	1.09	1.08	1.22	1.35	1.27	1.46	1.40	1.24	3
Pleckstrin homology domain-containing family A member 8	-1.01	-1.06	-1.05	1.08	1.25	1.27	1.10	1.04	3
Pleckstrin homology domain-containing family G member 2	-1.50	1.13	1.01	1.63	1.18	1.57	1.65	1.10	3
Poly [ADP-ribose] polymerase 12	1.01	-1.03	1.60	2.63	2.84	2.85	1.77	1.25	3
Polyadenylate-binding protein 2	1.25	1.30	1.53	1.53	1.50	1.17	1.07	1.01	3
Polypeptide N-acetylgalactosaminyltransferase 5	1.05	1.04	1.14	1.21	1.24	1.35	1.29	1.07	3
Polyubiquitin-C	1.13	-1.29	1.02	1.46	1.73	2.17	1.63	1.17	3
Porphobilinogen deaminase	-1.03	1.10	1.37	1.35	1.13	1.03	1.28	-1.02	3
Potassium channel subfamily K member 18	-1.00	1.01	1.15	1.87	1.71	1.55	1.39	1.03	3
PQ-loop repeat-containing protein 3	1.37	1.28	1.87	2.23	1.98	2.04	2.08	1.36	3
PRELI domain-containing protein 1. mitochondrial	-1.01	1.37	1.75	1.79	1.90	1.21	1.22	-1.15	3
Pre-mRNA-processing factor 19	1.02	1.16	1.46	1.54	1.40	1.06	1.13	-1.03	3
PRKR-interacting protein 1 homolog	-1.11	-1.02	1.07	1.15	1.21	1.27	1.18	1.12	3
Probable 2-ketogluconate reductase	1.15	1.08	1.27	1.33	1.07	1.52	1.53	-1.01	3
Probable ATP-dependent RNA helicase DHX36	1.05	1.05	1.26	1.40	1.26	1.25	1.21	1.05	3
Probable ATP-dependent RNA helicase YTHDC2	-1.01	1.06	1.73	1.85	1.40	1.37	1.13	1.03	3
Probable D-tyrosyl-tRNA(Tyr) deacylase 2	-1.17	-1.10	1.06	1.32	1.21	1.52	1.65	-1.06	3
Probable G-protein coupled receptor 125	1.23	1.23	1.30	1.47	1.74	1.58	1.44	1.16	3
Probable histone deacetylase 1-B	1.11	1.11	1.43	1.59	1.43	1.35	1.26	-1.01	3
Probable isoprenylcysteine alpha-carbonyl methylesterase ICMEL1	1.13	1.16	1.27	1.29	1.48	1.24	1.18	1.08	3
Probable N-acetyltransferase camello	-1.10	-1.07	-1.14	-1.01	1.07	1.19	1.11	-1.04	3
Probetacellulin	1.29	1.39	1.93	1.94	1.92	2.95	3.10	1.58	3
Programmed cell death 1 ligand 1	1.08	1.79	1.73	2.30	2.48	7.70	2.83	1.56	3
Proline-rich protein 15-like protein A	-1.19	1.94	2.55	3.73	3.71	4.05	2.65	1.66	3
Prolyl 3-hydroxylase 3-like	1.17	-1.03	1.23	1.27	1.27	1.64	1.35	1.18	3
Proteasome subunit alpha type-1	-1.07	-1.02	1.13	1.05	1.11	1.30	1.45	1.10	3
Proteasome subunit alpha type-5	1.01	-1.02	1.20	1.24	1.11	1.33	1.42	1.08	3
Proteasome subunit alpha type-6	-1.01	-1.10	1.09	1.17	1.09	1.30	1.19	1.01	3
Proteasome subunit beta type-1	-1.00	-1.06	1.05	1.19	1.05	1.25	1.19	1.05	3
Proteasome subunit beta type-2	1.03	-1.08	1.05	1.13	1.14	1.27	1.26	1.10	3
Proteasome subunit beta type-7	1.03	1.04	1.13	1.13	1.10	1.26	1.29	1.04	3
Protein angel homolog 1	1.12	-1.10	1.03	1.30	1.59	2.07	2.12	1.58	3
Protein aurora borealis	1.15	1.33	2.16	3.21	2.56	3.67	2.12	1.02	3
Protein canopy 4	-1.01	1.08	1.11	1.33	1.29	1.39	1.63	1.19	3
Protein CASC5	1.01	-1.04	1.70	2.10	1.56	2.55	1.53	-1.00	3
Protein catecholamines up	-1.02	-1.05	1.18	1.41	1.38	1.44	1.42	1.15	3
Protein cornichon homolog 4	-1.16	-1.07	1.08	1.19	1.10	1.19	1.11	1.01	3

Gene Name	Fold-Change								Cluster
	00/24	03/24	06/24	09/24	12/24	15/24	18/21	21/24	
Protein deltex-1	1.03	1.14	1.32	3.78	7.43	2.04	1.27	1.03	3
Protein disulfide-isomerase	-1.07	1.13	1.26	1.46	1.42	-1.01	1.07	-1.02	3
Protein disulfide-isomerase A4	1.28	-1.04	1.23	2.00	1.70	1.75	2.31	1.37	3
Protein disulfide-isomerase A6	-1.08	-1.16	1.30	1.62	1.63	1.54	1.69	1.08	3
Protein dpy-30 homolog	1.02	-1.17	1.10	1.08	1.16	1.30	1.26	1.04	3
Protein ECT2	1.14	1.26	1.89	2.33	2.35	3.76	1.73	1.30	3
Protein ERGIC-53	1.02	-1.04	1.21	1.23	1.16	1.40	1.46	1.13	3
Protein FAM118B	1.13	1.31	1.69	1.59	1.49	1.48	1.17	1.09	3
Protein FAM132B	1.08	1.02	1.29	2.04	2.49	2.74	1.76	1.20	3
Protein FAM13B	-1.35	1.07	1.42	1.64	1.44	-1.12	-1.28	-1.06	3
Protein FAM193B	1.13	1.22	1.27	1.34	1.24	1.18	1.12	1.06	3
Protein FAM54A	1.05	1.05	1.36	1.74	1.97	3.04	1.87	1.43	3
Protein FAM83D	1.10	1.23	1.44	1.67	1.48	2.14	1.32	1.12	3
Protein jagged-2-like isoform X1	-1.10	-1.13	-1.08	-1.05	1.08	1.15	-1.05	1.02	3
Protein KIAA0664	1.12	1.44	1.93	2.02	1.96	1.46	-1.04	-1.13	3
Protein kish-B	1.01	-1.05	1.14	1.22	1.25	1.19	1.12	-1.10	3
Protein MANBAL	-1.06	-1.08	-1.03	1.08	1.15	1.24	1.09	1.06	3
Protein Mis18-alpha	1.16	1.31	1.66	1.57	1.36	1.73	1.28	1.04	3
Protein NDRG1	1.11	1.64	2.27	3.70	4.19	2.69	1.82	1.09	3
Protein regulator of cytokinesis 1	1.07	-1.01	1.67	2.44	2.46	3.84	1.88	1.34	3
Protein S100-A13	-1.07	-1.09	-1.26	1.32	1.51	1.94	2.40	1.34	3
Protein saal1	-1.03	1.08	1.45	1.45	1.32	1.33	1.37	1.03	3
Protein SEC13 homolog	1.15	1.17	1.26	1.75	1.46	1.33	1.68	1.25	3
Protein Spindly	1.16	1.27	2.02	2.33	1.88	2.52	1.53	1.08	3
Protein transport protein Sec24C	1.17	1.45	1.29	1.72	1.66	1.47	1.19	1.41	3
Protein transport protein Sec61 subunit gamma	-1.09	-1.14	1.03	-1.00	1.14	1.13	1.19	1.01	3
Protein UXT	1.19	1.09	1.49	1.58	1.57	1.59	1.63	1.09	3
Protein Wnt-5a	1.20	-1.01	1.11	1.32	1.33	1.47	1.19	1.09	3
Protein yippee-like 2	1.04	-1.12	-1.48	1.33	1.27	1.61	1.04	1.40	3
P-selectin-like	-1.27	1.13	1.09	1.93	1.67	1.87	1.22	1.22	3
PTB domain-containing engulfment adapter protein 1	-1.01	-1.10	1.01	1.03	1.16	1.30	1.14	1.04	3
Pterin-4-alpha-carbinolamine dehydratase	-1.01	1.20	1.33	1.36	1.21	1.15	-1.08	-1.24	3
Putative oxidoreductase yteT	1.00	1.05	1.12	1.17	1.30	1.69	1.60	1.03	3
Putative TRAF4-associated factor 1	-1.26	-1.03	1.39	1.82	1.59	2.70	1.59	1.16	3
PWWP domain-containing protein MUM1L1	-1.06	-1.04	1.15	1.17	1.22	1.29	1.26	1.10	3
Rab5 GDP/GTP exchange factor	1.08	1.32	1.52	1.64	1.72	1.45	1.46	1.15	3
Rab-like protein 5	-1.11	-1.15	1.03	1.05	1.12	1.40	1.23	1.09	3
Rac GTPase-activating protein 1	1.30	1.32	1.61	2.16	1.80	2.26	1.80	1.24	3
Ran GTPase-activating protein 1	1.21	1.23	1.48	1.53	1.36	1.35	1.29	1.17	3
Ras and Rab interactor 2	1.19	1.25	1.53	1.81	1.71	1.58	1.33	1.08	3
Ras and Rab interactor 3	-1.22	-1.19	-1.29	1.14	1.52	1.73	1.43	1.19	3
Ras association domain-containing protein 5	-1.01	1.03	1.07	1.04	1.21	1.04	-1.21	-1.12	3
Ras association domain-containing protein 6	1.04	1.06	1.01	1.14	1.07	1.10	-1.27	-1.17	3

Gene Name	Fold-Change								Cluster
	00/24	03/24	06/24	09/24	12/24	15/24	18/21	21/24	
Ras-related protein Rab-11B	1.02	1.13	1.12	1.29	1.27	1.17	1.24	1.01	3
Ras-related protein Rab-1B	1.01	1.06	1.15	1.21	1.20	1.09	1.10	-1.03	3
Ras-related protein Rab-32	-1.01	-1.00	1.23	1.46	1.52	1.53	1.48	1.27	3
Ras-related protein Rab-9A	-1.24	-1.18	1.06	1.30	1.26	1.46	1.55	1.11	3
Ras-related protein Rap-2b	-1.16	-1.03	1.22	1.40	1.47	1.26	-1.21	-1.21	3
Receptor tyrosine-protein kinase erbB-3	1.13	1.06	1.19	1.37	1.36	1.47	-1.01	-1.08	3
Regulator of G-protein signaling 4	-1.48	-1.14	-1.15	1.02	1.25	-1.02	1.57	1.08	3
Relaxin-3 receptor 1	1.06	1.07	-1.03	1.22	1.51	1.76	1.42	1.18	3
Repressor of RNA polymerase III transcription MAF1 homolog	1.28	1.48	1.53	1.50	1.70	1.41	1.23	-1.04	3
Reticulon-4-interacting protein 1 homolog. mitochondrial	-1.06	-1.06	1.04	1.25	1.08	1.08	1.37	1.11	3
Rho GTPase-activating protein 11A	1.12	1.16	1.55	2.05	1.76	2.82	1.46	1.24	3
Rho-related GTP-binding protein RhoE	-1.10	-1.00	1.12	1.42	1.36	1.28	-1.04	-1.12	3
Rho-related GTP-binding protein RhoQ	-1.19	1.03	1.21	1.31	1.41	1.41	1.34	1.28	3
Ribonucleoside-diphosphate reductase large subunit	1.65	2.47	3.71	3.24	2.09	1.65	1.21	-1.05	3
RILP-like protein 2	-1.13	-1.16	1.02	1.15	1.16	1.39	1.46	1.21	3
RING finger protein 223	1.06	1.95	1.76	2.34	4.03	11.15	3.41	1.60	3
RNA polymerase II subunit A C-terminal domain phosphatase SSU72	1.02	1.02	1.19	1.31	1.32	1.25	1.18	1.01	3
RNA/RNP complex-1-interacting phosphatase	-1.18	1.14	1.38	-1.05	1.99	-1.32	-1.09	1.29	3
RNA-binding protein 4	-1.02	1.22	1.31	1.34	1.17	1.04	1.08	1.02	3
RNA-binding protein 42	-1.03	-1.07	1.04	1.14	1.12	1.17	1.07	-1.04	3
RNA-binding protein 47	-1.22	-1.11	1.11	1.71	1.89	1.90	1.67	1.24	3
RNA-binding protein 8A	1.06	-1.00	1.20	1.22	1.05	1.19	1.26	-1.01	3
RNA-binding protein with serine-rich domain 1	1.09	1.07	1.38	1.44	1.32	1.23	1.18	-1.06	3
RuvB-like 2	1.17	1.18	1.54	1.56	1.27	1.10	1.17	-1.05	3
S phase cyclin A-associated protein in the endoplasmic reticulum	1.04	1.20	1.24	1.48	1.64	1.37	1.17	-1.08	3
SAM domain-containing protein SAMSN-1	1.18	1.22	1.21	1.34	1.98	1.56	1.36	1.15	3
SAP domain-containing ribonucleoprotein	-1.10	-1.21	1.17	1.33	1.18	1.30	1.33	1.04	3
Scavenger mRNA-decapping enzyme DcpS	-1.02	-1.05	1.10	1.32	1.27	1.34	1.32	1.06	3
Secretogranin-3	1.03	1.14	1.14	1.45	1.77	1.32	1.18	1.28	3
Securin-2	1.02	-1.12	1.16	1.74	1.83	3.45	2.11	1.53	3
Selenoprotein Pa	-1.00	-1.08	1.19	1.24	1.22	1.21	1.28	1.00	3
Semaphorin-3aa	1.16	1.11	1.24	1.25	1.57	1.43	1.25	1.18	3
Septin-5	-1.01	-1.10	-1.07	1.10	1.32	1.21	1.18	1.05	3
Serine incorporator 3	-1.27	1.36	1.59	2.69	2.05	1.42	1.20	1.11	3
Serine protease HTRA1	1.26	1.28	1.80	2.06	2.45	2.15	1.26	-1.08	3
Serine protease HTRA1A	1.11	-1.15	1.19	1.37	1.34	1.74	1.22	1.17	3
Serine protease HTRA1B	1.01	-1.01	1.43	1.82	2.04	1.96	1.14	-1.07	3
Serine/arginine-rich splicing factor 2	-1.01	-1.00	1.19	1.26	1.09	1.16	1.08	-1.03	3
Serine/threonine-protein kinase greatwall	1.44	1.54	1.99	2.10	1.87	2.84	1.89	1.61	3
Serine/threonine-protein kinase haspin	1.29	1.59	2.04	2.22	1.99	2.37	1.68	1.10	3
Serine/threonine-protein kinase Nek2	-1.01	-1.10	1.06	1.37	1.62	2.72	1.77	1.26	3
Serine/threonine-protein kinase PAK 7	1.06	-1.08	1.10	1.40	1.66	1.82	1.44	1.26	3

Gene Name	Fold-Change								Cluster
	00/24	03/24	06/24	09/24	12/24	15/24	18/21	21/24	
Serine/threonine-protein kinase PLK1	-1.17	1.07	1.68	2.35	2.82	4.17	2.36	1.48	3
Serine/threonine-protein kinase PLK4	1.02	-1.09	1.18	1.25	1.00	1.46	-1.05	-1.14	3
Serine/threonine-protein kinase TAO3	1.13	1.55	1.43	1.73	2.03	1.68	1.48	1.27	3
Serine/threonine-protein phosphatase 2A regulatory subunit B'' subunit gamma	-1.10	-1.13	-1.11	1.00	1.01	1.13	-1.03	-1.06	3
Serine/threonine-protein phosphatase 4 catalytic subunit B	1.07	1.24	1.40	1.38	1.39	1.11	1.17	-1.02	3
Serine/threonine-protein phosphatase 4 regulatory subunit 3B	-1.00	1.02	1.11	1.20	1.14	1.16	1.05	-1.05	3
Serine-rich adhesin for platelets-like isoform X1	-1.17	-1.16	-1.05	1.27	1.18	1.47	1.25	1.24	3
Serpin A3-5	-1.45	-1.01	1.21	1.20	1.42	1.37	1.34	-1.11	3
Serpin H1	1.12	1.14	1.47	1.51	1.32	1.18	1.03	-1.17	3
SH2 domain-containing adapter protein B	-1.03	1.06	-1.01	1.14	1.43	1.44	1.40	1.10	3
SH3 domain-containing protein 19	-1.08	1.00	1.07	1.20	1.27	1.11	-1.13	-1.09	3
SHC SH2 domain-binding protein 1	1.05	1.28	1.69	1.67	1.24	1.16	-1.21	-1.40	3
Shugoshin-like 1	1.32	1.35	2.05	2.59	2.45	3.05	1.94	1.25	3
Sideroflexin-4	1.05	1.11	1.29	1.35	1.23	1.04	1.05	-1.23	3
Signal peptidase complex subunit 1	-1.00	-1.05	1.09	1.21	1.27	1.26	1.28	1.17	3
Signal recognition particle receptor subunit beta	1.03	-1.02	1.22	1.29	1.28	1.22	1.33	1.04	3
Similar to heterogeneous nuclear ribonucleoprotein k	-1.48	8.67	7.32	5.75	7.06	-1.58	1.25	-1.22	3
SLAIN motif-containing protein-like	1.20	1.37	1.39	1.29	1.37	1.08	1.09	1.09	3
Small nuclear ribonucleoprotein E	1.01	-1.00	1.21	1.33	1.21	1.31	1.25	1.01	3
Small nuclear ribonucleoprotein Sm D2	1.04	1.01	1.32	1.34	1.18	1.21	1.18	-1.05	3
Small ubiquitin-related modifier 2	1.01	-1.05	1.23	1.23	1.22	1.18	1.11	-1.01	3
Small ubiquitin-related modifier 3	1.03	-1.00	1.09	1.34	1.35	1.29	1.22	1.03	3
Sodium/bile acid cotransporter	1.09	1.29	1.12	1.58	1.72	1.62	1.74	1.11	3
Sodium-dependent neutral amino acid transporter B(0)AT3	-1.95	2.22	3.68	2.71	3.61	2.07	1.94	1.27	3
Solute carrier family 12 member 1	-1.03	-1.06	1.06	2.06	2.81	3.24	2.44	1.86	3
Solute carrier family 12 member 2	1.01	1.02	1.20	2.12	2.86	3.11	2.42	1.85	3
Solute carrier family 2. facilitated glucose transporter member 10	1.17	-1.11	1.10	1.44	1.40	1.73	1.73	1.16	3
Solute carrier family 22 member 3	1.02	1.94	1.99	2.33	2.63	1.28	1.30	1.23	3
Solute carrier family 25 member 36	-1.16	1.34	2.66	5.49	5.31	2.81	1.60	1.19	3
Solute carrier family 25 member 42	-1.33	-1.23	-1.44	1.06	1.39	2.03	1.64	1.21	3
Solute carrier family 35 member E3	1.03	1.06	1.34	1.56	1.28	1.23	1.23	-1.02	3
Somatostatin-2	-1.39	1.26	1.18	1.05	1.44	-1.00	-1.12	1.04	3
Sorting nexin-2	1.16	1.39	1.62	1.76	1.66	1.43	1.43	1.20	3
SOSS complex subunit C	-1.05	-1.04	1.25	1.38	1.44	1.30	1.18	1.00	3
Sperm-associated antigen 5	1.15	1.36	1.99	2.89	2.11	2.92	1.59	1.09	3
Sphingomyelin phosphodiesterase 3	-1.16	-1.07	1.03	1.07	1.25	1.10	1.10	1.10	3
Sphingosine-1-phosphate phosphatase 1	-1.05	-1.02	1.11	1.34	1.47	1.40	1.51	1.08	3
Spindle and kinetochore-associated protein 1	1.06	1.18	1.78	1.88	1.76	2.39	1.70	-1.01	3
Spindle and kinetochore-associated protein 3	1.02	1.15	1.44	1.36	-1.11	1.39	-1.13	-1.25	3
Splicing factor 3A subunit 3	1.11	1.08	1.23	1.31	1.25	1.11	1.15	-1.01	3

Gene Name	Fold-Change								Cluster
	00/24	03/24	06/24	09/24	12/24	15/24	18/21	21/24	
Splicing factor 3B subunit 4	1.20	1.29	1.43	1.64	1.61	1.56	1.45	1.11	3
Splicing factor. proline- and glutamine-rich	1.16	1.19	1.37	1.56	2.02	1.82	1.56	1.20	3
Spondin-2	-1.01	1.04	1.24	2.31	3.32	5.04	3.48	1.93	3
SPRY domain-containing SOCS box protein 3	-1.06	1.31	1.34	1.84	1.96	1.72	1.37	1.09	3
Steroidogenic acute regulatory protein. mitochondrial	-1.16	1.44	1.60	1.55	1.25	1.07	1.88	1.57	3
Stonustoxin subunit beta-like	-1.33	1.87	5.80	9.34	11.97	11.23	4.97	2.13	3
Stonustoxin subunit beta-like. partial	-1.11	-1.10	1.11	1.48	1.77	1.72	1.41	1.27	3
Sugar phosphate exchanger 2	1.14	1.07	1.02	1.14	1.38	1.33	1.48	1.23	3
SUMO-activating enzyme subunit 1	-1.00	-1.08	1.29	1.51	1.28	1.58	1.49	1.11	3
Surfeit locus protein 1	-2.17	5.77	3.69	3.96	5.73	-2.07	-1.93	-1.92	3
SWI/SNF-related matrix-associated actin-dependent regulator of chromatin subfamily B member 1	1.09	1.08	1.19	1.37	1.35	1.33	1.22	1.07	3
Synaptonemal complex protein SC65	1.41	1.27	1.59	1.85	1.88	1.80	1.83	1.25	3
Syntaxin-12	-1.01	-1.03	1.05	1.19	1.19	1.24	1.13	1.04	3
Syntaxin-7	-1.02	1.01	1.00	1.19	1.22	1.17	1.10	1.05	3
Syntaxin-binding protein 3	-1.19	-1.12	-1.07	1.06	-1.02	1.13	1.10	1.01	3
Targeting protein for Xklp2-B	-1.05	-1.02	1.20	1.70	1.63	2.37	1.46	1.15	3
TATA box-binding protein-like protein 1	1.05	1.15	1.35	1.72	1.57	1.65	1.36	1.05	3
Tetraspanin-1	1.14	1.15	1.41	1.65	1.80	2.02	1.86	1.29	3
Tetratricopeptide repeat protein 12	-1.07	-1.07	-1.06	1.05	1.12	1.36	1.10	1.02	3
Tetratricopeptide repeat protein 38	1.22	1.46	1.57	1.67	1.69	1.47	1.17	-1.06	3
Thioredoxin domain-containing protein 12	-1.03	-1.15	1.17	1.20	1.16	1.27	1.18	1.04	3
Thioredoxin reductase 2. mitochondrial	-1.06	1.26	1.30	1.17	1.07	1.10	1.28	1.14	3
Thioredoxin-like protein 4B	-1.02	-1.18	-1.03	1.00	1.20	1.32	1.14	1.06	3
THO complex subunit 3	-1.00	1.12	1.64	1.60	1.62	1.56	1.33	1.02	3
Threonyl-tRNA synthetase. cytoplasmic	-1.09	-1.09	1.26	1.50	1.28	1.32	1.31	-1.02	3
Tissue factor pathway inhibitor 2	-1.05	1.15	1.39	1.74	2.06	1.81	1.84	1.27	3
TLR4 interactor with leucine rich repeats-like	1.22	1.28	1.70	2.29	2.41	2.80	1.75	1.22	3
TRAF family member-associated NF-kappa-B activator	1.09	1.10	1.28	1.24	1.40	1.36	1.20	-1.00	3
Traf2 and NCK-interacting protein kinase	-1.45	-1.28	1.01	1.35	1.69	1.92	1.53	1.26	3
Transcription elongation factor A protein 1	1.11	1.06	1.23	1.30	1.30	1.33	1.24	1.03	3
Transcription factor 12	-1.19	-1.04	-1.01	1.14	1.17	1.30	1.51	1.22	3
Transcription factor AP-2-alpha	1.05	-1.15	-1.10	1.06	1.21	1.31	-1.59	-1.12	3
Transcription factor Dp-1	1.07	-1.01	1.15	1.31	1.13	1.28	1.14	1.05	3
Transcription initiation factor IIB	1.04	-1.03	1.03	1.12	1.15	1.16	1.20	1.04	3
Transcription initiation protein SPT3 homolog	-1.15	-1.10	-1.22	1.13	1.19	1.25	1.27	1.19	3
Transcription termination factor 2	-1.08	1.15	1.14	1.39	1.38	1.51	1.35	-1.02	3
Transducin beta-like protein 2	-1.09	1.05	1.36	1.37	1.34	1.19	1.22	-1.04	3
Transforming acidic coiled-coil-containing protein 3	1.16	1.06	1.62	2.55	2.76	4.29	2.13	1.34	3
Transforming growth factor beta-3	-1.31	-1.21	-1.06	1.07	1.08	1.30	1.09	1.06	3
Transitional endoplasmic reticulum ATPase	-1.05	1.07	1.11	1.45	1.21	1.51	1.37	1.10	3
Translocating chain-associated membrane protein 1-like 1	1.09	1.15	1.50	1.66	1.62	1.41	1.45	1.06	3

Gene Name	Fold-Change								Cluster
	00/24	03/24	06/24	09/24	12/24	15/24	18/21	21/24	
Translocating chain-associated membrane protein 2	-1.02	-1.12	1.21	1.75	1.80	2.07	1.73	1.29	3
Translocon-associated protein subunit delta	1.03	-1.01	1.11	1.16	1.12	1.20	1.28	1.09	3
Transmembrane 6 superfamily member 2	1.06	1.11	1.29	1.49	1.70	1.55	2.21	1.33	3
Transmembrane and coiled-coil domain-containing protein 6	-1.07	-1.18	-1.00	1.12	1.16	1.38	1.20	-1.11	3
Transmembrane emp24 domain-containing protein 3	-1.12	-1.08	-1.03	1.20	1.29	1.38	1.34	1.00	3
Transmembrane protein 165	-1.08	-1.02	1.17	1.30	1.19	1.25	1.13	1.00	3
Transmembrane protein 18	-1.03	-1.10	-1.04	1.00	1.07	1.24	1.09	1.01	3
Transmembrane protein 198-B	1.13	-1.05	1.08	1.18	1.23	1.44	1.28	1.19	3
Transmembrane protein 206	-1.11	-1.01	-1.03	1.16	1.63	1.50	1.46	1.14	3
Transmembrane protein 214-A	-1.04	1.22	1.59	1.93	1.94	1.77	1.53	-1.01	3
Transmembrane protein 33	1.04	1.18	1.23	1.45	1.43	1.18	1.28	1.12	3
Transmembrane protein 43	-1.03	-1.00	1.19	1.37	1.24	1.52	1.65	1.16	3
Tripartite motif-containing protein 14	1.04	1.00	-1.10	1.11	1.12	1.34	1.13	1.02	3
Tripeptidyl-peptidase 1	-1.10	-1.18	1.01	1.19	1.36	1.67	1.56	1.17	3
Tubulin alpha-1A chain	-1.02	-1.01	1.24	1.26	1.29	1.41	1.20	-1.03	3
Tubulin alpha-1B chain	1.06	1.00	1.47	1.58	1.61	1.76	1.68	1.28	3
Tubulin alpha-1D chain	-1.03	-1.02	1.21	1.31	1.28	1.38	1.28	-1.02	3
Tubulin beta chain	1.12	1.09	1.49	1.54	1.43	1.46	1.31	1.02	3
Tubulin beta-5 chain	1.12	1.08	1.78	2.51	2.32	2.47	2.88	1.53	3
Tubulin beta-6 chain	1.23	1.16	1.55	1.62	1.60	1.56	1.46	-1.01	3
Tubulin polymerization-promoting protein family member 3	1.23	2.36	4.04	4.91	6.19	3.29	1.65	-1.09	3
Tumor necrosis factor alpha-induced protein 8-like protein 3	-1.03	1.02	1.40	1.80	1.94	2.17	1.44	1.17	3
Tumor necrosis factor receptor superfamily member 11A	1.08	-1.16	1.07	1.15	1.29	1.49	1.06	-1.05	3
Tumor necrosis factor receptor type 1-associated DEATH domain protein	-1.01	1.23	1.51	1.69	1.84	1.67	1.41	1.03	3
Type II inositol-1.4.5-trisphosphate 5-phosphatase	1.15	1.27	1.37	1.56	1.58	1.29	1.18	1.04	3
Tyrosine-protein kinase Lyn	1.28	1.07	1.54	1.55	1.96	1.74	1.66	1.10	3
Tyrosine-protein phosphatase non-receptor type 6	1.23	1.14	1.24	1.27	1.41	1.57	1.71	1.19	3
Tyrosyl-tRNA synthetase. cytoplasmic	1.02	1.20	1.64	1.63	1.34	1.10	1.29	1.17	3
U4/U6 small nuclear ribonucleoprotein Prp31	1.05	1.09	1.27	1.34	1.33	1.25	1.21	1.02	3
U4/U6.U5 tri-snRNP-associated protein 2	-1.08	-1.09	1.28	1.27	1.10	1.02	1.01	-1.13	3
Ubiquitin carboxyl-terminal hydrolase 14	-1.03	1.18	1.36	1.43	1.33	1.42	1.51	1.03	3
Ubiquitin carboxyl-terminal hydrolase 28	-1.17	-1.19	-1.06	1.70	2.24	2.14	1.54	1.18	3
Ubiquitin carboxyl-terminal hydrolase 3	1.20	1.25	1.41	1.48	1.63	1.52	1.57	1.27	3
Ubiquitin-conjugating enzyme E2 C	1.08	1.08	1.62	2.10	2.14	3.07	1.68	1.17	3
Ubiquitin-conjugating enzyme E2 G1	-1.07	-1.08	-1.03	1.23	1.14	1.28	1.16	1.04	3
Ubiquitin-conjugating enzyme E2 S	-1.06	1.16	1.20	1.35	1.43	1.81	1.63	1.17	3
Ubiquitin-related modifier 1 homolog	-1.04	-1.12	1.06	1.30	1.17	1.17	1.16	1.05	3
UBX domain-containing protein 1	-1.20	-1.15	-1.02	1.14	-1.00	1.11	1.08	-1.02	3
UDP-glucose 6-dehydrogenase	1.08	1.17	1.67	1.81	1.92	1.30	1.45	1.11	3
UDP-glucuronosyltransferase 2B13	1.03	1.67	1.95	1.62	1.40	1.31	1.73	1.18	3

Gene Name	Fold-Change								Cluster
	00/24	03/24	06/24	09/24	12/24	15/24	18/21	21/24	
UDP-glucuronosyltransferase 2B2	-1.13	-1.28	-1.38	1.16	1.30	1.35	1.73	1.08	3
UDP-N-acetylhexosamine pyrophosphorylase	1.08	1.24	1.41	1.51	1.12	1.07	-1.16	-1.30	3
UHRF1-binding protein 1-like	1.03	1.07	1.30	1.37	1.54	1.37	1.07	1.05	3
Uncharacterized oxidoreductase ZK1290.5	-1.00	1.07	1.30	1.62	1.75	1.91	1.43	1.16	3
Uncharacterized protein C10orf71	-1.21	-1.16	1.17	1.84	1.50	2.62	1.92	1.58	3
Uncharacterized protein C12orf32 homolog	1.06	1.15	1.48	1.74	1.56	1.88	1.15	-1.18	3
Uncharacterized protein C17orf62 homolog	-1.55	-1.42	1.10	1.23	1.17	1.34	1.35	1.40	3
Uncharacterized protein C1orf172 homolog	1.05	1.27	1.12	1.12	1.34	1.21	-1.11	-1.14	3
Uncharacterized protein C4orf29 homolog	1.12	1.13	1.22	1.37	1.34	1.32	1.34	1.04	3
Uncharacterized protein C6orf132	-1.02	1.25	1.17	1.64	1.56	1.41	1.42	1.25	3
Uncharacterized protein C7orf36	-1.13	-1.03	1.09	1.17	1.11	1.07	-1.05	-1.08	3
Uncharacterized protein KIAA1107	1.06	1.19	1.12	1.32	1.45	1.28	1.20	1.08	3
Uncharacterized protein LOC101162159	-1.10	-1.01	1.06	1.47	1.85	1.75	-1.18	1.11	3
Uncharacterized protein LOC101469315 isoform X1	-1.27	1.60	5.38	7.14	4.75	4.24	1.93	-1.04	3
Uncharacterized protein LOC101470459	1.07	-1.01	1.31	1.58	2.71	2.45	1.56	1.59	3
Uncharacterized protein LOC101482294	1.03	1.31	1.54	1.64	2.56	1.92	1.26	1.15	3
Uncharacterized protein ORF91	-1.20	1.94	-1.16	1.73	5.61	-1.17	1.18	-1.27	3
Uncharacterized protein R102.4	1.01	-1.00	1.27	1.38	1.45	1.43	1.43	1.04	3
UPF0406 protein C16orf57 homolog	1.01	-1.09	-1.12	1.01	1.10	1.46	1.15	-1.04	3
UPF0454 protein C12orf49 homolog	1.02	1.31	1.24	1.35	1.35	1.48	1.50	1.40	3
UPF0554 protein C2orf43 homolog	-1.15	1.26	1.20	1.78	1.74	1.38	1.33	1.00	3
UPF0556 protein C19orf10 homolog	1.04	-1.03	1.09	1.33	1.34	1.36	1.55	1.16	3
UPF0568 protein C14orf166 homolog	-1.05	-1.01	1.31	1.34	1.25	1.22	1.28	1.01	3
UPF0670 protein C8orf55 homolog	-1.07	1.38	1.33	1.39	1.54	1.31	1.86	1.43	3
UPF0676 protein C1494.01	-1.03	-1.00	1.05	1.12	1.33	1.38	1.11	1.05	3
UPF0683 protein C7orf47 homolog	1.05	1.05	1.13	1.24	1.29	1.18	1.03	-1.01	3
UPF0760 protein C2orf29	-1.02	1.05	1.27	1.33	1.31	1.15	1.04	-1.01	3
Uridine-cytidine kinase 2-B	-1.16	-1.09	1.27	1.51	1.44	1.18	1.11	-1.01	3
Urotensin-2B-like	-1.40	1.23	1.10	1.32	1.36	1.18	1.47	1.17	3
UV excision repair protein RAD23 homolog A	-1.05	-1.06	1.05	1.08	-1.00	1.12	1.20	1.01	3
UV excision repair protein RAD23 homolog B	-1.02	-1.03	1.11	1.18	1.10	1.23	1.22	1.07	3
Uveal autoantigen with coiled-coil domains and ankyrin repeats protein	1.10	1.02	1.16	1.27	1.34	1.95	1.75	1.64	3
Vacuolar protein sorting-associated protein 53 homolog	-1.08	-1.02	-1.01	1.24	1.23	1.22	1.14	-1.01	3
Vacuolar protein-sorting-associated protein 25	1.03	-1.02	1.12	1.26	1.18	1.26	1.19	-1.01	3
Vascular endothelial growth factor A-A	-1.10	-1.07	-1.01	1.06	1.26	1.24	1.12	1.02	3
Vascular endothelial growth factor receptor 3-like	1.01	1.15	1.02	1.15	1.27	1.23	1.38	1.20	3
Vasohibin-2	-1.13	1.05	1.12	1.59	1.94	2.26	1.95	1.76	3
VIP36-like protein	1.09	1.01	1.08	1.32	1.28	1.21	1.34	1.14	3
Vitamin D 25-hydroxylase	-1.49	-1.08	1.06	1.34	1.42	2.56	2.23	1.53	3
WD repeat domain-containing protein 83	-1.09	1.56	2.11	3.76	2.06	2.09	2.43	1.58	3
WD repeat-containing protein 18	-1.04	1.06	1.07	1.22	1.50	1.29	1.18	1.08	3
WD repeat-containing protein 26	-1.63	-1.29	-1.22	-1.04	1.07	1.18	-1.04	-1.05	3

Gene Name	Fold-Change								Cluster
	00/24	03/24	06/24	09/24	12/24	15/24	18/21	21/24	
WD repeat-containing protein WRAP73	1.12	1.17	1.39	1.49	1.80	2.07	1.44	1.13	3
Wee1-like protein kinase	1.13	1.44	1.71	1.83	1.40	1.25	-1.00	-1.05	3
X-ray repair cross-complementing protein 5	1.01	1.03	1.26	1.44	1.53	1.64	1.52	1.10	3
Zinc finger CCCH-type with G patch domain-containing protein	1.05	1.15	1.54	1.54	1.42	1.21	1.28	1.11	3
Zinc finger CCHC domain-containing protein 24	-1.01	-1.05	1.17	1.22	1.17	1.25	1.33	1.01	3
Zinc finger matrin-type protein 5	-1.05	-1.12	-1.06	1.04	1.18	1.24	1.29	1.14	3
Zinc finger protein 10	1.09	1.30	1.40	1.25	1.40	1.23	1.07	-1.07	3
Zinc finger protein 345	1.08	1.35	1.51	1.53	1.52	1.60	1.31	-1.02	3
Zinc finger protein 479	1.09	-1.16	1.00	-1.02	1.14	1.36	1.19	1.01	3
Zinc finger protein 483	1.22	1.11	1.31	1.28	1.37	1.15	-1.03	-1.27	3
Zinc finger protein 595	-1.00	-1.18	-1.02	1.23	1.25	1.73	1.60	1.22	3
Zinc finger protein 70	-1.05	-1.10	1.08	1.11	1.29	1.48	1.28	1.11	3
Zinc finger protein 846	-1.05	-1.31	-1.13	1.14	1.59	1.60	1.08	1.31	3
Zinc finger protein 879	-1.15	-1.20	-1.15	1.04	1.06	1.18	1.09	-1.06	3
Zinc transporter ZIP13	1.05	-1.15	1.01	1.26	1.18	1.33	1.19	1.09	3
Zinc transporter ZIP4	1.17	1.23	1.42	2.12	2.06	2.26	1.64	-1.10	3
Zona pellucida-like domain-containing protein 1-like	1.21	1.66	2.87	4.24	5.48	3.59	1.29	1.20	3
28S ribosomal protein S21. mitochondrial	-1.02	-1.07	-1.27	-1.27	-1.32	-1.41	-1.03	-1.07	4
28S ribosomal protein S7. mitochondrial	-1.10	-1.03	-1.07	-1.14	-1.17	-1.25	1.03	-1.00	4
55 kDa erythrocyte membrane protein	1.01	-1.13	-1.53	-1.56	-1.82	-1.58	-1.13	-1.03	4
5'-AMP-activated protein kinase subunit beta-1	-1.01	-1.14	-1.32	-1.18	-1.05	1.15	1.13	1.12	4
5'-nucleotidase domain-containing protein 2	1.22	-1.19	-1.42	-1.35	-1.59	1.04	1.09	1.06	4
6-phosphofructo-2-kinase/fructose-2.6-biphosphatase 1	1.21	-1.28	-1.35	-1.45	-1.78	-1.20	1.12	1.11	4
A disintegrin and metalloproteinase with thrombospondin motifs 8	-1.17	-1.28	-2.39	-2.30	-2.33	-2.09	-1.20	-1.10	4
A disintegrin and metalloproteinase with thrombospondin motifs 9	1.17	-1.08	-1.18	-1.05	1.11	1.29	1.26	1.22	4
Abhydrolase domain-containing protein 14A	-1.00	-1.07	-1.24	-1.16	1.03	1.07	1.11	1.06	4
Abhydrolase domain-containing protein 2-B	-1.40	-1.81	-2.22	-2.39	-2.06	-1.15	-1.20	-1.06	4
Acetylcholine receptor subunit alpha	1.07	-1.58	-1.73	-1.72	-1.90	-1.02	1.05	1.05	4
Acetylcholine receptor subunit delta	-1.14	-1.38	-1.51	-1.71	-1.74	-1.29	-1.23	-1.09	4
Acetylcholine receptor subunit gamma	1.16	-1.23	-1.33	-1.36	-1.43	-1.04	1.14	1.07	4
Acetyl-CoA carboxylase 2	1.04	-1.23	-1.64	-1.67	-1.40	-1.47	-1.20	-1.07	4
Acyl-CoA dehydrogenase family member 11	1.00	-1.03	-1.32	-1.15	-1.12	1.24	1.42	1.15	4
Adenylate cyclase type 7	-1.56	-1.45	-2.89	-1.26	-1.27	-1.25	-1.02	-1.12	4
ADM2	-1.18	1.18	-1.09	-1.22	-1.17	-1.21	1.16	1.21	4
ADP-ribosylation factor-binding protein GGA1	1.26	1.02	-1.17	-1.33	-1.26	-1.06	1.01	1.24	4
ADP-ribosylation factor-related protein 1	-1.10	-1.11	-1.43	-1.73	-1.58	-1.44	-1.14	-1.01	4
Adrenodoxin-like protein. mitochondrial	1.03	-1.04	-1.30	-1.46	-1.50	-1.26	-1.13	-1.13	4
AF4/FMR2 family member 1	-1.32	-1.15	-2.06	-1.22	-1.06	1.11	1.46	1.06	4
A-kinase anchor protein 6	-1.58	-1.15	-1.48	1.01	1.01	1.60	1.48	1.29	4
Alanine--glyoxylate aminotransferase 2-like 2	-1.15	-1.12	-1.37	-1.29	-1.13	1.10	1.11	1.09	4
Alpha N-terminal protein methyltransferase 1B	-1.08	-1.52	-1.15	-1.27	-1.30	1.54	1.66	1.62	4
Alpha-1B adrenergic receptor-like	1.07	1.08	-1.26	-1.56	-1.41	-1.51	1.00	1.08	4

Gene Name	Fold-Change								Cluster
	**00/24**	**03/24**	**06/24**	**09/24**	**12/24**	**15/24**	**18/21**	**21/24**	
Alpha-galactosidase A	-1.10	1.11	-1.16	-1.04	1.15	1.20	1.28	1.23	4
Alpha-glucosidase 2	1.04	-1.06	-2.01	-1.77	-1.19	1.02	1.45	1.08	4
AMP deaminase 2-like. partial	-1.04	-1.07	-1.39	-1.50	-1.48	-1.69	-1.09	1.07	4
AMY-1-associating protein expressed in testis 1	-1.23	1.05	-1.57	-1.55	-1.21	-1.28	1.03	-1.02	4
AN1-type zinc finger protein 5	-1.05	-1.98	-2.72	-1.98	-1.30	1.39	1.68	2.30	4
Angiopoietin-2	-1.52	-1.13	-1.54	-1.58	-1.49	-1.23	1.33	1.24	4
Angiotensin-converting enzyme	1.32	-1.21	-1.92	-1.88	-1.65	-1.08	1.09	1.45	4
Ankyrin repeat and SOCS box protein 10	-1.05	-3.03	-3.17	-1.84	-1.16	3.18	2.79	2.70	4
Ankyrin repeat and SOCS box protein 3	1.02	-1.01	-1.26	-1.17	-1.18	-1.06	1.16	1.12	4
Ankyrin repeat and SOCS box protein 8	1.07	1.03	-1.13	1.10	1.26	1.47	1.40	1.55	4
Ankyrin repeat domain-containing protein 1	1.77	-1.10	-1.25	-1.63	-1.66	-1.16	1.32	1.49	4
Ankyrin repeat domain-containing protein 33	-1.20	-1.61	-3.37	-2.21	1.20	-1.43	-1.28	1.48	4
Anoctamin-8	1.08	-1.08	-1.27	-1.17	-1.05	-1.13	1.07	1.20	4
Anthrax toxin receptor 2	-1.14	-1.21	-1.39	-1.20	-1.12	1.36	1.22	1.07	4
Apolipoprotein L domain-containing protein 1	-1.06	-1.06	-1.26	-1.11	1.25	1.75	1.19	1.36	4
Aquaporin-4	-1.13	-1.39	-1.73	-1.86	-1.99	-1.66	-1.51	-1.03	4
Arrestin domain-containing protein 2	-1.03	-2.33	-3.17	-2.65	-2.15	1.21	1.11	2.05	4
Aryl hydrocarbon receptor 2	1.03	1.02	-1.27	-1.29	1.05	1.18	1.50	1.16	4
Arylacetamide deacetylase	-1.05	-1.32	-1.64	-1.66	-1.20	1.44	2.05	1.85	4
ATP-binding cassette sub-family C member 9	-1.90	-1.23	-2.01	-1.70	-2.11	-1.36	1.06	1.26	4
ATP-binding cassette sub-family D member 4	1.04	-1.04	-1.43	-1.10	-1.13	1.13	1.15	-1.04	4
Autophagy-related protein 2 homolog B	-1.13	-1.30	-1.27	-1.14	-1.10	1.36	1.19	1.09	4
Autophagy-related protein 9A-like	-1.21	-1.19	-1.48	-1.43	-1.35	1.00	1.04	1.19	4
Baculoviral IAP repeat-containing protein 4	-1.09	-1.41	-1.82	-1.98	-1.90	-1.70	-1.22	1.20	4
Band 4.1-like protein 5	-2.15	-1.69	-1.98	-1.59	-1.23	-1.26	-1.13	-1.17	4
Basic leucine zipper transcriptional factor ATF-like	1.12	-1.34	-1.04	-1.71	-1.08	1.31	1.20	2.03	4
Calmodulin-regulated spectrin-associated protein 1-B	-1.36	-1.23	-2.25	-2.02	-1.82	-1.79	-1.17	1.23	4
Carbohydrate sulfotransferase 7	-1.01	1.06	-1.13	-1.21	-1.17	-1.20	-1.03	1.12	4
Carbonyl reductase family member 4	1.03	-1.11	-1.21	-1.44	-1.24	-1.09	-1.01	1.02	4
Carboxy-terminal domain RNA polymerase II polypeptide A small phosphatase 2	-1.09	-1.40	-2.03	-2.60	-2.69	-1.80	-1.68	-1.22	4
Carnitine O-palmitoyltransferase 1. liver isoform	-1.05	-1.07	-1.91	-1.82	-1.32	-1.04	1.30	1.46	4
Casein kinase I isoform delta	-1.25	-1.33	-2.32	-1.84	-3.00	-1.53	-1.38	-1.05	4
Caseinolytic peptidase B protein homolog	1.06	1.10	-1.22	-1.20	-1.15	-1.25	-1.01	-1.00	4
Caspase-3	-1.09	-1.18	-1.20	-1.04	1.02	1.21	1.11	1.028	4
Caspase-9	-1.29	-1.21	-1.68	-1.26	-1.18	1.34	1.21	1.00	4
Cat eye syndrome critical region protein 2	-2.63	1.25	-1.79	-1.95	-1.72	-1.65	1.56	2.00	4
Cation transport regulator-like protein 1	-1.00	-2.95	-4.22	-3.96	-2.04	1.75	2.37	2.94	4
Cbp/p300-interacting transactivator 3	1.02	-1.34	-1.54	-1.19	-1.08	1.33	1.26	1.47	4
C-C chemokine receptor type 11	1.06	-1.26	-1.54	-1.92	-2.20	-1.17	-1.09	1.17	4
C-C chemokine receptor type 9	-1.20	-1.74	-1.51	-1.51	-1.31	1.27	1.31	-1.02	4
CCAAT/enhancer-binding protein delta	-1.33	-1.74	-1.80	-3.21	-2.02	-1.42	-1.61	1.50	4
CCR4-NOT transcription complex subunit 2	1.14	-1.03	-1.29	-1.42	-1.33	-1.26	1.02	-1.07	4

Gene Name	Fold-Change								Cluster
	00/24	03/24	06/24	09/24	12/24	15/24	18/21	21/24	
CD83 antigen	-1.18	-1.94	-1.53	-2.09	-1.70	-1.14	-1.04	-1.02	4
Cdc42-interacting protein 4 homolog	-1.19	-1.33	-1.55	-2.26	-2.19	-1.35	-1.41	-1.05	4
CDGSH iron-sulfur domain-containing protein 3. mitochondrial	-1.18	-1.15	-1.43	-1.30	-1.11	-1.20	1.07	1.05	4
Cell division cycle and apoptosis regulator protein 1	1.02	-1.09	-1.26	-1.24	-1.10	1.09	1.15	1.23	4
Centrin-1	-1.08	-1.13	-1.35	-1.20	1.03	1.26	1.06	1.09	4
Centrosomal protein kizuna	-1.18	-1.16	-1.46	-1.44	-1.36	-1.15	-1.10	-1.00	4
Ceramide synthase 1	-1.02	-1.19	-1.55	-1.60	-1.65	-1.45	-1.26	1.09	4
Ceroid-lipofuscinosis neuronal protein 5	-1.08	-1.22	-1.35	-1.28	-1.26	1.03	1.06	1.01	4
CGMP-dependent protein kinase 2	1.18	-1.02	-1.12	-1.04	1.22	1.52	1.91	1.57	4
Charged multivesicular body protein 1a	-1.01	-1.06	-1.20	-1.21	-1.21	-1.01	-1.05	1.09	4
Claudin-10	1.08	1.43	-1.01	-1.55	1.01	1.17	1.77	1.80	4
Claudin-19	-1.03	1.00	-1.03	-1.01	1.03	1.20	1.40	1.15	4
CMRF35-like molecule 3	-1.40	1.17	-1.45	-1.62	-1.23	-1.06	1.25	1.18	4
CMRF35-like molecule 7	1.94	2.29	-1.01	1.28	1.10	1.48	2.69	1.16	4
C-Myc-binding protein	-1.03	-1.13	-1.11	-1.18	1.06	1.15	1.03	-1.00	4
Coagulation factor V	-1.51	-1.32	-1.49	-2.03	-1.81	-1.53	-1.63	-1.22	4
Coenzyme Q-binding protein COQ10 homolog. mitochondrial	-1.47	-1.16	-1.85	-1.34	-1.01	1.12	1.62	1.01	4
Coiled-coil domain-containing protein 115	-1.23	-1.31	-1.22	-1.14	-1.07	1.13	1.03	-1.05	4
Coiled-coil domain-containing protein 126	-1.12	1.17	1.02	1.03	1.14	1.09	1.35	1.15	4
Coiled-coil domain-containing protein 90B. mitochondrial	-1.11	1.12	-1.20	-1.15	-1.42	-1.14	1.29	1.17	4
Coiled-coil domain-containing protein 97	1.32	1.28	1.35	1.47	2.11	2.70	3.88	2.38	4
Coiled-coil-helix-coiled-coil-helix domain-containing protein 6	-1.13	-1.16	-1.18	-1.08	1.05	1.17	1.14	1.00	4
Collagen alpha-2(IV) chain	1.23	1.01	-1.17	-1.16	-1.08	1.17	1.34	1.31	4
Complement C1q tumor necrosis factor-related protein 2	-1.01	-1.62	-2.49	-3.12	-2.64	-1.36	-1.11	1.25	4
Complement C1q tumor necrosis factor-related protein 7	1.24	-1.04	-1.14	-1.32	-1.26	1.16	1.01	1.25	4
Complement C1q-like protein 4	1.85	1.36	-1.82	-1.06	2.50	3.69	3.53	3.15	4
Connective tissue growth factor	-1.19	-1.47	-1.78	-2.12	-2.34	-1.59	-1.77	-1.20	4
Consortin	-1.06	-1.23	-1.17	-1.03	1.11	1.39	1.27	1.19	4
Constitutive coactivator of peroxisome proliferator-activated receptor gamma	-1.27	-1.44	-1.77	-1.84	-1.37	-1.16	1.35	1.27	4
Contactin-5	-1.37	-1.51	-1.81	-1.50	-1.60	-1.01	1.26	1.68	4
Contactin-associated protein-like 5	-1.09	1.37	-1.17	1.04	-1.16	-1.11	1.67	2.13	4
Cornifelin homolog	-1.00	-1.25	-1.94	-1.79	-1.71	-1.40	1.55	1.40	4
Crumbs homolog 1	1.35	-1.28	-1.57	-1.81	-1.52	-1.49	1.36	2.33	4
Cullin-4A	-1.05	-1.14	-1.12	-1.08	-1.05	1.10	1.05	1.03	4
C-X-C chemokine receptor type 5	-1.14	-1.25	-1.97	-1.93	-1.23	1.67	1.56	1.17	4
C-X-C motif chemokine 9	1.05	-1.54	-1.29	-1.42	-1.13	1.24	1.00	1.04	4
Cyclic AMP-dependent transcription factor ATF-1	1.00	-1.14	-1.33	-1.37	-1.44	-1.17	-1.15	-1.03	4
Cyclin-D1-binding protein 1 homolog	-1.08	-1.74	-1.35	-1.11	-1.50	-1.29	-1.07	-1.10	4
Cyclin-dependent kinase inhibitor 3	1.04	-1.16	-1.26	-1.10	-1.01	1.84	1.67	1.32	4
Cyclin-dependent kinase-like 1	-1.01	-1.12	-2.03	-1.97	-1.95	-1.44	-1.44	-1.59	4

Gene Name	Fold-Change								Cluster
	00/24	03/24	06/24	09/24	12/24	15/24	18/21	21/24	
Cysteine protease ATG4C	-1.10	-1.25	-1.38	-1.27	-1.12	1.29	1.26	1.18	4
Cysteine/serine-rich nuclear protein 2	-1.22	-1.55	-2.13	-1.85	-1.62	1.08	1.28	1.85	4
Cysteine/serine-rich nuclear protein 3	-1.07	1.09	-1.19	-1.09	-1.20	-1.20	1.36	1.34	4
Cysteine-rich motor neuron 1 protein-like	-1.01	-1.19	-1.49	-1.58	-1.77	-1.26	1.01	1.17	4
Cytidine and dCMP deaminase domain-containing protein 1	1.09	-1.14	-1.20	-1.11	-1.24	1.12	1.05	1.28	4
Cytochrome b reductase 1	1.03	1.05	-1.35	-1.67	-1.49	-1.45	1.03	1.03	4
Cytochrome P450 2D9	-1.10	1.91	1.14	1.19	1.19	-1.06	1.60	3.14	4
Cytochrome P450 2U1	-1.21	-1.22	-1.67	-1.57	-1.57	-1.30	1.07	-1.05	4
Cytoglobin-1	1.07	1.19	-1.40	-1.13	-1.28	-1.07	1.27	1.21	4
DCN1-like protein 2	-1.29	-1.42	-1.51	-1.44	-1.47	-1.17	-1.17	-1.03	4
DDB1- and CUL4-associated factor 15	1.00	-1.21	-1.16	-1.05	-1.05	1.04	1.05	1.06	4
Delta-like protein 4	-1.11	-1.15	-1.54	-1.08	-1.12	1.22	1.23	1.18	4
Deoxyhypusine synthase	-1.18	-1.16	-1.35	-1.38	-1.38	-1.30	-1.34	-1.14	4
Dexamethasone-induced protein homolog	-1.18	-1.50	-1.69	-1.92	-1.84	-1.11	-1.16	-1.07	4
Dexamethasone-induced Ras-related protein 1	-1.19	-1.25	-1.49	-1.48	-1.09	-1.53	1.03	1.26	4
Dihydropyrimidine dehydrogenase [NADP+]	1.06	1.14	-1.11	-1.53	-1.60	-1.25	1.16	1.15	4
DIS3-like exonuclease 1	1.06	1.62	-1.12	-1.66	-1.01	-1.17	1.11	1.23	4
Disks large-associated protein 4	1.01	1.19	1.00	1.04	1.20	-1.01	1.26	1.26	4
DNA damage-inducible transcript 4 protein	-1.25	-1.76	-2.36	-2.45	-2.00	1.21	1.29	3.15	4
DNA damage-inducible transcript 4-like protein	-1.00	1.30	1.01	1.22	1.79	2.09	2.83	2.38	4
DNA polymerase delta subunit 4	-1.15	-1.16	-1.19	-1.17	-1.12	1.04	1.01	1.01	4
DNA-binding death effector domain-containing protein 2	-1.03	-1.11	-1.75	-1.80	-1.56	-1.30	-1.19	1.01	4
DNA-binding protein inhibitor ID-1	-1.09	-1.35	-1.44	-1.39	-1.25	-1.20	-1.16	-1.17	4
DNA-directed RNA polymerase I subunit RPA2	-1.46	-1.29	-1.77	-2.36	-2.16	-2.08	1.01	1.09	4
DNA-directed RNA polymerase II subunit RPB1	-1.36	1.12	-1.06	-1.04	-1.09	1.09	1.51	1.36	4
DnaJ homolog subfamily B member 12	1.01	-1.06	-1.05	-1.01	1.01	1.16	1.17	1.04	4
DNL-type zinc finger protein	-1.18	-1.38	-1.48	-1.52	-1.61	-1.43	-1.13	-1.10	4
DTDP-D-glucose 4.6-dehydratase	-1.23	-1.03	-1.38	1.03	-1.09	-1.10	1.20	-1.23	4
Dual specificity protein phosphatase 2	-1.29	1.27	-1.07	-1.07	1.27	1.02	1.23	2.14	4
Dynein heavy chain 12. axonemal	-1.17	-1.55	-1.46	1.09	1.12	1.66	1.61	1.27	4
Dynein light chain Tctex-type 1	-1.22	-1.22	-1.30	1.02	-1.06	1.34	1.25	1.08	4
Dystrophin	1.08	-1.26	-3.39	-1.58	-2.29	-1.34	-1.08	1.49	4
E3 ubiquitin-protein ligase CBL-B	-1.14	-1.38	-2.29	-1.80	-2.02	-1.50	-1.39	-1.32	4
E3 ubiquitin-protein ligase MARCH7	-1.33	-1.82	-1.76	-1.42	-1.34	1.29	1.20	1.33	4
E3 ubiquitin-protein ligase MARCH8	-1.04	-1.09	-1.14	1.13	1.20	1.17	1.23	1.66	4
E3 ubiquitin-protein ligase parkin	1.15	-1.02	-1.32	-1.20	-1.06	-1.03	1.01	1.04	4
E3 ubiquitin-protein ligase RNF167	-1.10	-1.44	-1.41	-1.39	-1.42	1.06	1.06	1.38	4
E3 ubiquitin-protein ligase TRIM11	1.06	-1.05	-1.46	-1.88	-2.02	-1.44	1.06	1.23	4
E3 ubiquitin-protein ligase TRIM63	-1.39	-2.69	-1.96	1.11	-1.16	3.71	3.32	2.87	4
Ectonucleotide pyrophosphatase/phosphodiesterase family member 5	1.20	-1.32	-1.56	-1.20	-1.03	1.01	1.35	1.33	4
EF-hand calcium-binding domain-containing protein 7	1.35	1.02	-1.34	-1.21	1.02	1.21	1.06	1.15	4

Gene Name	Fold-Change								Cluster
	00/24	03/24	06/24	09/24	12/24	15/24	18/21	21/24	
ELM2 and SANT domain-containing protein 1	-1.39	-1.05	-1.50	-1.62	-1.33	1.19	1.68	1.40	4
ELMO domain-containing protein 2	1.02	1.09	-1.09	1.05	1.09	1.15	1.28	1.41	4
Elongation factor 1-alpha 1	-1.44	1.61	-1.22	-1.63	1.22	-1.47	2.07	1.78	4
Elongation of very long chain fatty acids protein AAEL008004	1.03	-1.10	-1.07	1.01	1.06	1.28	1.28	1.28	4
Elongator complex protein 3	-1.52	-1.24	-2.26	-1.81	-2.08	-1.15	-1.15	-1.04	4
EMILIN-1	1.12	1.00	-1.33	-1.19	-1.01	1.28	1.21	1.27	4
Endothelial zinc finger protein induced by tumor necrosis factor alpha	-1.07	-1.38	-1.13	-1.03	1.07	1.42	1.40	1.11	4
Endothelin-converting enzyme 1	1.07	1.01	-1.44	-1.69	-1.36	-1.24	1.23	1.25	4
Epidermal growth factor receptor kinase substrate 8-like protein 1	1.12	-1.47	-1.92	-2.02	-1.22	1.02	-1.32	1.16	4
Erythroid differentiation-related factor 1	-1.09	-1.26	-1.22	-1.08	-1.12	1.02	-1.03	-1.14	4
E-selectin	-1.24	-1.31	-1.29	-1.93	-1.52	-1.58	-1.24	-1.35	4
Ethanolamine kinase	1.18	1.07	1.06	1.04	1.12	1.72	1.90	1.68	4
Ethanolamine kinase 2	-1.12	-1.05	-1.25	-1.60	-1.52	-1.15	-1.03	1.05	4
ETS translocation variant 3	-1.05	-1.10	-1.54	-1.54	-1.50	-1.23	-1.21	1.13	4
ETS translocation variant 4	-1.24	-1.40	-1.47	-1.91	-1.70	-1.11	-1.59	-1.11	4
Eukaryotic translation initiation factor 2-alpha kinase 3	-1.42	-1.45	1.00	1.12	-1.58	-1.17	1.16	1.20	4
Exocyst complex component 3-like protein 4	-1.00	-1.30	-1.40	-1.26	-1.14	-1.07	1.24	1.11	4
Exosome component 10	-1.13	-1.13	-1.23	-1.26	-1.32	-1.29	-1.12	1.00	4
Extended synaptotagmin-2-A	-1.63	-1.51	-3.12	-2.07	-2.07	-1.41	1.42	1.43	4
Eyes absent homolog 4	1.20	1.13	1.07	-1.21	-1.03	1.87	2.29	2.37	4
FAST kinase domain-containing protein 5	-1.30	-1.06	-1.28	-1.57	-1.30	-1.44	1.05	1.06	4
F-box only protein 25	1.04	-1.87	-3.37	-2.31	-1.61	1.33	1.58	1.81	4
F-box only protein 32	-2.47	-2.20	-5.83	-4.56	-3.11	-1.12	1.89	2.56	4
FERM and PDZ domain-containing protein 4-like isoform X2	-1.02	-1.13	-1.46	-1.51	-1.40	-1.43	-1.21	1.16	4
Fibroblast growth factor-binding protein 2	-1.09	-1.35	-1.68	-2.90	-2.14	-1.91	-1.60	-1.27	4
Filamin A-interacting protein 1-like	-1.00	-1.21	-1.56	-1.42	-1.22	-1.09	-1.03	1.05	4
Filamin-A-interacting protein 1	-1.04	1.02	-1.32	-1.20	-1.19	-1.08	1.30	1.05	4
Folate receptor beta	-1.18	-1.36	-2.20	-2.31	-2.57	-2.09	-1.05	1.05	4
Forkhead box protein D5-A	-1.14	1.81	1.00	1.11	1.06	-1.15	1.55	1.81	4
Forkhead box protein K1	-1.13	-1.40	-1.66	-1.70	-1.69	-1.51	-1.32	1.15	4
FXYD domain-containing ion transport regulator 6	-1.07	-1.81	-1.20	-1.01	1.15	1.68	1.50	1.35	4
FYVE and coiled coil domain containing protein 1	-1.56	-1.28	-1.48	-1.22	-1.14	1.23	1.05	1.02	4
G patch domain-containing protein 2	-2.29	-1.64	-1.41	-1.77	-1.79	1.09	-1.42	1.45	4
G1/S-specific cyclin-D2	1.01	-1.12	-1.20	-1.20	-1.16	-1.10	-1.01	-1.00	4
G1/S-specific cyclin-E1	-1.02	-1.19	-1.18	-1.57	-2.64	-1.05	1.08	1.25	4
Galectin-related protein	-1.00	-1.10	-1.41	-1.29	-1.11	1.02	1.27	1.18	4
Gamma-aminobutyric acid receptor-associated protein-like 1	-1.30	-1.31	-1.48	-1.25	1.03	1.19	1.02	1.00	4
Gap junction alpha-3 protein	1.19	-1.20	-1.15	-1.01	-1.92	-1.20	1.03	1.10	4
GATS-like protein 3	-1.03	1.03	-1.27	-1.44	-1.26	-1.20	1.04	-1.04	4
GDP-D-glucose phosphorylase C15orf58 homolog	-1.35	-1.25	-1.25	-1.08	-1.15	1.01	1.13	-1.16	4

Gene Name	Fold-Change								Cluster
	00/24	03/24	06/24	09/24	12/24	15/24	18/21	21/24	
General receptor for phosphoinositides 1-associated scaffold protein	1.06	-1.05	-1.14	-1.32	-1.14	-1.03	1.17	1.13	4
General transcription factor IIH subunit 5	-1.10	-1.18	-1.03	-1.02	-1.03	1.11	1.08	1.04	4
Glia-activating factor	-1.07	-1.02	-1.54	-1.22	-1.22	-1.19	1.20	1.15	4
Glomulin-like	1.07	-1.15	-1.66	-1.65	-1.12	1.14	1.21	1.27	4
Glycerate kinase	-1.11	-1.05	-1.18	-1.08	-1.03	1.05	1.13	1.14	4
Glycerophosphocholine phosphodiesterase GPCPD1	-1.25	-1.37	-1.94	-2.52	-2.25	-1.37	1.12	1.54	4
Glypican-5	1.17	-1.20	-1.07	-1.46	-1.16	-1.08	1.00	-1.02	4
GPN-loop GTPase 3	-1.15	-1.28	-1.35	-1.36	-1.44	-1.28	-1.21	-1.09	4
G-protein coupled receptor 26	1.14	-1.04	-1.04	-1.13	1.10	1.04	1.95	1.95	4
G-protein coupled receptor 4	-1.00	-1.11	-1.14	-1.39	-1.21	1.07	1.26	1.27	4
Granulocyte colony-stimulating factor receptor	-1.17	1.10	-1.13	-1.28	-1.02	1.01	1.28	1.317	4
Growth arrest and DNA damage-inducible protein GADD45 alpha	-1.32	-1.43	-1.44	-1.29	-1.09	1.01	1.16	-1.05	4
Growth arrest and DNA damage-inducible protein GADD45 gamma	-1.32	-1.55	-1.57	-1.91	-1.53	-1.28	1.09	-1.26	4
Growth factor receptor-bound protein 10	-1.19	-1.21	-1.68	-1.56	-1.69	-1.57	1.08	1.47	4
Growth hormone receptor type I	1.07	-1.09	-1.22	-1.25	-1.06	-1.18	-1.03	1.34	4
GTP:AMP phosphotransferase. mitochondrial	1.01	-1.13	-1.45	-1.71	-1.80	-1.67	1.04	1.18	4
GTPase-activating Rap/Ran-GAP domain-like protein 3	-1.50	1.02	-1.48	-1.45	-1.56	-1.24	1.37	1.27	4
Guanine nucleotide-binding protein G(I)/G(S)/G(O) subunit gamma-5	1.04	-1.13	-1.06	-1.03	1.08	1.22	1.06	1.09	4
Headcase protein homolog	-1.20	-1.41	-1.55	-1.47	-1.22	1.52	1.24	1.39	4
Heat shock 70 kDa protein 4L	1.41	1.28	-1.25	-1.69	-1.06	-1.38	-1.14	1.48	4
Heme transporter hrg1-A	-1.06	-1.28	-1.87	-2.02	-1.93	-1.73	-1.29	-1.05	4
Heme transporter hrg1-B	1.04	1.08	-1.16	-1.30	-1.25	-1.29	-1.06	1.16	4
Heparan sulfate glucosamine 3-O-sulfotransferase 2	1.17	1.02	-1.30	-1.51	-1.28	-1.17	-1.01	1.08	4
Heparan-alpha-glucosaminide N-acetyltransferase	1.36	1.00	-1.20	1.16	-1.10	1.36	1.29	1.25	4
High affinity cGMP-specific 3'.5'-cyclic phosphodiesterase 9A	1.06	1.27	-1.12	1.03	1.29	1.46	1.43	1.26	4
High affinity choline transporter 1	-1.03	-1.13	-1.18	-1.17	-1.10	1.12	1.39	1.22	4
Histidine triad nucleotide-binding protein 3	-1.09	-1.08	-1.08	1.02	1.06	1.11	1.19	1.05	4
Histone acetyltransferase KAT2B	1.00	1.06	-1.11	1.01	1.18	1.56	1.76	1.42	4
Histone acetyltransferase KAT5	-1.19	-1.16	-1.36	-1.27	-1.02	1.05	1.16	1.19	4
Histone H1.0	-1.14	-1.42	-1.78	-1.97	-1.46	1.13	1.21	-1.00	4
Histone H3	1.47	-1.11	-1.09	-1.73	1.05	2.64	2.80	1.73	4
HMG box transcription factor BBX	-1.13	-1.50	-1.42	-1.09	-1.03	1.38	1.06	1.22	4
HMG box-containing protein 1	1.02	-1.13	-1.58	-1.68	1.06	2.07	1.71	1.47	4
Homeobox protein MOX-1	1.12	-1.14	-1.43	-1.71	-1.54	-1.45	-1.22	-1.01	4
HSPB1-associated protein 1 homolog	-1.08	-1.20	-1.56	-1.59	-1.54	-1.10	1.18	1.25	4
Ig epsilon chain C region	1.04	-1.18	-1.24	-1.40	-1.28	-1.11	1.25	1.34	4
Inactive serine protease PAMR1	-1.05	-1.36	-1.49	-2.01	-1.87	-1.00	-1.07	1.14	4
Induced myeloid leukemia cell differentiation protein Mcl-1	-1.46	-1.41	-1.51	-1.85	-1.83	-1.27	-1.33	-1.07	4
Induced myeloid leukemia cell differentiation protein Mcl-1 homolog	1.03	-1.23	-1.41	-1.62	-1.51	-1.06	1.12	1.05	4

Gene Name	Fold-Change								Cluster
	00/24	03/24	06/24	09/24	12/24	15/24	18/21	21/24	
Inhibitor of growth protein 4	-1.05	-1.23	-1.28	-1.19	-1.11	-1.02	-1.03	-1.00	4
INO80 complex subunit E	-1.14	-1.20	-1.23	-1.08	-1.05	1.10	1.22	1.07	4
Inositol hexakisphosphate kinase 2	1.20	-1.14	-1.43	-1.51	-1.28	1.12	1.32	1.22	4
Inositol polyphosphate 5-phosphatase K	-1.15	-1.56	-1.66	-1.43	-1.64	1.18	-1.09	-1.00	4
Insulin receptor substrate 1	-1.18	-1.19	-1.52	-1.77	-1.88	-1.58	-1.40	-1.08	4
Insulin-like growth factor II	-1.27	-1.31	-1.49	-1.24	-1.22	1.00	1.14	1.03	4
Integral membrane protein GPR155	1.03	1.12	-1.93	-1.42	-2.13	-1.46	1.05	1.32	4
Integrin alpha-5	-1.51	1.07	-2.02	-1.05	-1.58	1.55	1.60	1.65	4
Integrin alpha-9	-1.20	1.02	-1.30	1.02	-1.03	1.48	1.59	1.42	4
Integrin beta-6	1.21	-1.18	-1.03	-1.08	-1.06	1.24	1.13	1.22	4
Interferon regulatory factor 5	-1.02	-1.19	-1.29	-1.26	-1.14	-1.01	-1.14	-1.05	4
Interferon-related developmental regulator 1	-1.06	-1.06	-1.13	-1.38	-1.50	-1.14	1.17	1.48	4
Intraflagellar transport protein 43 homolog B	-1.10	-1.11	-1.25	-1.23	-1.08	1.02	-1.04	-1.02	4
Intraflagellar transport protein 88 homolog	-1.14	-1.19	-1.26	-1.19	-1.20	1.10	1.22	1.10	4
Iron/zinc purple acid phosphatase-like protein	-1.04	-1.09	-1.32	-1.26	1.02	1.10	1.09	1.08	4
JmjC domain-containing protein 4	-1.04	-1.25	-1.26	-1.11	-1.03	1.34	1.16	1.12	4
Junctional adhesion molecule C	-1.29	-1.45	-1.50	-1.25	-1.37	-1.10	1.07	1.08	4
Kelch domain-containing protein 1	-1.25	-1.17	-1.48	-1.36	-1.26	-1.11	1.08	1.07	4
Kelch domain-containing protein 10	-1.02	-1.08	-1.27	-1.11	-1.12	1.39	1.28	1.36	4
Kelch-like protein 23	1.17	1.07	-1.03	1.13	1.34	1.38	1.73	1.46	4
Kelch-like protein 24	-1.56	-1.33	-2.22	-1.33	1.48	4.05	2.87	1.99	4
Kinetoplastid membrane protein 11	-2.57	-1.13	-1.86	-2.38	-2.29	-1.52	1.45	1.70	4
Laminin subunit beta-1-like	-1.15	1.03	-1.21	-1.10	1.14	1.10	1.20	1.15	4
Large neutral amino acids transporter small subunit 3	-1.22	-1.39	-2.05	-1.78	-1.86	-1.81	-1.22	1.07	4
Large neutral amino acids transporter small subunit 3-like	-1.22	-1.39	-1.88	-2.23	-2.39	-2.00	-1.41	-1.11	4
Leiomodin-1	-1.30	1.19	-1.96	-2.81	-1.92	-2.01	1.00	-1.08	4
Leucine-rich repeat-containing protein 14B	1.13	-1.04	-1.01	1.05	1.50	2.01	1.79	1.75	4
Leucine-rich repeat-containing protein 27	-1.18	-1.04	-1.13	-1.40	-1.29	-1.05	1.08	1.00	4
Leucine-rich repeat-containing protein 28	-1.05	-1.05	-1.05	1.08	-1.05	1.36	1.31	1.07	4
Leucine-rich repeat-containing protein 57	1.11	1.10	-1.20	-1.10	1.05	1.06	1.21	1.10	4
Ligand of Numb protein X 2	1.04	1.03	1.06	-1.08	1.21	1.36	1.22	1.27	4
LINE-1 reverse transcriptase homolog	-2.02	1.36	-2.18	1.20	-2.77	-1.05	1.85	1.24	4
Lipid A export ATP-binding/permease protein MsbA	1.01	1.57	1.28	1.19	1.86	2.11	3.78	1.97	4
Lipid phosphate phosphohydrolase 3	1.03	-1.30	-1.50	-1.24	-1.00	1.06	1.02	-1.01	4
Liprin-beta-2	1.02	-1.27	-1.66	-1.56	-1.46	1.04	1.20	1.22	4
L-lactate dehydrogenase B-B chain	1.34	-1.38	-1.67	-1.95	-1.61	-1.02	-1.16	1.52	4
L-lactate dehydrogenase C chain	1.21	-1.18	-1.25	-1.06	1.28	1.45	1.16	1.47	4
Low density lipoprotein receptor adapter protein 1	-1.13	-1.03	-1.22	1.00	1.53	2.00	1.63	1.51	4
Low density lipoprotein receptor adapter protein 1-A	-1.20	1.08	-1.04	-1.05	1.21	1.30	1.29	1.18	4
LOW QUALITY PROTEIN: von Willebrand factor A domain-containing protein 5B1-like	-1.10	1.10	-1.02	-1.04	1.39	-1.18	2.03	1.61	4
Low-density lipoprotein receptor 2	-1.16	-1.05	-1.71	-1.17	1.85	4.31	3.02	2.09	4

Gene Name			Fold-Change						Cluster
	00/24	03/24		00/24	03/24		00/24	03/24	
Lymphoid-restricted membrane protein	-1.07	-1.06	-1.31	-1.69	-1.32	1.18	1.49	1.85	4
LYR motif-containing protein 5A	-1.12	-1.22	-1.27	-1.15	-1.27	1.09	1.06	1.07	4
Lysine-specific demethylase 6B	1.03	-1.09	-1.08	1.04	1.01	1.17	1.15	1.13	4
Lysophosphatidylcholine acyltransferase 2	1.10	-1.20	-1.21	1.05	1.25	1.07	1.58	1.58	4
Lysozyme g	-1.22	-1.18	-1.55	-1.64	-1.57	-1.39	-1.25	-1.15	4
Major facilitator superfamily domain-containing protein 8	-1.03	-1.11	-1.30	-1.18	-1.20	1.02	1.10	1.07	4
Malonyl-CoA-acyl carrier protein transacylase. mitochondrial	-1.09	-1.25	-1.45	-1.53	-1.50	-1.46	-1.12	-1.07	4
Maltase A3	1.13	-1.28	-1.77	-1.47	-1.23	1.02	1.21	1.36	4
MAP kinase-interacting serine/threonine-protein kinase 2	-1.30	-1.69	-2.38	-2.52	-2.41	-1.38	-1.30	1.08	4
Metal transporter CNNM4	-1.69	1.17	-1.44	-1.69	-1.08	1.19	2.02	1.61	4
Metalloproteinase inhibitor 2	-1.24	-1.56	-1.42	-1.39	-1.26	1.22	1.12	1.13	4
Meteorin-like protein	-1.11	-1.34	-1.33	-1.35	1.00	1.04	1.15	1.03	4
Methenyltetrahydrofolate synthase domain-containing protein	-1.19	-1.55	-1.36	-1.16	1.01	1.38	1.34	1.15	4
Methionine aminopeptidase 1D. mitochondrial	-1.17	-1.14	-1.45	-1.49	-1.57	-1.42	-1.05	-1.18	4
Methyltransferase-like protein 12. mitochondrial	1.01	-1.21	-1.26	-1.24	-1.14	1.02	1.20	1.23	4
Methyltransferase-like protein 17. mitochondrial	-1.05	-1.01	-1.31	-1.57	-1.34	-1.32	-1.05	-1.02	4
Microtubule-associated proteins 1A/1B light chain 3 beta 2	-1.03	-1.32	-1.55	-1.61	-1.13	-1.13	1.54	1.93	4
Microtubule-associated proteins 1A/1B light chain 3B	-1.59	-1.58	-1.90	-1.99	-1.29	1.04	-1.09	1.16	4
Microtubule-associated tumor suppressor 1 homolog A	-1.00	-1.07	-1.35	-1.43	-1.24	-1.21	1.08	1.11	4
MIT domain-containing protein 1	-1.02	-1.12	-1.30	-1.08	1.04	1.15	1.28	1.09	4
Mitochondrial carnitine/acylcarnitine carrier protein	-1.44	-1.30	-1.68	-1.64	-1.49	-1.46	-1.09	-1.06	4
Mitochondrial thiamine pyrophosphate carrier	-1.01	1.12	-1.21	-1.21	-1.18	-1.08	1.14	1.12	4
Mitofusin-1	-1.07	1.12	-1.39	-1.15	-1.19	-1.25	1.08	1.06	4
Mitogen-activated protein kinase kinase kinase MLT	-1.66	-1.01	-2.60	-1.90	-1.62	-2.58	-1.06	1.05	4
Molybdenum cofactor biosynthesis protein 1	-1.10	-1.18	-1.59	-1.79	-1.54	-1.34	-1.13	1.03	4
Monocarboxylate transporter 1	-1.03	-1.08	-1.28	1.02	1.03	-1.02	1.33	1.42	4
Monocarboxylate transporter 9	-1.12	-1.33	-2.11	-2.61	-2.35	-2.02	-1.21	-1.10	4
Monocyte chemotactic protein 1B	-1.23	-1.31	-1.34	-2.19	-1.80	-1.52	-1.13	-1.05	4
MORN repeat-containing protein 5	-1.27	-1.29	-1.26	-1.11	-1.14	1.16	1.29	-1.04	4
Mothers against decapentaplegic homolog 9	-1.23	-1.38	-1.75	-1.91	-1.86	-1.57	-1.44	-1.28	4
Motile sperm domain-containing protein 1	-1.07	-1.19	-1.61	-1.55	-1.31	-1.01	1.06	1.08	4
M-phase inducer phosphatase 1	1.23	-1.10	-1.13	1.30	1.20	1.98	1.64	1.53	4
M-phase phosphoprotein 9-like	-1.69	1.19	-1.29	-1.20	1.05	1.26	1.48	1.66	4
Mpv17-like protein	1.11	-1.32	-1.15	-1.36	-1.56	-1.10	1.01	1.03	4
Multidrug resistance-associated protein 9	1.45	1.13	-1.25	-1.72	-1.73	-1.09	1.29	1.74	4
Multiple epidermal growth factor-like domains protein 8	-1.87	1.03	-1.98	-1.07	-1.07	1.16	1.44	1.11	4
Muscle. skeletal receptor tyrosine protein kinase	-1.48	-1.28	-1.71	-1.78	-2.12	-1.18	1.04	1.31	4
Muscle. skeletal receptor tyrosine-protein kinase	-1.32	-1.35	-1.54	-1.32	-1.22	-1.12	1.13	1.45	4

Gene Name	Fold-Change								Cluster
	00/24	03/24	06/24	09/24	12/24	15/24	18/21	21/24	
Muscular LMNA-interacting protein	-1.07	-1.45	-2.15	-2.88	-2.12	-2.10	-1.39	1.13	4
Myelin protein zero-like protein 3	-1.06	-1.30	-1.58	-1.61	-1.44	-1.48	-1.23	1.03	4
Myosin light chain kinase. smooth muscle	1.12	-1.04	-1.44	-1.62	-1.48	-1.43	1.00	-1.00	4
Myosin-7B	1.25	-1.15	-1.34	-1.37	-1.49	-1.11	1.32	1.09	4
Myosin-XV	1.41	1.23	-1.25	-1.41	-1.62	-1.23	-1.00	1.19	4
Natural resistance-associated macrophage protein 1	-1.61	-1.18	-1.42	-1.36	1.18	1.07	1.72	1.74	4
NCK-interacting protein with SH3 domain	-1.17	-1.08	-1.24	-1.82	-1.26	-1.27	1.09	-1.11	4
Neprilysin	-1.22	-1.29	-1.41	-1.04	1.04	1.34	1.30	1.53	4
Neuromedin-K receptor	-1.06	1.03	-1.34	-1.52	-1.36	-1.19	1.00	1.08	4
Neuronal pentraxin-1-like	-1.32	-1.60	-3.00	-2.40	-1.55	-1.34	-1.23	1.07	4
Neuropathy target esterase	-1.64	-1.49	-2.07	-2.07	-1.97	-1.29	1.06	1.12	4
Neutral and basic amino acid transport protein rBAT	1.15	-1.01	-1.15	-1.16	1.10	-1.18	2.10	1.78	4
NF-kappa-B inhibitor epsilon	-1.04	-1.27	-1.17	-1.44	-1.25	1.03	1.02	1.09	4
NF-kappa-B inhibitor zeta	1.05	-1.18	-1.28	-1.27	1.09	1.49	2.12	1.00	4
NF-kappa-B inhibitor-interacting Ras-like protein 2	-1.12	1.01	-1.35	-1.26	-1.16	-1.14	1.17	1.11	4
Nicastrin	1.02	-1.09	-1.00	-1.01	1.05	1.22	1.08	1.09	4
Nicotinate-nucleotide pyrophosphorylase [carboxylating]	-1.02	-1.01	-1.70	-1.56	-1.91	-1.84	-1.04	-1.14	4
Nicotinate-nucleotide pyrophosphorylase [carboxylating]-like isoform X1	1.02	1.15	-1.60	-1.57	-1.59	-1.50	1.12	1.07	4
Niemann-Pick C1-like protein 1	1.33	-1.02	-1.39	-1.24	-1.68	-1.13	-1.04	1.26	4
Nitrogen permease regulator 2-like protein	-1.00	-1.03	-1.16	-1.01	-1.06	1.53	1.57	1.34	4
N-sulphoglucosamine sulphohydrolase	1.01	1.02	-1.13	-1.17	1.04	1.26	1.23	1.13	4
Nuclear receptor coactivator 4	1.20	-1.00	-1.32	-1.48	-1.47	-1.22	-1.03	1.16	4
Nucleolar protein 10	-1.41	-1.61	-1.75	-2.16	-2.09	-1.80	-1.50	-1.26	4
O-acetyl-ADP-ribose deacetylase C6orf130	-1.11	-1.29	-1.46	-1.45	-1.24	1.12	-1.01	1.07	4
O-acetyl-ADP-ribose deacetylase C6orf130 homolog	-1.09	-1.24	-1.41	-1.39	-1.22	1.14	1.03	1.08	4
O-phosphoseryl-tRNA(Sec) selenium transferase	-1.15	-1.16	-1.39	-1.53	-1.54	-1.52	-1.31	-1.06	4
ORM1-like protein 1	1.02	-1.16	-1.39	-1.25	-1.13	-1.01	1.06	-1.03	4
Patatin-like phospholipase domain-containing protein 2	1.18	-1.18	-1.56	-1.62	-1.15	1.12	1.54	1.14	4
PDZ and LIM domain protein 2	1.14	1.22	-1.12	-1.44	-1.34	-1.12	1.08	1.1	4
Peptide-N(4)-(N-acetyl-beta-D-glucosaminyl)asparagine amidase F	1.01	-1.09	-1.19	1.04	1.07	1.21	1.21	1.07	4
Peptidyl-prolyl cis-trans isomerase-like 6	-1.08	1.03	-1.50	-1.28	-1.35	-1.18	1.22	1.00	4
Peripheral myelin protein 22	-1.14	-1.48	-1.37	-1.48	-1.69	-1.12	-1.18	-1.11	4
Peroxisomal membrane protein 11C	1.17	1.18	1.15	1.25	1.09	1.05	1.32	1.26	4
Peroxisomal membrane protein 4	-1.08	-1.11	-1.12	-1.05	-1.09	1.09	1.14	1.08	4
Phosphatidate phosphatase LPIN1	-1.16	-1.26	-1.38	1.23	1.72	2.70	2.80	1.87	4
Phosphatidate phosphatase PPAPDC1B	-1.11	-1.09	-1.71	-1.56	-1.47	-1.21	-1.06	-1.03	4
Phosphatidylcholine:ceramide cholinephosphotransferase 1	1.11	1.38	-1.02	1.73	1.11	1.34	2.74	1.66	4
Phosphatidylcholine-sterol acyltransferase	-1.36	-1.19	-1.50	-1.70	1.16	1.25	1.13	1.09	4
Phosphatidylinositol 4.5-bisphosphate 5-phosphatase A	-1.26	-1.32	-1.55	-1.46	-1.18	1.13	1.09	1.18	4

Gene Name	Fold-Change								Cluster
	00/24	03/24	06/24	09/24	12/24	15/24	18/21	21/24	
Phosphoinositide-3-kinase-interacting protein 1	-1.22	-1.97	-2.39	-2.22	-1.84	1.19	1.07	1.32	4
Phosphomannomutase 1	-1.16	-1.43	-1.99	-2.33	-2.63	-2.09	-1.57	-1.06	4
Pleckstrin homology-like domain family A member 3	1.14	-1.07	-1.10	-1.44	-1.08	-1.22	1.01	1.20	4
Polymerase delta-interacting protein 3	-1.08	-1.22	-1.24	-1.18	-1.12	1.10	1.08	1.00	4
Polypeptide N-acetylgalactosaminyltransferase 6	-1.05	-1.11	-1.31	-1.08	1.11	1.22	1.55	1.40	4
Post-GPI attachment to proteins factor 3	1.38	1.34	-1.20	-1.35	-1.23	1.15	1.40	1.18	4
Potassium voltage-gated channel subfamily B member 2	-2.34	-1.29	-1.61	-1.23	-1.10	1.09	1.13	1.17	4
Potassium voltage-gated channel subfamily H member 8-like	1.04	-1.22	-1.19	-1.50	-1.34	-1.45	-1.24	1.16	4
PQ-loop repeat-containing protein 2	-1.08	-1.39	-1.66	-1.46	-1.24	1.35	1.52	1.33	4
PR domain zinc finger protein 10	1.09	-1.09	-1.44	-1.46	-1.37	-1.27	-1.11	1.07	4
Pre-mRNA-splicing factor syf2	1.02	-1.14	-1.24	-1.20	-1.18	-1.03	1.01	1.02	4
Probable E3 ubiquitin-protein ligase HERC1	-1.06	-1.04	-1.04	-1.08	1.16	1.17	1.16	1.26	4
Probable E3 ubiquitin-protein ligase makorin-1	-1.25	-1.34	-1.71	-1.83	-1.49	-1.37	-1.31	-1.10	4
Probable E3 ubiquitin-protein ligase MGRN1	-1.05	-1.06	-1.26	-1.11	1.00	1.13	1.15	-1.00	4
Probable G-protein coupled receptor 157	-1.01	-1.12	-1.43	-1.66	-1.51	1.12	1.13	1.14	4
Probable methyltransferase BCDIN3D	-1.04	-1.27	-1.30	-1.29	-1.33	-1.06	1.06	-1.06	4
Probable palmitoyltransferase ZDHHC23	-1.01	-1.04	-1.09	-1.25	-1.05	-1.21	-1.06	1.01	4
Probable phospholipid-transporting ATPase ID	-1.42	-1.03	-1.76	-1.08	1.03	1.24	2.15	2.11	4
Probable polypeptide N-acetylgalactosaminyltransferase 8	1.03	-1.32	-1.53	-1.25	-1.13	1.04	1.23	1.40	4
Probable sodium-coupled neutral amino acid transporter 6	1.06	1.09	-1.37	-1.39	-2.06	-1.09	-1.03	1.04	4
Probable tRNA pseudouridine synthase 2	-1.10	-1.21	-1.33	-1.38	-1.44	-1.38	-1.09	-1.11	4
Progestin and adipoQ receptor family member 6	-1.20	-1.71	-2.03	-1.98	-2.04	-1.37	-1.15	1.10	4
Prolactin receptor	1.45	1.26	-1.10	-1.56	-1.29	-1.15	1.13	1.36	4
Proline-rich protein 15-like protein B	-1.29	-1.25	-2.10	-2.52	-2.39	-1.67	-1.25	1.15	4
Prominin-1	1.39	1.04	-1.32	-1.26	-1.27	1.05	1.65	1.38	4
Pro-neuregulin-1. membrane-bound isoform	-1.15	-1.24	-1.24	-1.21	-1.15	-1.12	1.00	1.03	4
Pro-neuregulin-4. membrane-bound isoform-like	-1.38	-1.29	-1.87	-2.10	-1.16	-1.13	1.27	1.55	4
Prostaglandin E2 receptor EP3 subtype	1.06	-1.09	-1.26	-1.50	-1.40	-1.09	1.39	1.21	4
Protease-associated domain-containing protein 1	-1.01	-1.21	-1.27	-1.21	-1.20	-1.10	-1.16	-1.03	4
Protein C20orf11 homolog	-1.08	-1.26	-1.21	-1.18	-1.08	1.19	1.12	1.16	4
Protein canopy homolog 2	-1.07	1.33	1.23	1.03	1.43	1.42	2.49	1.66	4
Protein CDKN2AIP homolog A	-1.09	-1.08	-1.25	-1.60	-1.30	-1.22	-1.29	-1.08	4
Protein ENL	-1.02	-1.15	-1.33	-1.38	-1.47	-1.21	-1.08	-1.06	4
Protein FAM102A	-1.25	-1.56	-2.14	-2.70	-2.66	-1.99	-1.57	-1.12	4
Protein FAM110C	1.01	-1.49	-1.44	-1.42	-1.16	1.19	1.01	1.09	4
Protein FAM13A	-1.17	-1.48	-1.64	-2.01	-1.81	-1.22	-1.25	-1.00	4
Protein FAM176B	-1.00	-1.23	-1.11	-1.18	-1.03	1.22	1.38	1.28	4
Protein FAM32A	-1.07	-1.22	-1.19	-1.19	-1.03	1.07	1.02	1.03	4
Protein FAM49A	1.05	1.02	-1.37	-1.33	-1.18	-1.08	1.12	-1.02	4

Gene Name	Fold-Change								Cluster
	00/24	03/24	06/24	09/24	12/24	15/24	18/21	21/24	
Protein FAM69A	-1.11	-1.11	-1.38	-1.64	-1.61	-1.32	-1.11	-1.05	4
Protein FAM76B	1.00	-1.03	-1.28	-1.36	-1.31	-1.15	-1.18	-1.01	4
Protein FAM8A1	1.04	-1.11	-1.13	-1.13	-1.13	1.02	1.06	-1.00	4
Protein GTLF3B	-1.08	-1.22	-1.43	-1.19	-1.10	1.29	1.21	1.26	4
Protein ITFG3	1.05	-1.03	-1.08	-1.01	1.15	1.21	1.18	1.10	4
Protein max	-1.13	-1.24	-1.40	-1.34	-1.25	-1.09	-1.07	1.01	4
Protein MEMO1	1.00	-1.09	-1.21	-1.19	-1.06	1.13	1.10	1.02	4
Protein naked cuticle homolog 1	-1.08	1.03	-1.20	1.02	1.18	1.22	1.24	1.18	4
Protein NipSnap homolog 1	-1.07	-1.13	-1.29	-1.51	-1.43	-1.24	-1.06	-1.06	4
Protein strawberry notch homolog 1	1.13	-1.16	-1.52	-1.66	-1.37	-1.22	-1.35	-1.16	4
Protein unc-93 homolog A	1.24	1.16	-1.81	-1.28	-1.07	1.03	1.47	1.59	4
Protein UXT homolog	1.10	-1.10	-1.25	-1.33	-1.24	-1.01	1.02	1.05	4
Protein ycf2	-1.67	-1.14	1.15	-1.09	-1.09	1.26	1.30	1.44	4
Protein YIPF1	-1.07	-1.08	-1.15	1.06	1.02	1.20	1.18	1.14	4
Proto-oncogene c-Rel	-1.15	-1.41	-1.37	-1.56	-1.22	1.16	1.21	1.56	4
Proto-oncogene serine/threonine-protein kinase pim-1	-1.21	-1.28	-1.90	-1.69	-1.23	-1.33	1.36	1.11	4
Putative deoxyribonuclease tatdn3-B	1.03	-1.13	-1.18	-1.16	-1.23	1.05	1.09	1.02	4
Putative homeodomain transcription factor 1	-1.22	-1.26	-1.65	-1.26	-1.30	1.14	1.16	1.10	4
Putative hydroxypyruvate isomerase	-1.11	-1.15	-1.27	-1.44	-1.33	-1.29	-1.16	1.05	4
Putative N-acetylglucosamine-6-phosphate deacetylase	1.15	1.18	-1.55	-1.34	1.00	1.00	1.22	1.10	4
Putative uncharacterized protein CXorf69	-1.03	-1.22	-1.13	-1.04	1.04	1.33	1.17	1.10	4
Rab proteins geranylgeranyltransferase component A 1	-1.05	-1.13	-1.41	-1.22	1.05	1.15	1.33	1.06	4
RAD50-interacting protein 1	1.00	-1.04	-1.16	-1.17	-1.12	-1.08	-1.03	-1.00	4
Ral guanine nucleotide dissociation stimulator	1.12	1.08	-1.21	-1.20	-1.04	-1.09	1.10	1.11	4
Ran-binding protein 9	-1.07	-1.00	-1.13	-1.18	-1.07	1.13	1.42	1.24	4
RanBP-type and C3HC4-type zinc finger-containing protein 1	-1.18	-1.30	-1.60	-1.99	-1.27	-1.26	-1.19	-1.13	4
Rap1 GTPase-activating protein 2	-1.02	-1.17	-1.23	-1.17	-1.10	1.05	-1.03	1.09	4
Ras-associating and dilute domain-containing protein	-2.10	-1.56	-1.68	-1.29	-1.21	-1.47	1.20	1.22	4
Ras-interacting protein 1	-1.04	-1.09	-1.09	-1.08	-1.01	1.18	1.19	1.07	4
Ras-related protein Rab-33B	-1.11	-1.43	-1.72	-2.07	-1.51	1.03	1.06	1.16	4
Ras-related protein Rab-38	1.00	-1.23	-1.29	-1.08	-1.13	-1.02	1.20	1.42	4
Ras-related protein Rab-4B	-1.11	-1.11	-1.67	-2.13	-1.39	-1.20	1.15	1.07	4
Ras-related protein Rab-6B &gt	1.00	-1.16	-1.15	-1.09	-1.07	1.02	1.08	1.06	4
Ras-related protein R-Ras2	1.02	-1.07	-1.12	-1.10	-1.00	1.17	1.22	1.12	4
Receptor expression-enhancing protein 1	-1.23	1.03	-1.34	-1.17	-1.16	1.08	1.83	1.52	4
Retinitis pigmentosa 9 protein homolog	-1.20	-1.36	-1.43	-1.60	-1.49	-1.19	1.01	-1.10	4
Retinoid isomerohydrolase	-1.17	-1.29	-1.23	-1.12	-1.14	1.24	1.15	1.05	4
Rho guanine nucleotide exchange factor 37	-2.27	-1.50	-1.97	-1.07	-1.16	1.85	1.77	1.03	4
Rhomboid domain-containing protein 1	-1.18	-1.14	-1.85	-1.59	-1.46	-1.05	1.77	1.09	4
Rho-related GTP-binding protein RhoU	1.07	-1.21	-1.26	-1.19	1.09	1.47	-1.08	1.07	4
Ribonuclease P protein subunit p40	1.08	-1.43	-1.74	-1.53	-1.93	-1.82	-1.22	1.00	4
Ribonuclease P/MRP protein subunit POP5	-1.25	-1.47	-1.40	-2.01	-1.53	-1.46	-1.31	-1.25	4

Gene Name	Fold-Change								Cluster
	00/24	03/24	06/24	09/24	12/24	15/24	18/21	21/24	
Ribonuclease T2	-1.06	-1.16	-1.21	-1.23	-1.19	1.05	1.07	1.08	4
Ribosomal protein S6 kinase beta-2	-1.15	-1.15	-1.42	-1.55	-1.64	-1.33	-1.07	1.00	4
RING finger protein 114	1.02	-1.15	-1.23	-1.36	-1.24	1.03	1.10	1.15	4
RING finger protein 122	-1.03	-1.20	-1.42	-1.29	-1.10	1.19	1.25	1.21	4
RNA (guanine-9-)-methyltransferase domain-containing protein 3	-1.18	-1.18	-1.40	-1.41	-1.62	-1.36	-1.24	-1.15	4
RNA-binding protein 10	-1.17	-1.23	-1.38	-1.22	-1.15	-1.01	-1.09	-1.06	4
Salivary glue protein Sgs-4	1.01	1.13	1.13	1.19	1.21	1.19	1.58	1.45	4
Scavenger receptor class A member 5	-1.07	-1.16	-1.12	-1.09	-1.01	1.25	1.35	1.15	4
Scm-like with four MBT domains protein 2	-1.12	-1.14	-1.30	-1.48	-1.24	1.05	-1.06	1.06	4
Secreted frizzled-related protein 3	-1.05	-1.12	-1.08	-1.05	-1.17	-1.05	1.36	1.18	4
Semaphorin-4B	1.09	1.11	-1.10	-1.30	-1.16	-1.21	1.05	1.12	4
Sentrin-specific protease 5	-1.27	-1.27	-1.37	-1.46	-1.45	-1.48	-1.17	-1.15	4
Sequestosome-1	-1.08	-1.10	-1.51	-1.60	-1.33	-1.05	1.20	1.10	4
Serine protease hepsin	-1.13	-1.34	-2.55	-2.65	-1.98	-1.37	-1.29	-1.00	4
Serine/arginine-rich splicing factor 10	-1.10	-1.27	-1.32	-1.19	-1.06	1.07	-1.09	1.00	4
Serine/threonine/tyrosine-interacting protein	-1.08	-1.23	-1.28	-1.48	-1.44	-1.43	-1.17	-1.03	4
Serine/threonine-protein kinase 32C	-1.20	-1.09	-1.24	-1.12	1.14	1.15	1.39	1.33	4
Serine/threonine-protein kinase D3	-1.93	-1.64	-2.35	-2.91	-1.86	-1.13	-1.23	1.02	4
Serine/threonine-protein kinase ICK	1.00	-1.06	-1.27	-1.24	-1.00	1.10	1.09	1.04	4
Serine/threonine-protein kinase LMTK1-like isoform X3	-1.09	-1.22	-1.36	-1.59	-1.96	-1.23	-1.09	1.11	4
Serine/threonine-protein kinase Nek3	-1.19	1.15	-1.39	-1.47	-1.08	-1.00	1.38	1.48	4
Serine/threonine-protein kinase pim-2	1.43	-1.16	1.14	-1.21	-1.35	1.47	1.49	1.39	4
Serine/threonine-protein kinase Sgk3	-1.01	-1.07	-1.28	-1.39	-1.36	-1.27	-1.14	1.03	4
Serine/threonine-protein kinase SRPK3	1.08	-1.28	-1.47	-1.67	-1.72	-1.40	-1.19	-1.06	4
Serine/threonine-protein phosphatase 2A regulatory subunit B'' subunit alpha	-1.02	-1.31	-1.20	-1.48	-1.56	-1.07	1.26	-1.10	4
Serine/threonine-protein phosphatase 2A regulatory subunit B'' subunit beta	-1.05	-1.28	-1.75	-2.22	-2.37	-1.69	-1.27	-1.17	4
Sestrin-1	-1.51	-1.50	-1.63	-1.22	-1.11	1.44	1.02	1.15	4
SET and MYND domain-containing protein 3	1.19	-1.15	-1.08	-1.10	-1.30	-1.04	1.21	1.12	4
Short transient receptor potential channel 4-associated protein	-1.08	-1.27	-1.50	-1.44	-1.55	-1.42	-1.37	-1.05	4
SID1 transmembrane family member 2	-1.01	-1.25	-1.67	-1.39	-1.21	-1.01	1.10	1.23	4
Small VCP/p97-interacting protein	-1.04	-1.21	-1.32	-1.19	-1.02	1.29	1.23	1.16	4
Sodium- and chloride-dependent creatine transporter 1	1.29	1.01	-1.12	-1.30	-1.51	-1.14	1.11	1.22	4
Sodium channel modifier 1	-1.17	-1.28	-1.65	-1.42	-1.43	-1.02	1.19	1.09	4
Sodium/bile acid cotransporter-like	-1.01	-1.03	-1.21	-1.45	-1.22	-1.16	1.03	1.10	4
Sodium/calcium exchanger 3-like isoform X3	1.16	-1.15	1.04	1.09	1.11	1.45	1.56	1.39	4
Sodium/potassium/calcium exchanger 6	-1.20	-1.36	-1.44	-1.98	-1.67	-1.50	-1.48	-1.05	4
Sodium/potassium-transporting ATPase subunit beta-233	-1.19	-1.12	-1.14	-1.31	-1.36	-1.32	1.01	1.09	4
Sodium-dependent phosphate transporter 1-A	1.03	1.03	-1.20	-1.29	-1.21	-1.28	1.12	1.03	4
Sodium-dependent phosphate transporter 2	-1.21	-1.39	-2.02	-2.43	-2.25	-2.00	-1.43	1.09	4
Solute carrier family 12 member 8	1.01	1.30	-1.11	-1.76	-1.40	1.22	1.43	1.34	4
Solute carrier family 13 member 1	1.26	1.18	-1.14	-1.14	-1.45	1.49	1.63	1.27	4

Gene Name	Fold-Change								Cluster
	00/24	03/24	06/24	09/24	12/24	15/24	18/21	21/24	
Solute carrier family 15 member 5	-1.12	-1.31	-1.80	1.24	2.11	2.32	3.35	2.45	4
Solute carrier family 25 member 36-A	-1.19	-1.14	-1.59	-1.57	-1.42	-1.17	1.17	1.37	4
Solute carrier family 25 member 43	-1.13	-1.20	-1.55	-1.60	-1.44	1.09	1.35	1.36	4
Solute carrier family 25 member 45	1.17	-1.04	-1.03	1.03	1.03	1.26	1.56	1.35	4
Somatostatin receptor type 2	1.14	-1.09	-1.37	-1.47	1.09	1.04	1.25	1.27	4
Sorting nexin-10	-1.22	-1.52	-1.68	-1.97	-1.73	-1.86	1.05	1.09	4
Sorting nexin-22	-1.04	-1.32	-1.30	-1.37	-1.40	-1.22	-1.37	-1.12	4
Sorting nexin-27	-1.15	-1.27	-1.38	-1.49	-1.53	-1.30	-1.18	-1.01	4
Spastin	1.01	-1.12	-1.06	-1.07	-1.05	1.18	1.18	1.12	4
Sperm-associated antigen 7 homolog	-1.08	-1.23	-1.34	-1.25	-1.27	-1.04	-1.04	-1.04	4
Spermatogenesis-associated protein 2-like protein	1.14	-1.04	-1.27	-1.19	1.02	1.24	1.38	1.28	4
Spermatogenesis-associated protein 5-like protein 1	1.09	-1.21	-1.39	-1.55	-1.56	-1.29	-1.07	1.07	4
Sphingomyelin phosphodiesterase	1.01	-1.07	-1.54	-1.28	-1.17	1.32	1.45	1.24	4
StAR-related lipid transfer protein 13	1.09	-1.07	-1.43	-1.71	-1.49	-1.21	-1.12	1.05	4
Stromal cell-derived factor 1	-1.40	-1.15	-1.52	-1.29	-1.48	-1.02	1.02	1.01	4
Stromelysin-3	1.20	-1.22	-1.20	-1.02	-1.05	1.52	1.05	1.33	4
Superkiller viralicidic activity 2-like 2	1.19	-1.08	-1.63	-1.31	-1.28	1.20	1.14	1.28	4
SWI/SNF-related matrix-associated actin-dependent regulator of chromatin subfamily E member 1-related	-1.11	-1.22	-1.21	-1.17	-1.11	1.05	1.02	-1.09	4
Synapse-associated protein 1	-1.04	-1.34	-1.44	1.06	1.11	1.58	1.11	1.14	4
Synaptotagmin-like protein 1	-1.24	1.05	-1.20	-1.51	1.00	-1.12	1.45	1.69	4
Syntaxin-8	-1.01	1.01	-1.09	-1.00	1.09	1.24	1.17	1.11	4
Target of Myb protein 1	1.00	-1.17	-1.42	-1.26	-1.07	1.16	1.33	1.27	4
TBC1 domain family member 1	-1.10	-1.09	-1.10	-1.15	-1.12	1.07	1.32	1.17	4
TBC1 domain family member 4	-1.25	-1.12	-1.65	-1.80	-1.81	-1.36	-1.11	1.13	4
T-box transcription factor TBX1-like	1.03	-1.07	-1.19	-1.12	1.02	1.06	1.12	1.02	4
T-complex protein 11-like protein 2	1.28	-1.09	-1.68	-2.00	-2.32	-1.24	1.22	1.34	4
Tensin-1	-1.30	1.05	-1.53	-1.81	-2.14	-1.73	-1.18	1.11	4
Tensin-like C1 domain-containing phosphatase-like	-1.02	1.05	-1.44	-1.91	-1.86	-1.37	1.14	1.33	4
Tetraspanin-11	1.41	1.25	-1.27	-1.15	-1.71	-1.03	1.63	1.16	4
Tetraspanin-33	-1.03	-1.19	-1.37	-1.54	-1.18	-1.07	1.03	1.09	4
Tetratricopeptide repeat protein 32	-1.03	-1.20	-1.11	-1.13	-1.01	1.19	1.21	1.09	4
Thioredoxin-interacting protein	-1.79	-1.26	-1.95	-1.91	-1.78	-1.16	1.95	1.09	4
THO complex subunit 4	1.02	-1.13	-1.01	1.04	1.04	1.19	1.25	1.08	4
Thrombospondin-1	-1.31	-2.26	-1.65	-1.39	-1.32	1.24	1.13	1.43	4
Thromboxane-A synthase	1.03	1.12	-1.46	-1.61	-1.62	-1.19	-1.15	-1.06	4
Tissue alpha-L-fucosidase	-1.10	-1.26	-1.38	-1.24	-1.23	1.09	1.06	-1.00	4
TLC domain-containing protein 2	-1.13	-1.28	-1.87	-2.03	-1.70	-1.41	-1.24	-1.03	4
TM2 domain-containing protein 2	-1.15	-1.25	-1.31	-1.25	-1.10	1.03	-1.00	-1.05	4
Toll-like receptor 13	1.12	1.62	1.35	1.13	1.00	1.85	1.54	2.48	4
Trafficking protein particle complex subunit 2-like protein	-1.05	-1.29	-1.20	-1.14	-1.07	1.08	-1.04	-1.02	4
Transcobalamin-2	-1.21	-1.13	-1.03	-1.03	-1.13	1.10	1.24	-1.06	4

Gene Name	Fold-Change								Cluster
	00/24	03/24	06/24	09/24	12/24	15/24	18/21	21/24	
Transcription elongation factor 1 homolog	-1.06	-1.13	-1.14	-1.12	-1.15	1.05	-1.05	1.01	4
Transcription elongation factor A N-terminal and central domain-containing protein 2	-1.16	-1.25	-1.30	-1.19	-1.07	1.10	1.04	1.00	4
Transcription elongation factor A protein 3	1.05	-1.41	-1.87	-2.09	-1.86	-1.21	-1.02	1.09	4
Transcription factor Adf-1	-1.19	-1.12	-1.53	-1.37	-1.45	-1.05	1.24	1.21	4
Transcription factor COE2	-1.30	1.71	-1.28	-1.67	1.02	1.15	1.88	1.87	4
Transcription factor E2F4	1.07	-1.17	-1.26	-1.26	-1.31	1.20	1.09	1.07	4
Transcription factor ETV7	1.08	-1.56	-1.10	-1.03	-1.19	1.20	1.12	1.08	4
Transcription factor HES-1-B	-1.06	-1.15	-1.33	-1.15	1.01	1.07	1.28	1.17	4
Transcription factor HES-4-B	1.05	-1.10	-1.11	1.01	1.08	1.14	1.31	1.03	4
Transcription factor Maf	-1.05	-1.13	-1.15	-1.06	1.02	1.15	1.09	1.10	4
Transcription factor MafB	-1.03	1.01	-1.15	-1.22	1.06	1.03	1.32	1.33	4
Transcriptional regulator Myc	-1.07	-1.40	-1.61	-1.33	1.01	1.35	1.34	1.25	4
Transcriptional regulator Myc-1-like	-1.59	-1.69	-2.76	-3.23	-2.44	-1.64	1.20	1.85	4
Transducin-like enhancer protein 3	1.10	1.03	-1.11	1.01	1.18	1.23	1.33	1.37	4
Transforming acidic coiled-coil-containing protein 1	-1.11	-1.33	-1.67	-2.06	-1.71	-1.64	-1.38	-1.03	4
Transforming acidic coiled-coil-containing protein 2	-1.02	-1.13	-1.24	-1.16	1.08	1.07	-1.01	1.13	4
Transmembrane and coiled-coil domain-containing protein 4	-1.60	-1.62	-2.32	-1.52	-1.26	-1.19	-1.08	-1.12	4
Transmembrane anterior posterior transformation protein 1 homolog	-1.19	-1.29	-1.23	-1.39	-1.47	-1.33	-1.24	1.00	4
Transmembrane protein 150A-like	-1.06	-1.41	-1.54	-1.69	-1.15	1.02	1.15	1.17	4
Transmembrane protein 150B	-1.03	-1.12	-1.63	-1.76	-1.53	-1.11	1.21	1.30	4
Transmembrane protein 174	-1.34	-1.26	-1.63	-2.19	-1.06	1.06	1.78	1.11	4
Transmembrane protein 181	-1.11	-1.15	-1.54	-1.47	-1.33	-1.19	-1.05	-1.01	4
Transmembrane protein 222	-1.37	-1.14	-1.46	-1.12	-1.25	1.03	1.09	-1.06	4
Transmembrane protein 5	1.06	-1.06	-1.30	-1.30	-1.11	1.02	1.05	1.09	4
Transmembrane protein 56-B	1.00	-1.16	-1.25	-1.31	-1.10	1.08	1.08	1.15	4
Transmembrane protein 59	-1.02	-1.16	-1.24	-1.22	-1.14	-1.00	-1.16	1.04	4
Transmembrane protein 82	-1.00	-1.16	-1.42	-1.39	-1.31	1.02	1.16	1.07	4
Transmembrane protein 8A	-1.52	-1.47	-1.38	-1.36	-1.30	1.24	1.74	1.31	4
Transmembrane protein C20orf108	-1.08	-1.05	-1.46	-1.51	-1.44	-1.39	-1.20	1.01	4
Transposase for transposon Tn501	-1.56	-1.06	-1.17	1.21	-1.03	-1.46	1.98	1.14	4
Tripartite motif-containing protein 29	-1.06	-1.12	-1.28	-1.12	1.03	1.34	1.27	1.31	4
Tripartite motif-containing protein 55	-1.08	-1.30	-1.34	-1.08	-1.04	1.74	2.05	1.51	4
Tripartite motif-containing protein 72	1.26	1.15	-1.30	-1.04	-1.89	1.47	2.32	1.74	4
TRMT1-like protein	-1.08	-1.30	-1.16	-1.06	-1.12	1.25	1.15	1.06	4
TRNA (guanine-N(1)-)-methyltransferase	-1.07	-1.13	-1.20	-1.28	-1.30	-1.17	-1.05	-1.10	4
TRNA wybutosine-synthesizing protein 2 homolog	1.15	-1.57	-2.06	-1.14	-1.34	1.40	1.37	1.02	4
TRNA(His) guanylyltransferase	-1.02	-1.17	-1.34	-1.71	-1.60	-1.48	-1.02	-1.05	4
Tubulin polyglutamylase complex subunit 2	1.01	-1.22	-1.65	-1.16	-1.08	-1.07	1.32	1.20	4
Tumor necrosis factor alpha-induced protein 2	1.17	-1.10	-1.33	-1.13	1.19	1.96	1.79	1.14	4
Tumor protein p53-inducible nuclear protein 1	-1.38	-1.67	-2.04	-2.47	-1.49	1.39	1.01	1.42	4
Tumor suppressor candidate 3	1.09	-1.13	-1.15	-1.07	-1.12	1.21	1.18	1.11	4

Gene Name	Fold-Change								Cluster
	00/24	03/24	06/24	09/24	12/24	15/24	18/21	21/24	
Two pore calcium channel protein 1	1.02	1.01	-1.15	-1.09	1.06	1.17	1.14	1.22	4
Two pore calcium channel protein 2	1.03	1.56	1.03	1.50	1.23	1.22	2.38	1.43	4
Type III iodothyronine deiodinase	-1.23	1.50	1.23	-1.06	-1.14	-1.06	1.46	2.12	4
Tyrosine-protein kinase transforming protein RYK	1.16	-1.04	-1.26	-1.35	1.02	1.28	1.55	1.24	4
Tyrosine-protein kinase transforming protein SEA	-1.03	-1.11	-1.25	-1.30	-1.24	-1.24	1.02	-1.02	4
Tyrosine-protein phosphatase non-receptor type 5	1.09	1.32	-1.00	1.21	1.27	1.09	1.61	1.28	4
U11/U12 small nuclear ribonucleoprotein 25 kDa protein	-1.03	-1.20	1.00	1.08	1.04	1.22	1.40	1.10	4
UBA-like domain-containing protein 1	-1.57	-1.94	-2.82	-2.06	-2.03	1.15	1.02	1.27	4
Ubiquitin carboxyl-terminal hydrolase 36	-1.21	-1.50	-1.65	-1.83	-1.77	-1.64	-1.50	-1.03	4
Ubiquitin domain-containing protein 1	1.14	-1.67	-2.87	-2.31	-1.94	-1.79	-1.37	1.31	4
Ubiquitin fusion degradation protein 1 homolog	-1.14	-1.24	-1.27	-1.24	-1.18	1.01	1.06	-1.07	4
Ubiquitin thioesterase OTUB2	-1.01	-1.16	-1.30	-1.40	-1.20	-1.23	-1.14	-1.04	4
Ubiquitin-associated protein 1-like	1.04	-1.78	-1.81	-2.20	-2.34	-1.22	-1.42	1.29	4
Ubiquitin-conjugating enzyme E2 H	-1.33	-1.38	-1.51	-1.93	-1.36	-1.21	1.37	1.51	4
Ubiquitin-like protein 3	-1.12	1.13	-1.42	-1.49	-1.25	-1.12	1.27	1.54	4
UDP-GlcNAc:betaGal beta-1.3-N-acetylglucosaminyltransferase 2	-1.29	1.02	-1.14	-1.19	-1.34	1.03	1.28	1.46	4
UDP-GlcNAc:betaGal beta-1.3-N-acetylglucosaminyltransferase 5B	-1.44	1.46	1.13	1.03	1.25	2.47	2.48	2.06	4
UDP-glucuronosyltransferase 2A1	1.07	1.32	-1.25	1.16	1.43	1.48	2.04	1.55	4
UDP-glucuronosyltransferase 2A3-like	1.06	-1.07	-1.27	-1.58	-1.70	-1.34	1.23	1.40	4
UDP-glucuronosyltransferase 2B16	1.08	-1.06	-1.07	-1.38	-1.38	-1.31	1.16	1.26	4
Uncharacterized protein C10orf18 homolog	-1.11	-1.04	-1.27	-1.39	-1.34	-1.35	-1.05	-1.10	4
Uncharacterized protein C11orf51 homolog	1.11	-1.10	-1.35	-1.51	-1.05	1.31	1.08	1.01	4
Uncharacterized protein C12orf41 homolog	-1.12	-1.26	-1.69	-1.84	-1.70	-1.30	-1.13	-1.14	4
Uncharacterized protein C14orf43	-1.02	-1.01	-1.32	-1.13	-1.05	1.05	1.14	1.12	4
Uncharacterized protein C15orf57	-1.08	-1.19	-1.11	-1.00	-1.03	1.21	1.12	1.04	4
Uncharacterized protein C17orf90 homolog	1.04	-1.13	-1.17	-1.10	1.04	1.26	1.18	1.07	4
Uncharacterized protein C1orf131 homolog	-1.17	-1.36	-1.40	-1.58	-1.86	-1.61	-1.25	-1.16	4
Uncharacterized protein C1orf51 homolog	-1.09	-1.70	-2.17	-2.33	-1.73	-1.69	-1.41	1.44	4
Uncharacterized protein C20orf111 homolog	1.02	-1.15	-1.42	-1.51	-1.60	-1.20	-1.26	-1.17	4
Uncharacterized protein C2orf42 homolog	-1.12	-1.23	-1.51	-1.61	-1.40	-1.27	-1.13	-1.19	4
Uncharacterized protein C3orf18 homolog	1.13	-1.02	-1.09	-1.05	1.17	1.25	1.34	1.19	4
Uncharacterized protein C3orf54 homolog	1.13	-1.27	-1.20	-1.08	-1.08	1.27	-1.09	-1.05	4
Uncharacterized protein C4orf34 homolog	1.03	1.01	-1.23	-1.35	-1.39	-1.34	-1.08	1.07	4
Uncharacterized protein C6orf64	-1.12	-1.06	-1.07	-1.06	-1.03	1.14	1.28	1.13	4
Uncharacterized protein C7orf30	-1.14	-1.23	-1.35	-1.35	-1.31	-1.07	1.00	1.01	4
Uncharacterized protein C8orf4 homolog	-1.08	-1.10	-1.37	-1.91	-1.48	-1.20	1.04	-1.16	4
Uncharacterized protein C8orf59	-1.02	-1.44	-1.58	-1.57	-1.86	-1.71	-1.19	-1.07	4
Uncharacterized protein C8orf59 homolog	-1.16	-1.52	-1.77	-1.84	-1.88	-1.81	-1.33	-1.10	4
Uncharacterized protein C9orf25	-1.32	1.06	-1.17	-1.07	-1.00	1.04	1.34	1.33	4
Uncharacterized protein ENSP00000370281 homolog	-1.08	-1.25	-1.86	-1.97	-1.21	1.14	-1.01	1.12	4

Gene Name	Fold-Change								Cluster
	00/24	03/24	06/24	09/24	12/24	15/24	18/21	21/24	
Uncharacterized protein KIAA0247	-1.12	-1.21	-1.18	-1.10	-1.00	1.06	-1.00	-1.05	4
Uncharacterized protein KIAA1539 homolog	-1.04	-1.32	-1.60	-1.49	-1.33	1.15	1.46	1.57	4
Uncharacterized protein LOC101218508. partial	-1.74	1.21	-1.42	-1.63	-1.20	-1.77	2.01	1.30	4
UPF0405 protein C3orf75 homolog	-1.00	-1.06	-1.29	-1.30	-1.50	-1.34	-1.04	-1.1	4
UPF0420 protein C16orf58 homolog	-1.01	-1.22	-1.19	-1.11	-1.03	1.15	1.27	1.03	4
UPF0444 transmembrane protein C12orf23 homolog	-1.04	-1.06	-1.17	-1.13	1.02	1.28	1.15	1.02	4
UPF0468 protein C16orf80 homolog	-1.06	-1.09	-1.14	-1.11	-1.03	1.11	1.09	1.01	4
UPF0585 protein C16orf13 homolog B	1.08	-2.16	-2.03	-2.37	2.06	2.87	1.85	2.27	4
UPF0600 protein C5orf51 homolog	-1.04	-1.13	-1.34	-1.48	-1.57	-1.41	-1.12	-1.03	4
UPF0697 protein C8orf40 homolog	-1.25	-1.02	-1.44	-1.11	1.10	1.37	1.31	1.30	4
UPF0711 protein C18orf21 homolog	-1.29	-1.71	-1.56	-1.68	-1.39	1.19	1.18	1.33	4
Upstream stimulatory factor 1	1.13	-1.01	-1.03	1.03	1.09	1.25	1.16	1.15	4
Upstream stimulatory factor 2	-1.28	-1.23	-1.49	-1.52	-1.35	-1.21	1.05	1.15	4
Urokinase-type plasminogen activator	-1.24	-1.59	-1.45	-1.92	-1.50	1.42	1.81	1.50	4
Uroporphyrinogen-III synthase	-1.08	-1.32	-1.41	-1.60	-1.30	-1.16	-1.14	-1.11	4
Vacuolar protein-sorting-associated protein 36	-1.02	-1.12	-1.12	-1.03	1.07	1.16	1.22	1.06	4
Vasoactive intestinal polypeptide receptor 2	1.06	1.11	-1.03	1.03	1.17	1.28	1.60	1.19	4
Vesicle transport protein SFT2A	1.04	-1.04	-1.09	-1.12	-1.12	-1.01	1.06	1.14	4
Vesicle transport through interaction with t-SNAREs homolog 1A	-1.02	-1.19	-1.19	-1.07	1.00	1.11	-1.01	1.05	4
Villin-like protein	1.18	-1.85	-2.30	-2.92	-1.86	-2.62	-1.16	-1.01	4
Von Hippel-Lindau disease tumor suppressor	-1.12	-1.16	-1.18	1.00	1.01	1.18	1.09	1.06	4
V-type proton ATPase 116 kDa subunit a isoform 4	-1.10	-1.13	-1.18	-1.10	-1.05	1.18	1.17	1.07	4
WD repeat domain phosphoinositide-interacting protein 4	-1.06	-1.09	-1.46	-1.49	-1.21	1.05	1.17	1.11	4
WD repeat. SAM and U-box domain-containing protein 1	-1.73	-1.21	-1.87	-1.11	-1.77	1.20	2.53	1.46	4
WD repeat-containing protein 41	1.06	-1.09	-1.56	-1.26	-1.05	1.06	1.24	1.13	4
WD repeat-containing protein 69	-1.06	1.29	-1.11	-1.35	-1.07	-1.17	1.25	1.11	4
WD repeat-containing protein 85	-1.12	-1.22	-1.54	-1.70	-1.68	-1.33	-1.15	-1.17	4
Wolframin	1.07	-1.25	-1.99	-2.12	-1.60	-1.44	1.17	1.09	4
Y+L amino acid transporter 1	1.25	1.12	-1.27	-1.04	1.10	1.15	1.71	1.54	4
YY1-associated factor 2	-1.04	-1.04	-1.08	-1.02	1.08	1.29	1.36	1.34	4
Zinc finger FYVE domain-containing protein 19	1.14	-1.01	-1.08	-1.04	1.04	1.28	1.28	1.01	4
Zinc finger FYVE domain-containing protein 9	-1.57	1.18	-1.36	-1.49	-1.34	-1.29	1.34	1.52	4
Zinc finger HIT domain-containing protein 3	-1.04	-1.28	-1.36	-1.54	-1.44	-1.43	-1.20	-1.14	4
Zinc finger matrin-type protein 1	-1.21	-1.10	-1.24	-1.02	-1.03	1.30	1.38	1.31	4
Zinc finger MYM-type protein 4	1.01	-1.42	-1.61	-1.47	-1.53	-1.20	-1.08	1.18	4
Zinc finger protein 157	1.09	-1.05	-1.48	-1.59	-1.68	-1.28	-1.14	-1.00	4
Zinc finger protein 322	-3.20	-1.25	-1.23	-1.48	-1.24	1.23	1.11	1.27	4
Zinc finger protein 323	-1.09	-1.34	-1.38	-1.74	-1.65	-1.01	-1.14	1.43	4
Zinc finger protein 335	-1.04	-1.14	-1.16	-1.18	-1.22	-1.11	-1.14	-1.07	4
Zinc finger protein 431	-1.09	-1.26	-1.31	-1.12	-1.03	1.46	1.18	-1.02	4
Zinc finger protein 630	-1.02	-1.26	-1.19	-1.00	1.07	1.17	1.08	1.00	4

Gene Name	Fold-Change								Cluster
	00/24	03/24	06/24	09/24	12/24	15/24	18/21	21/24	
Zinc finger protein 82	-1.07	-1.23	-1.29	-1.15	-1.07	1.34	1.24	-1.07	4
Zinc transporter ZIP8	-1.17	-1.37	-1.91	-1.64	-1.60	-1.16	1.23	1.19	4
Zinc transporter ZIP8-like	-1.16	-1.26	-2.12	-1.63	-1.39	-1.12	1.29	1.18	4

Supplementary Table S2. Significantly correlated genes (Spearman correlation coefficient, P< 0.05) to reference clock genes included in cluster1 (*per3*), cluster 2 (*per1*, *per2*, *cry1*, *cry2*) and cluster 3 (*bmal1*, *clock*). Normalized intensity values are shown for each gene and sampling time.

Clone	Similar entities	00:00	03:00	06:00	09:00	12:00	15:00	18:00	21:00	24:00
	CLUSTER 1 entities similar to per3									
C2_441	60S ribosomal protein L13a	0.30	0.21	-0.49	-0.49	-0.90	-0.42	0.18	0.45	0.84
C2_4364	Alpha-crystallin B chain	1.51	0.94	-0.58	-2.09	-3.02	-2.65	-0.33	1.11	1.74
C2_5212	Angiopoietin-related protein 7	0.61	-0.02	-0.50	-0.69	-0.90	-0.42	-0.01	0.51	0.54
C2_1538	Angiotensin-converting enzyme 2	0.64	0.27	-0.26	-0.54	-0.75	-0.36	-0.10	0.55	0.31
C3_c23026	Collagen alpha-3(VI) chain-like	0.63	0.12	-0.15	-0.57	-1.01	-0.30	-0.22	0.62	0.77
C2_5711	Cytidine deaminase	0.38	0.23	-0.26	-1.49	-0.97	-0.66	-0.12	0.51	0.74
C3_c37865	Epidermal growth factor receptor kinase substrate 8-like protein 1-like isoform X2	0.65	0.29	-0.74	-0.92	-0.76	-0.28	-0.04	0.42	0.76
C2_2657	Eukaryotic translation initiation factor 4E-binding protein 3-like	0.64	0.32	-0.34	-0.83	-0.88	-0.42	-0.07	0.58	0.90
C2_14211	Hyaluronan synthase 1	1.00	0.81	-0.52	-1.09	-2.94	-1.59	0.02	0.54	0.92
C2_107323	Hypoxia-inducible factor 3-alpha	0.51	0.26	-0.05	-0.87	-0.55	-0.32	0.02	0.39	0.91
C2_2258	Ileal sodium/bile acid cotransporter	0.87	0.50	-0.74	-0.96	-1.67	-0.95	-0.11	0.36	0.88
C2_14363	Large neutral amino acids transporter small subunit 4	0.58	0.36	-0.54	-0.58	-1.00	-0.52	0.06	0.42	0.51
C3_c30347	LIM domain only protein 7-like	0.33	0.31	-0.38	-0.79	-0.81	-0.57	0.20	0.29	0.32
C2_1137	L-threonine 3-dehydrogenase, mitochondrial	1.33	0.70	-0.47	-1.24	-1.38	-1.51	-0.29	0.94	1.50
C2_14996	Lysyl oxidase homolog 2	0.62	0.12	-0.24	-0.53	-1.05	-0.79	-0.23	0.55	0.70
C2_22601	Mitochondrial uncoupling protein 3 (UCP3)	1.11	0.15	-0.39	-1.28	-1.35	-0.22	-0.09	0.93	1.21
C2_29219	Monocarboxylate transporter 12-B	0.76	0.26	-0.94	-1.68	-1.76	-0.73	0.15	0.98	1.06
C2_1987	Mucolipin-2	0.87	0.42	-0.61	-0.71	-1.08	-0.35	-0.10	0.77	0.86
C2_50960	Na(+)/H(+) exchange regulatory cofactor NHE-RF4	0.38	0.37	-0.39	-0.80	-0.96	-0.72	-0.03	0.44	0.43
C2_24502	Nef-associated protein 1	0.49	0.29	-0.47	-1.19	-0.67	-0.49	0.22	0.35	0.62
C2_44391	Period circadian protein homolog 3	1.41	1.06	-0.64	-1.07	-1.10	-0.92	-0.09	1.22	1.64
C2_4596	Prostaglandin E synthase	0.61	0.49	-0.04	-0.83	-0.96	-0.67	-0.26	0.32	0.72
C2_12452	Protein phosphatase 1K, mitochondrial	0.42	0.41	-0.37	-0.62	-0.65	-0.52	-0.21	0.40	0.73
C2_2559	Protein Tob1	0.73	0.18	-0.44	-1.03	-1.07	-0.28	0.18	0.67	0.91
C2_8183	S-adenosylmethionine decarboxylase proenzyme	0.80	0.38	-0.23	-0.71	-0.91	-0.99	-0.09	0.69	1.05
C2_8696	Sarcospan	0.99	0.12	-0.30	-0.38	-0.63	-0.59	-0.01	0.53	0.60
C2_81766	SH2 domain-containing protein 3C	0.48	0.19	-0.38	-0.40	-1.73	-0.47	-0.25	0.16	0.64
C2_55470	Sorting nexin-16	0.81	0.63	-0.63	-0.80	-1.17	-0.67	-0.36	0.43	0.75
C2_34685	Uncharacterized protein C14orf118 homolog	0.78	0.50	-0.84	-1.87	-1.76	-1.01	-0.16	1.10	1.43
C2_552	Uridine phosphorylase 2	1.06	0.91	-0.29	-1.41	-2.24	-1.41	-0.23	0.29	1.40
	CLUSTER 2 entities similar to per1 or per2, or cry1 or cry2									
C2_4902	17-beta-hydroxysteroid dehydrogenase 14	0.30	0.33	0.25	0.00	-0.11	-0.22	-0.18	0.03	0.08
C2_13398	2-methoxy-6-polyprenyl-1,4-benzoquinol methylase, mitochondrial	0.11	0.32	0.28	0.03	-0.26	-0.28	-0.14	-0.27	0.15

Clone	Similar entities	00:00	03:00	06:00	09:00	12:00	15:00	18:00	21:00	24:00
C2_845	60S ribosomal protein L7-like 1	0.23	0.29	0.33	-0.06	-0.41	-0.75	-0.24	-0.28	0.21
C2_9564	Acyl-CoA-binding domain-containing protein 4	0.50	0.65	0.76	0.46	-0.33	-0.62	-0.36	-0.35	-0.05
C2_1101	Adrenodoxin, mitochondrial	0.15	0.32	0.27	-0.02	-0.11	-0.33	-0.12	-0.12	0.03
C2_45870	Amidophosphoribosyltransferase	0.38	0.51	0.11	-0.25	-0.42	-0.58	-0.27	-0.10	0.01
C2_669	Ammonium transporter Rh type A	0.15	0.21	0.19	0.07	-0.30	-0.35	-0.49	-0.25	0.16
C2_23922	Angiopoietin-related protein 1	0.41	0.28	0.45	-0.03	-0.21	-0.35	-0.29	-0.06	0.15
C2_6122	ATP-dependent zinc metalloprotease YME1L1	0.52	0.82	0.31	-0.04	-0.67	-0.85	-0.65	-0.27	0.39
C3_c27904	Beta-tectorin	0.17	0.79	0.40	-0.11	-0.73	-1.34	-0.23	0.26	0.28
C3_lrc16146	Beta-tectorin-like	0.34	0.49	0.28	-0.22	-0.39	-0.69	-0.39	0.06	0.13
C2_81283	Calcium-binding mitochondrial carrier protein SCaMC-2-A	0.00	0.90	0.65	0.43	-0.51	-1.63	-0.84	-0.05	0.47
AM964132	Chromosome transmission fidelity protein 18 homolog	0.43	0.55	0.76	0.15	-0.27	-0.49	-0.26	-0.18	-0.08
C2_3499	Chromosome transmission fidelity protein 8 homolog	0.22	0.38	0.38	0.06	-0.37	-0.44	-0.15	-0.15	0.23
C2_31305	Coiled-coil domain-containing protein 86	0.14	0.14	0.28	-0.02	-0.34	-0.61	-0.27	-0.18	0.26
C2_8882	Cryptochrome DASH	0.20	0.58	0.37	-0.03	-0.60	-0.71	-0.55	-0.44	0.17
C2_13248	Cryptochrome-1	0.87	1.51	0.96	-0.09	-0.66	-0.86	-0.84	-0.54	0.78
C2_63385	Cryptochrome-2	0.63	1.48	0.79	-0.26	-0.69	-0.99	-0.43	-0.33	0.64
C2_2620	Cystathionine gamma-lyase	0.20	0.36	0.14	-0.10	-0.34	-0.57	-0.19	-0.17	0.22
C2_21983	Cytochrome P450 2B4	0.19	0.55	0.17	0.28	0.00	-0.56	-0.44	-0.21	0.43
C2_3999	Dehydrogenase/reductase SDR family member 1	0.41	0.94	0.65	-0.03	-0.22	-1.03	-0.75	-0.51	0.26
C3_c34861	Dehydrogenase/reductase SDR family member 13-like	0.44	0.69	0.31	-0.11	-0.50	-0.85	-0.95	-0.16	0.27
C3_c35186	Diphthine synthase-like	0.12	0.26	0.26	-0.14	-0.23	-0.62	-0.47	-0.23	0.18
FM148633	DNA mismatch repair protein Msh2	0.45	0.56	0.62	0.20	-0.43	-0.87	-0.33	-0.30	-0.02
C2_10309	DNA polymerase delta catalytic subunit	0.55	0.57	0.58	-0.10	-0.47	-0.71	-0.33	-0.02	0.18
C2_17954	DNA polymerase epsilon subunit 2	0.22	0.30	0.38	-0.10	-0.74	-0.71	-0.25	-0.13	0.02
C2_5648	DNA replication complex GINS protein PSF1	0.23	0.28	0.63	0.22	-0.26	-0.34	-0.20	-0.19	0.13
C2_3706	DNA-directed RNA polymerases I and III subunit RPAC1	0.12	0.13	0.19	-0.03	-0.26	-0.55	-0.06	-0.11	0.10
C2_9286	F-box/LRR-repeat protein 14	0.08	0.18	0.05	0.01	-0.08	-0.25	-0.25	-0.07	-0.03
C2_20304	Gem-associated protein 4	0.11	0.27	0.25	-0.05	-0.07	-0.33	-0.16	-0.14	0.05
FP338940	Glutamate-rich WD repeat-containing protein 1	0.31	0.33	0.37	-0.01	-0.29	-0.76	-0.16	-0.22	0.18
C2_10333	H/ACA ribonucleoprotein complex subunit 3	0.15	0.16	0.21	0.07	-0.33	-0.62	-0.29	-0.17	0.18
C2_12708	Heme oxygenase 2	0.21	0.30	0.29	-0.08	-0.29	-0.41	-0.45	-0.21	0.28
C2_20564	High affinity cationic amino acid transporter 1	0.25	0.54	0.68	0.23	-0.90	-1.56	-1.49	-0.97	0.33
C3_lrc19727	Homeobox protein Hox-A2a	0.16	0.33	0.06	0.00	-0.20	-0.23	-0.12	-0.11	0.10
C2_2104	Homologous-pairing protein 2 homolog	0.25	0.49	0.42	0.07	-0.53	-0.50	-0.19	-0.16	0.15
C2_54399	Inositol-3-phosphate synthase 1-A	0.40	0.70	0.26	0.02	-0.70	-1.26	-0.76	-0.38	0.26
C2_32646	Kelch domain-containing protein 4	0.16	0.18	0.21	-0.02	-0.23	-0.27	-0.13	-0.07	0.13
AM971218	Keratin, type II cytoskeletal	0.74	0.79	0.34	0.12	-0.27	-0.74	-1.28	-0.63	0.16

Clone	Similar entities	00:00	03:00	06:00	09:00	12:00	15:00	18:00	21:00	24:00
C2_32804	Leucine-rich repeat-containing protein 58	0.30	0.50	0.08	0.24	-0.80	-0.52	-0.19	-0.28	0.48
C2_64757	Low affinity vacuolar monovalent cation/H(+) antiporter	0.14	0.21	0.18	-0.08	-0.26	-0.13	-0.09	-0.09	0.14
C2_5997	LysM and putative peptidoglycan-binding domain-containing protein 3	0.22	0.27	0.12	-0.09	-0.20	-0.39	-0.38	-0.10	0.15
C2_12578	Meiotic nuclear division protein 1 homolog	0.34	0.56	0.61	0.20	-0.61	-0.64	-0.61	-0.52	0.12
C2_93588	Mitogen-activated protein kinase kinase kinase 6	0.27	0.36	0.27	-0.15	-0.49	-0.59	-0.73	-0.17	0.19
C2_26959	MOSC domain-containing protein 2, mitochondrial	0.25	0.58	0.32	-0.40	-0.82	-1.04	-0.75	-0.10	0.51
C2_16487	Myb-related protein B	0.55	0.64	0.62	0.31	-0.46	-0.61	-0.28	-0.13	0.14
C2_3594	Myeloid-associated differentiation marker homolog	0.19	0.34	0.28	-0.06	-0.37	-0.42	-0.57	-0.19	0.12
C2_3013	N-acetyltransferase 10	0.08	0.08	0.25	0.04	-0.24	-0.69	-0.07	-0.20	0.07
C2_59500	NIPA-like protein	0.23	0.26	0.47	0.14	-0.27	-0.34	-0.29	-0.18	0.03
C2_79893	Nuclear pore complex protein Nup155	0.22	0.35	0.39	0.03	-0.32	-0.62	-0.98	-0.31	0.17
C2_53107	Nuclear receptor ROR-gamma	0.45	0.71	0.33	-0.05	-0.56	-1.08	-1.32	-0.29	0.30
C2_47345	Origin recognition complex subunit 4	0.33	0.38	0.48	0.08	-0.57	-0.87	-0.29	-0.18	0.01
C2_34563	Origin recognition complex subunit 5	0.32	0.36	0.43	-0.05	-0.56	-0.70	-0.40	-0.20	0.33
C2_21343	Period circadian protein homolog 1	0.24	0.67	0.08	0.26	-0.19	-1.26	-1.20	-0.80	0.33
C2_58904	Period circadian protein homolog 2	0.55	1.44	0.14	0.25	-0.47	-0.85	-0.18	-0.24	0.63
C2_9364	Phosphoglycolate phosphatase	0.03	0.21	0.13	0.04	-0.20	-0.31	-0.12	-0.08	0.14
C2_26487	Probable ATP-dependent RNA helicase DDX11	0.59	0.74	0.65	0.10	-0.48	-0.51	-0.34	-0.27	0.23
C2_3861	Probable ATP-dependent RNA helicase DDX27	0.14	0.21	0.16	-0.05	-0.22	-0.49	-0.16	-0.14	0.23
C2_10438	Protein arginine N-methyltransferase 7	0.10	0.19	0.20	0.06	-0.14	-0.39	-0.17	-0.17	0.06
C2_6748	Protein SDA1 homolog	0.28	0.41	0.27	-0.09	-0.37	-0.77	-0.39	-0.21	0.27
C2_39921	Protein-glutamine gamma-glutamyltransferase 2	0.48	0.55	0.76	0.03	-0.27	-1.38	-1.27	-0.73	0.27
C2_55059	Retinal cone rhodopsin-sensitive cGMP 3',5'-cyclic phosphodiesterase subunit gamma	0.40	0.84	0.85	0.01	-0.58	-1.27	-2.92	-0.97	0.03
C3_c26523	Retinal rod rhodopsin-sensitive cGMP 3',5'-cyclic hosphodiesterase subunit gamma-like	0.33	0.61	0.36	-0.16	-0.42	-0.73	-1.46	-0.13	0.05
C2_8888	Ribosomal RNA processing protein 1 homolog A	0.12	0.15	0.28	0.01	-0.25	-0.69	-0.20	-0.11	0.28
C2_15467	Ribosome biogenesis protein BMS1 homolog	0.05	0.14	0.12	0.12	-0.15	-0.38	-0.18	-0.15	-0.01
C2_88702	Ribosome biogenesis protein bop1	0.12	0.27	0.34	0.03	-0.25	-0.51	-0.39	-0.18	0.13
C2_972	S-adenosylmethionine synthase isoform type-2	0.47	0.48	0.65	-0.01	-0.49	-1.10	-0.49	-0.31	0.16
C2_13518	Scavenger receptor class B member 1	0.10	0.31	0.08	-0.06	-0.39	-0.58	-0.11	-0.13	0.10
C2_18487	Serine/threonine-protein kinase SBK2	0.04	0.70	0.50	0.16	-0.63	-1.19	-1.15	-0.48	0.36
C2_10464	Sodium-coupled neutral amino acid transporter 3	0.38	0.58	0.46	0.16	-0.21	-0.47	-0.51	-0.33	0.32

Clone	Similar entities	00:00	03:00	06:00	09:00	12:00	15:00	18:00	21:00	24:00
C2_7004	Sterol 26-hydroxylase, mitochondrial	0.30	0.46	0.32	-0.01	-0.56	-0.64	-0.49	0.08	0.11
C2_1531	Succinate dehydrogenase assembly factor 2, mitochondrial	0.24	0.58	0.23	0.03	-0.23	-0.37	-0.35	-0.33	0.17
C2_14036	Transducin beta-like protein 3	0.15	0.17	0.41	0.03	-0.30	-0.76	-0.24	-0.10	0.19
C2_60	Translocator protein	0.23	0.67	0.32	0.15	0.03	-0.32	-0.08	-0.14	0.23
C2_2896	Transmembrane protein 120A	-0.03	0.26	0.11	0.14	-0.18	-0.32	-0.23	-0.14	0.22
C2_3540	Tyrosine aminotransferase	0.75	1.38	0.75	-0.11	-1.07	-2.00	-1.16	-0.74	0.87
C2_21536	Ubiquitin carboxyl-terminal hydrolase 9	0.13	0.44	0.33	0.09	-0.13	-0.68	-0.74	-0.59	0.08
C2_984	Uncharacterized methyltransferase WBSCR22	0.13	0.17	0.17	-0.08	-0.32	-0.43	-0.14	-0.12	0.15
C2_2790	Uncharacterized protein At5g50100, mitochondrial	0.22	0.71	0.41	-0.21	-0.62	-0.96	-0.90	-0.44	0.35
C2_2753	Uncharacterized protein C1orf50 homolog	0.14	0.56	0.50	-0.07	-0.32	-0.41	-0.29	-0.14	0.43
C3_c6790	Uncharacterized protein LOC101166720	0.40	1.23	0.57	-0.16	-0.34	-0.81	-0.76	0.10	0.04
C2_21008	WD repeat and HMG-box DNA-binding protein 1	0.53	0.59	0.76	0.39	-0.51	-0.63	-0.36	-0.33	0.02
	CLUSTER 3 entities similar to bmal1 or clock									
C2_13557	6-phosphofructo-2-kinase/fructose-2,6-biphosphatase 2	-0.52	-0.07	0.21	0.53	0.57	0.16	0.12	-0.40	-0.43
C2_4969	6-phosphofructokinase, liver type	-0.26	0.00	0.04	0.22	0.26	0.14	-0.10	-0.29	-0.23
C2_14888	78 kDa glucose-regulated protein	-0.71	-0.22	0.21	0.76	0.53	0.22	0.22	-0.44	-0.31
C2_4392	Adenosine 3'-phospho 5'-phosphosulfate transporter 2	-0.18	-0.10	0.11	0.20	0.21	0.15	0.03	-0.16	-0.08
C2_3814	Aldehyde dehydrogenase family 9 member A1	-0.85	-0.28	0.38	0.52	0.48	0.17	-0.19	-0.65	-0.71
C2_8898	Apoptosis facilitator Bcl-2-like protein 14	-0.75	0.05	0.58	0.94	0.62	0.43	-0.28	-1.04	-0.70
C2_16398	Aryl hydrocarbon receptor nuclear translocator-like protein 1 (BMAL1)	-1.94	0.12	0.61	1.10	0.98	0.24	-0.38	-1.20	-0.88
C2_43110	Bcl-2-like protein 12	-0.51	-0.26	-0.07	0.26	0.52	0.24	0.04	-0.68	-0.33
C2_73896	Beta-citryl-glutamate synthase B	-0.40	0.20	0.59	0.90	0.66	-0.04	-0.26	-0.36	-0.16
C2_22008	Cip1-interacting zinc finger protein	-0.15	-0.02	0.01	0.03	0.19	0.01	0.00	-0.17	-0.16
C2_26384	Circadian locomoter output cycles protein kaput	-1.08	-0.28	0.28	0.95	1.08	0.63	-0.22	-1.08	-0.68
C2_18271	Coenzyme Q-binding protein COQ10 homolog B, mitochondrial	-1.27	-0.48	0.18	0.55	0.78	0.59	0.04	-0.52	-0.99
C3_c46150	Cyclin-dependent kinase 17	-0.29	-0.10	0.06	0.33	0.27	0.16	-0.08	-0.17	-0.23
C2_1055	Cyclin-dependent kinase inhibitor 1	-2.06	-0.56	0.90	1.61	2.00	1.61	-0.19	-2.29	-1.69
C2_2270	Cytosolic sulfotransferase 3	-0.29	0.27	0.36	0.60	0.57	0.05	-0.26	-0.49	-0.21
C2_1362	DCN1-like protein 5	-0.20	-0.10	0.16	0.24	0.23	0.00	0.02	-0.14	-0.13
C2_41762	Dimethylglycine dehydrogenase, mitochondrial	-0.55	0.07	-0.02	0.54	0.65	0.32	0.29	-0.52	-0.50
C2_4669	E3 ubiquitin-protein ligase ZNRF2	-0.37	0.00	0.48	0.94	1.09	0.82	-0.23	-0.54	-0.36
C2_79423	FERM, RhoGEF and pleckstrin domain-containing protein 1	-0.24	-0.09	0.11	0.15	0.21	0.15	0.06	-0.19	-0.37
C2_7621	Four and a half LIM domains protein 3	-0.55	-0.30	0.06	0.25	0.51	0.48	0.20	-0.36	-0.24
C2_3007	Gametogenetin-binding protein 2	-0.24	0.00	0.17	0.26	0.32	0.11	-0.10	-0.20	-0.19
C2_23348	Histone-lysine N-methyltransferase EHMT2	-0.17	-0.09	0.08	0.24	0.29	0.14	-0.09	-0.22	-0.15
C2_83951	Homer protein homolog 1	-0.65	-0.09	0.14	0.36	0.31	0.26	0.09	-0.28	-0.41

Clone	Similar entities	00:00	03:00	06:00	09:00	12:00	15:00	18:00	21:00	24:00
C2_8172	Immunoglobulin superfamily member 5	-0.59	-0.11	0.03	0.32	0.47	0.35	0.30	-0.62	-0.57
C2_5889	Kidney mitochondrial carrier protein 1	-0.54	-0.15	0.75	0.79	0.92	0.42	-0.11	-0.78	-0.44
C2_16722	Methylosome subunit pICln	-0.19	0.00	0.18	0.19	0.12	0.08	-0.11	-0.25	-0.19
C2_2814	Motile sperm domain-containing protein 2	-0.46	-0.13	0.24	0.47	0.47	0.44	0.00	-0.36	-0.33
C2_81334	Mucin-3A	-0.26	-0.12	0.21	0.44	0.31	0.14	0.07	-0.18	-0.08
C2_16468	Nuclear pore complex protein Nup93	-0.37	-0.13	0.39	0.45	0.15	0.02	-0.06	-0.30	-0.25
C2_22441	Probable ATP-dependent RNA helicase YTHDC2	-0.31	-0.21	0.50	0.60	0.20	0.16	-0.11	-0.23	-0.29
C2_54553	Protein deltex-1	-0.38	-0.23	-0.03	1.49	2.47	0.60	-0.07	-0.38	-0.42
C2_729	RNA polymerase II subunit A C-terminal domain phosphatase SSU72	-0.16	-0.15	0.07	0.21	0.22	0.14	0.05	-0.16	-0.18
C2_48154	Serine incorporator 3	-0.72	0.07	0.29	1.05	0.66	0.12	-0.11	-0.23	-0.38
C2_47279	Solute carrier family 25 member 36	-1.03	-0.39	0.59	1.64	1.59	0.68	-0.14	-0.56	-0.82
C2_33191	SPRY domain-containing SOCS box protein 3	-0.51	-0.02	0.01	0.47	0.55	0.36	0.04	-0.29	-0.42
C2_5569	Transmembrane protein 214-A	-0.58	-0.23	0.15	0.43	0.43	0.31	0.10	-0.55	-0.52
C2_2326	Tumor necrosis factor receptor type 1-associated DEATH domain protein	-0.43	-0.12	0.18	0.34	0.46	0.33	0.08	-0.37	-0.42
C2_22550	UHRF1-binding protein 1-like	-0.21	-0.15	0.13	0.20	0.37	0.20	-0.15	-0.17	-0.25
C2_5518	Uncharacterized protein C7orf36	-0.20	-0.07	0.10	0.20	0.12	0.07	-0.09	-0.14	-0.03
C3_c8931	Uncharacterized protein LOC101469315 isoform X1	-1.58	-0.57	1.19	1.59	1.00	0.84	-0.29	-1.31	-1.24
C2_28608	UPF0760 protein C2orf29	-0.16	-0.07	0.21	0.27	0.25	0.06	-0.08	-0.16	-0.14

Supplementary Table S3. Cycling genes showing major overlapping of canonical pathways (except for cluster 1) and playing key roles in metabolism and growth are putative biomarkers of circadian physiology and daily growth scope. The clone code for each gene in the Nutrigroup database (www.nutrigroup-iats.org/seabreamdb) is indicated. Correspondence for canonical pathways numbers is stated in Fig. 3b for cluster 2 genes, Fig. 4a for cluster 3 genes, and Fig. 4b for cluster 4 genes.

	Clone	Gene description	Canonical pathway	Function
Cluster 1 (suitable time to be measured: ZT0)				
1	C2_5902	UCP1	TR/RXR Activation	Regulates the production of ROS
2	C2_60770	UCP2	TR/RXR Activation	Regulates the production of ROS
3	C2_22601	UCP3	TR/RXR Activation	Regulates the production of ROS
Cluster 2 (suitable time to be measured: ZT3)				
4	C2_8602	Cyclin-dependent kinase 2 (*cdk2*)	4 5 6 7 8 9 10 11	Control of the cell cycle at G1-S phase transition.
5	C2_7311	Cyclin-dependent kinase 4 (*cdk4*)	2 4 5 6 8 9 10 11	Control of the cell cycle at G1-S phase transition.
6	C2_39274	G1/S-specific cyclin-D1	2 4 5 6 8 9 10 11	Regulatory subunit of D1-CDK4 complex during G1-S phase transition
7	C2_15396	Retinoblastoma-associated protein (*rb1*)	2 3 4 6 8 9 10 11	Negative regulator of G1-S phase transition. If phosphorylated by Cyclin D/CDK4 is unable to restrict entry into S phase.
8	C2_228	Proliferating cell nuclear antigen (*pcna*)	1 4 5 7	Auxiliary protein of DNA polymerase during DNA replication
9	C2_2839	Replication factor C subunit 3	1 2 3 7	Auxiliary protein of DNA polymerase during DNA replication
10	C2_9764	Replication factor C subunit 5	1 2 3 7	Auxiliary protein of DNA polymerase during DNA replication
11	C2_6841	Replication protein A 70 kDa DNA-binding subunit	1 2 3 7	Stabilizes single-stranded DNA during replication and prevents reannealing. Involved in response to DNA damage
12	FM148633	DNA mismatch repair protein Msh2	1 2 3	Initiate post-replicative DNA mismatch repair
13	C2_11938	Serine/threonine-protein kinase Chk1 (Checkpoint Kinase)	2 3 4 7 10	Central component DNA damage G1/S checkpoint. Delay cell cycle progression in response to DNA damages. Also participates in G2/M checkpoint and M phase
Cluster 3 (suitable time to be measured: ZT12)				
14	C2_1055	Cyclin-dependent kinase inhibitor 1	3 4 7 10 11 12	Plays a regulatory role in S phase DNA replication and DNA damage repair. Inhibits cellular proliferation at G1 in response to DNA damage or stress
15	C2_2037	Transcription factor Dp-1	4 10 11	Transcription factor that control the activity of numerous genes involved in G1-S phase transition
16	C2_75402	Cyclin-dependent kinase 1-B	2 3 4 7 8 10 12	Control of the cell cycle at G1-S and G2-M transition.
17	C2_4302	Cyclin-A2	4 10 11	Control of the cell cycle at G1-S and G2-M transitions
18	C2_3801	G2/mitotic-specific cyclin-B1	2 3 4 7 8 12	Control of the cell cycle at the G2-M transition.
19	C2_11108	G2/mitotic-specific cyclin-B2	2 3 4 7 8	Control of the cell cycle at the G2-M

				transition.
20	C2_18447	G2/mitotic-specific cyclin-B3	2 3 4 7 8	Control of the cell cycle at the G2-M transition.
21	C2_68747	Wee1-like protein kinase	2 3 4 12	Acts as a negative regulator of entry into mitosis (G2 to M transition).
22	C2_14888	78 kDa glucose-regulated protein (Heat shock protein 70 kDa family, Hsp70).	1 5 6 9	Facilitates the transport of newly synthesized proteins into the ER lumen and their subsequent folding. Found in all cell types but highly expressed in secreting cells like thyroid and pancreas
23	C2_15999	Heat shock 70 kDa protein 1 (Heat shock protein 70 kDa family, Hsp70)	1 6 9	Major role in protection against external stresses by avoiding the aggregation of denatured proteins
24	C2_213	DnaJ homolog subfamily A member 1 (Heat shock protein 40 kDa family, Hsp40)	1 9 13	Co-chaperone of Hsp70. Prevents aggregation of unfolded polypeptides. Involved in importing proteins into the mitochondria
25	C2_2999	DnaJ homolog subfamily B member 11 (Heat shock protein 40 kDa family, Hsp40)	1 9 13	Co-chaperone of Hsp70. Involved in folding of nascent proteins
26	C2_4773	DnaJ homolog subfamily C member 3 (Heat shock protein 40 kDa family, Hsp40)	1 5 6 9 13	Co-chaperone of Hsp70. Involved in folding of nascent proteins
27	C2_10236	DnaJ homolog subfamily C member 10 (Heat shock protein 40 kDa family, Hsp40)	1 9 13	Co-chaperone of Hsp70. Involved in the degradation of unfolded proteins
28	C2_5322	DnaJ homolog subfamily C member 17 (Heat shock protein 40 kDa family, Hsp40)	1 9 13	Co-chaperone of Hsp70. Involved in thyroglobulin transcription in thyroid. Important role in organogenesis and/or function of the thyroid
29	C2_1490	Endoplasmin (GRP-94) (Heat shock protein 90 kDa family, Hsp90)	1 2 5 6 9 11 14	Involved in the stabilization and folding of proteins in the secretory pathway in the ER
30	C2_4132	Heat shock protein HSP 90-alpha 1 (Heat shock protein 90 kDa family, Hsp90)	1 2 9 11 14	Aids in the proper folding of specific target proteins
31	C2_5798	Heat shock protein beta-8 (small heat-shock protein family, sHsps)	1 9 13	Blocks protein aggregation. Involved in regulation of cell proliferation and apoptosis. Induction of autophagy
32	C2_117130	Glutathione S-transferase	11 13	Oxidative Stress Response
Cluster 4 (suitable time to be measured: ZT21)				
33	C2_10954	Transcription factor E2F4	1 2 4	Transcription activator of genes whose products are involved in G1 to S transition and DNA replication
34	C2_91393	G1/S-specific cyclin-E1	1 2 4	Regulatory subunit of E1-CDK2 complex during G1 to S phase transition
35	C2_10260	M-phase inducer phosphatase 1	1 2 4	Required for G1 to S phase transition by activating CDK2 and CDK4
36	C2_7026	Ras-related protein R-Ras2	2 3 5	Activates signal transduction pathways that control cell proliferation
37	C3_lrc4088	Transcriptional regulator Myc-1-like	1 2 4 5	Transcriptional activator. Involved in cell proliferation and growth, but blocks differentiation.

38	C2_8684	Integrin alpha-5	2 3 5	Part of the receptor for fibronectin, needed for cell adhesion, growth, migration, and differentiation
39	C3_C57938	Pro-Neuregulin-4, Membrane-Bound Isoform (*nrg4*)	3 5	Activate type-1 growth factor receptors to initiating cell-to-cell signaling
40	C2_28111	Pro-neuregulin-1, membrane-bound isoform (*nrg1)*	1 3 5	Ligand for integrins. Mediates cell-cell signaling, growth and development of multiple organs. Induces expression of AChR in synaptic vesicles during neuromuscular development.
